# The RIG-I receptor adopts two different conformations for distinguishing host from viral RNA ligands

**DOI:** 10.1016/j.molcel.2022.09.029

**Published:** 2022-10-21

**Authors:** Wenshuai Wang, Anna Marie Pyle

**Affiliations:** 1 Department of Molecular, Cellular and Developmental Biology, Yale University, New Haven, CT 06511, USA; 2 Howard Hughes Medical Institute, Yale University, New Haven, CT 06520, USA; 3 Lead contact

## Abstract

RIG-I is an essential innate immune receptor for detecting and responding to infection by RNA viruses. RIG-I specifically recognizes the unique molecular features of viral RNA molecules and selectively distinguishes them from closely-related RNAs abundant in host cells. The physical basis for this exquisite selectivity is revealed through a series of high-resolution cryo-EM structures of RIG-I in complex with host and viral RNA ligands. These studies demonstrate that RIG-I actively samples double-stranded RNAs in the cytoplasm and distinguishes them by adopting two different types of protein folds. Upon binding viral RNA, RIG-I adopts a high-affinity conformation that is conducive to signaling, while host RNA induces an autoinhibited conformation that stimulates RNA release. By coupling protein folding with RNA binding selectivity, RIG-I distinguishes RNA molecules that differ by as little as one phosphate group, thereby explaining the molecular basis for selective antiviral sensing and the induction of autoimmunity upon RIG-I dysregulation.

## eTOC Blurb

Wang et al. show that RIG-I uses a domain unfolding event to bind strongly with target viral ligands and an alternative, enzymatically active conformation for sensing and discarding inappropriate host ligands, thereby explaining the molecular basis for selective antiviral sensing and the induction of autoimmunity upon RIG-I dysregulation.

## Graphical Abstract

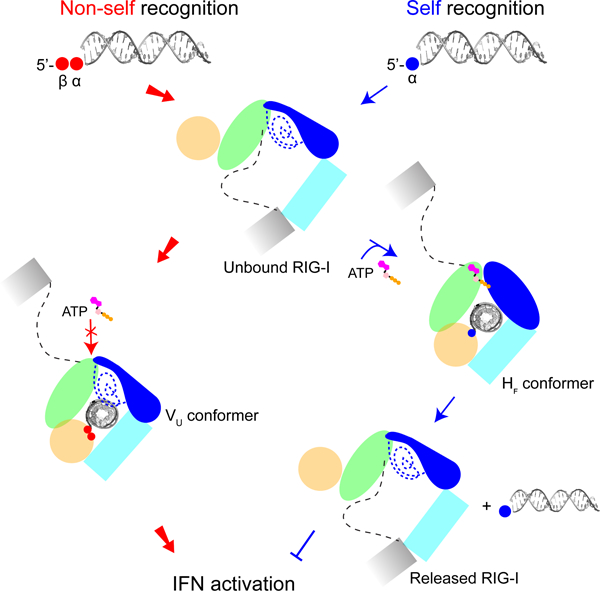

## INTRODUCTION

One of our most important cellular defenses against RNA virus infection is an innate immune sensor known as RIG-I (Retinoic Acid Inducible Gene-I) (Barrat et al., 2016; Cesaro and Michiels, 2021; Li and Wu, 2021; Taschuk and Cherry, 2020; Thoresen et al., 2021). This multidomain protein is essential for effective host response to the most significant viral health threats impacting society today, including coronaviruses, influenza, flaviviruses, and many more (Chen et al., 2016; Chiale et al., 2022; Diamond and Kanneganti, 2022; Fitzgerald et al., 2014; Madden and Diamond, 2022). Understanding the molecular events that guide RIG-I activation and response is central to the development of new therapeutic strategies that aim to harness the great potential of the human innate immune response against viral infection, cancer and other diseases.

RIG-I displays exquisite sensitivity in recognizing minute quantities of viral RNA in the cytoplasm and then forming a tight RNA:protein complex that releases specialized signaling domains (CARDs, or Caspase Recruitment Domains), which engage in a cascade of downstream interactions leading to induction of interferons and a rapid antiviral immune response (Kolakofsky et al., 2012; Thoresen *et al.*, 2021; Yoneyama et al., 2005; Yoneyama et al., 2004). While each step in this process is critical, the entire response rests on the specificity and sensitivity of the initial trigger: RIG-I recognition and conformational response to viral RNA. Years of work by many labs has shown that the most potent triggers of the RIG-I response in biochemical experiments, in cells and in living animals are double-stranded RNAs (dsRNAs) containing a blunt, base-paired terminus that is decorated with a 5’-triphosphate (p3dsRNA) or 5’-diphosphate (p2dsRNA), which are prevalent on viral genomic RNAs and replication intermediates (Baum et al., 2010; Chiang et al., 2015; Goubau et al., 2014; Goulet et al., 2013; Linehan et al., 2018; Liu et al., 2015; Schlee et al., 2009; Schmidt et al., 2009; Te Velthuis et al., 2018). While these dsRNAs can be of any length, short dsRNAs (10–20 base-pairs), such as those found in a genomic viral RNA panhandle, are particularly potent RIG-I agonists (Kohlway et al., 2013; Linehan *et al.*, 2018; Liu *et al.*, 2015).

But just as RIG-I must rapidly and faithfully recognize trace quantities of viral RNA, it must ignore the sea of host RNAs that circulate within the cytoplasmic environment. Abundant dsRNA molecules containing 5’-cap structures, 5’-monophosphate groups, 5’-hydroxyls, and a diversity of blunt, and overhanging dsRNA molecules surround the RIG-I receptor at all times. How does RIG-I become selectively activated by trace amounts of viral dsRNAs while remaining dormant within an abundant pool of host RNAs that differ little from their viral counterparts? The selectivity for viral RNAs rests on an extremely narrow set of molecular determinants. For example, the most potent RIG-I agonists are short, 5’-diphosphorylated RNAs (p2dsRNAs), while the most potent antagonists are 5’-monophosphorylated RNAs (p1dsRNAs). Avoiding p1dsRNAs is particularly critical given that microRNAs are frequently presented as uncapped, 5’-monophosphorylated dsRNA molecules (Ghildiyal and Zamore, 2009; Park et al., 2011). Abundant cellular p1dsRNAs also include mRNA degradation and triphosphorylase intermediates (Braun et al., 2012; Choi et al., 2020; Choi and Sullivan, 2021; Jonas and Izaurralde, 2013; Nagarajan et al., 2013; van Dijk et al., 2002). The extreme, opposite responses to p2dsRNAs and p1dsRNAs indicate that RIG-I can sense the presence of a viral RNA by detecting a single chemical group: the β phosphate of p2dsRNAs and p3dsRNAs (Ren et al., 2019). One phosphate residue makes the difference between a resting cell and one that is churning out interferon (Ren *et al.*, 2019). While we have conducted biochemical studies designed to understand this razor-edge specificity, a structural explanation has been lacking.

Until now, our existing knowledge of RIG-I conformational states and RNA interactions has stemmed from a small set of crystal structures that were obtained under diverse conditions, using RIG-I truncation constructs employed in different laboratories, making it difficult to compare features that enable RIG-I to differentiate host and viral RNAs (Devarkar et al., 2016; Jiang et al., 2011; Kohlway *et al.*, 2013; Kowalinski et al., 2011; Luo et al., 2011; Luo et al., 2012). Despite the importance of these structures, available data on RIG-I ligand interactions remained incomplete, as RIG-I was never captured in complex with p1dsRNA, or p2dsRNA, nor at an internal dsRNA site. As a result, structural understanding of host-pathogen specificity has remained elusive.

To uncover the molecular basis for RIG-I ligand specificity, we created a pipeline for solving single-particle cryo-EM structures of full-length, human RIG-I protein in complex with a diversity of dsRNA molecules that represent different types of viral and host RNA ligands. Using this approach, we have obtained a complete collection of high-resolution RIG-I host and viral complexes, capturing RIG-I in complex with its most biologically significant agonist (p2dsRNA) and antagonist (p1dsRNA) ligands for the first time. By visualizing these and many other complexes, including those obtained in the presence of ATP, we reveal that RIG-I has multiple, layered strategies for differentiating host from viral RNA. We find that RIG-I uses two different conformations to recognize host and viral ligand classes, and that the specificity of RNA ligand binding is amplified by coupling it with distinct protein folding events that guide selectivity and downstream behavior of the protein. The resulting set of structural data, combined with mutational functional analysis, provide a coherent, unifying mechanism that explains the exquisite selectivity of this critical antiviral receptor and the molecular basis for its dysregulation in autoimmune disease.

## RESULTS

### High resolution structural differentiation of RIG-I host and viral dsRNA complexes using single-particle cryo-electron microscopy.

To elucidate the structural basis for RIG-I discrimination between host and viral RNA ligands, and to visualize dsRNA:RIG-I structures in solution, we set out to solve high-resolution structures of full-length RIG-I receptor (RIG-I) bound to four different types of blunt-ended dsRNA duplexes: 5’-triphosphorylated dsRNA (p3dsRNA) and 5’-diphosphorylated dsRNA (p2dsRNA), 5’-monophosphorylated dsRNA (p1dsRNA) and 5’-OH-dsRNA (OHdsRNA). While p3dsRNA and p2dsRNA represent ligands that mimic high affinity viral PAMPs, p1dsRNA and OHdsRNA represent classes of RNA that are abundant in the host cytoplasm (Ren *et al.*, 2019). RIG-I must trigger IFN signaling upon binding the former “viral” dsRNAs and remain silent upon encountering the latter, more abundant category of “host” dsRNAs. Given potential subtleties in molecular recognition strategies for these two families, we first set out to develop a pipeline for reproducible particle preparation and single-particle reconstruction that would enable us to ultimately compare different types of complexes solved under identical solution conditions.

Having developed a unified sample preparation and imaging pipeline (See Methods), we obtained all four complexes in parallel at good overall resolution, resulting in p3dsRNA:RIG-I, p2dsRNA:RIG-I, p1dsRNA:RIG-I and OHdsRNA:RIG-I complexes resolved at 3.5 Å, 3.2 Å, 3.5 Å and 3.5 Å respectively (Fig S1, Fig S2A–S2D, Fig S3A–S3D, S4A–S4D and S5A–S5D; Table S2). Given that previous RIG-I structural studies were performed using crystallography on truncated complexes, this is the first time RIG-I dsRNA complexes were visualized in solution, using full-length protein. In addition, these represent the first structures of RIG-I in complex with 5’-pp and 5’-p RNA duplexes, which represent the most important viral and host PAMP termini, respectively. After successfully building atomic models into the EM maps (Fig S2E, S3E, S4E and S5E; Table S2), it was possible to visualize and analyze molecular interactions and compare structural features of these four complexes (S2G–S2H, S3G–S3H, S4G–S4H and S5G–S5H). We observe striking differences between the complexes bound to “viral” ligands (with p2dsRNA or p3dsRNA), and those bound to “host” ligands (p1dsRNA or OHdsRNA), but certain architectural features are similar amongst all four complexes, as described below.

### Shared architectural features of RIG-I complexes with host and viral dsRNA ligands.

**Figure 1.**
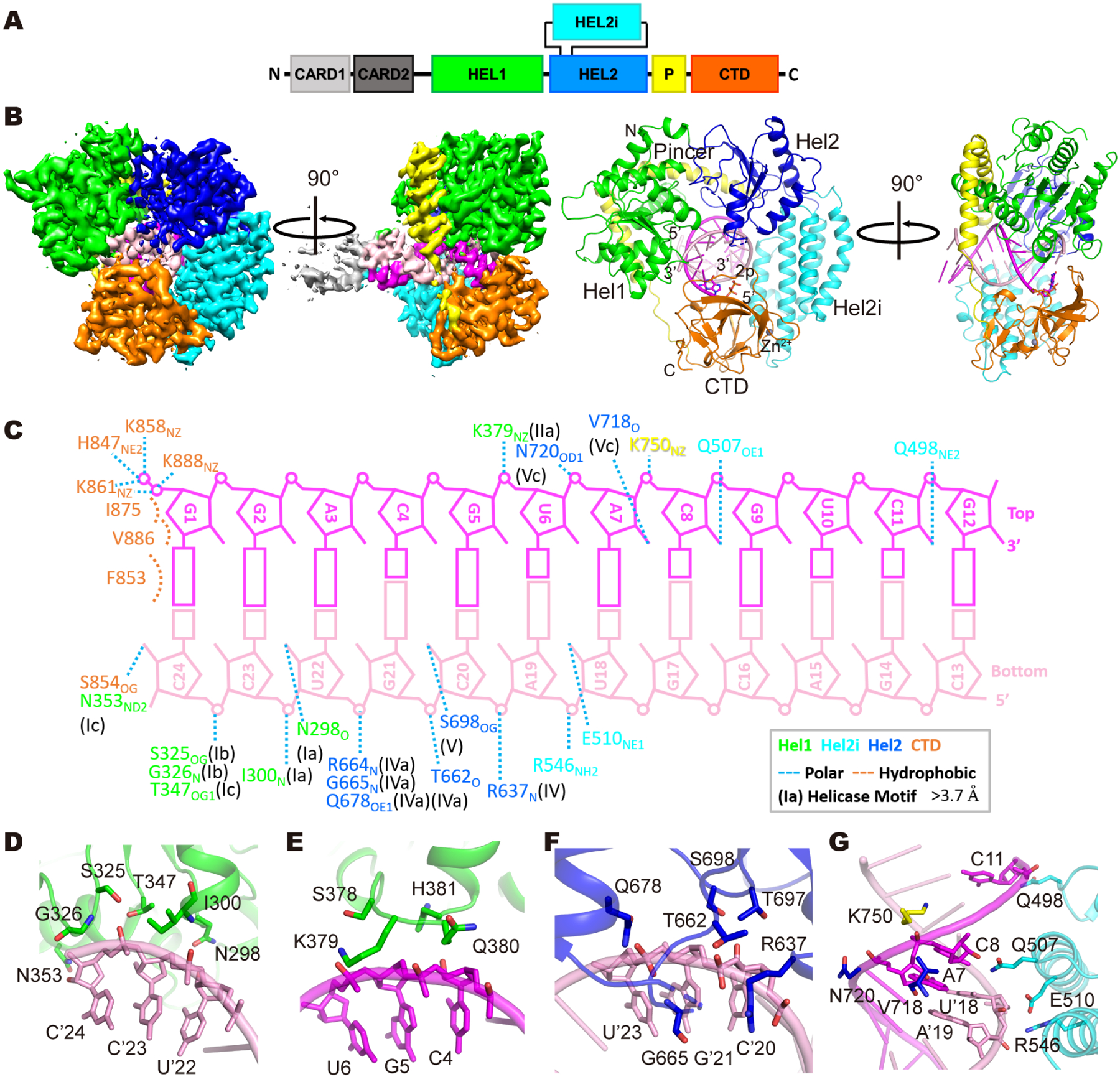
Shared structural features of the dsRNA:RIG-I complexes. (A) Schematic representation of the RIG-I protein. (B) Cryo-EM map and overall structure of a representative complex (p2dsRNA:RIG-I). Starting from the N terminus: The CARDs are invisible in the structure; Hel1 (green), Hel2i (cyan), Hel2 (blue), Pincer (yellow) and CTD (orange) of RIG-I are highlighted. The top and bottom strands of the dsRNA is colored in magenta and pink, respectively. The diphosphate is shown as sticks. (C) The interactions between p2dsRNA and RIG-I, determined with a 3.7 Å cutoff for polar contacts. (D-G) Zoom-in views of Hel1-RNA (D and E), Hel2-RNA (F) and Hel2i-RNA (G) interfaces in p2dsRNA:RIG-I complex. See also Figure S1, S2, S3, S4, S5, S6, S7 and S8, Table S1, S2 and S3

As observed in previous crystallographic studies, all complexes visualized by cryo-EM reveal a familiar ring-like structure in which the domains of RIG-I are wrapped around dsRNA and the blunt duplex terminus is capped by stacking and hydrogen-bonding networks with the protein (Fig 1, S6 and S7) (Jiang *et al.*, 2011; Kohlway *et al.*, 2013; Luo *et al.*, 2011; Luo *et al.*, 2012). This observation establishes that the overall architecture and RNA recognition strategy of RIG-I is maintained in the solution environment.

The N-terminal RecA fold known as Helicase domain 1 (Hel1) forms specific contacts with backbone residues on the “bottom” strand (3’-end) of the duplex terminus. Together with Hel1, the second RecA fold (Hel2), forms a cleft that contains conserved motifs critical for RNA-dependent ATP binding and hydrolysis (Fig 1B, 1C, 1D, 1E, 1F, 1G, S6B, S7 and S8). Unique to RLRs, the V-shaped Pincer domain is critical for RLR functionality as it connects and coordinates action of Hel1, Hel2 and the CTD (Rawling et al., 2014), and in this set of structures we observe that it forms a direct contact with the RNA at position C8 (via 750, Fig 1G and S8).

The alpha-helical bundle inserted into Hel2 (Hel2i) is a unique feature of RLRs and metazoan Dicer proteins. This domain engages with 2’-hydroxyl groups on both strands of the dsRNA (Fig 1B, 1C, 1G, S6B, S7 and S8). In addition to conferring dsRNA binding capability to an otherwise canonical SF2 protein (most of which bind ssRNA), the Hel2i domain plays a critical role in sequestering the CARDs via an interaction interface on the outer surface of the RIG-I protein. When RIG-I is unbound (the apo form, Fig S9A), the alpha-helical CARDs are sequestered against a specific recognition site along the surface of Hel2i, forming an autoinhibitory interaction that prevents the initiation of signaling (Kowalinski *et al.*, 2011). Although the Caspase Recruitment Domains (CARDs) were included in the RIG-I construct that was used for these cryo-EM studies, the CARDs became dynamic upon RNA binding and could not be visualized. In the dsRNA:RIG-I structures, the CARDs of RIG-I are unable to pack against Hel2i because their binding site has become blocked by the position of the CTD (vide infra). This indicates that RIG-I binding to any dsRNA terminus, regardless of its identity, is sufficient for releasing the CARDs into solution, as reported in earlier FRET studies of CARD dynamics (Fig 1B and S6B) (Dickey et al., 2019).

The C-terminal domain (CTD) is critical for distinguishing host from viral RNA, and in all complexes, it is anchored via hydrophobic interactions with the base-paired RNA terminus (Fig 1C and S7). From this position, the CTD can sense the differences between host and viral dsRNA by making different types of contacts to 5’-terminal substituents (vide infra). When the CTD stacks on the terminal base pair of the dsRNA duplex, it blocks re-entry of the ejected CARDs.

### **Structural features that distinguish host and viral dsRNA complexes with RIG-I**.

A comparative analysis of the four dsRNA:RIG-I complexes shows that they fall into two distinct architectural categories. Complexes formed between RIG-I and dsRNAs that are abundant in the host (5’-hydroxyl or 5’-monophosphate RNA (OHdsRNA and p1dsRNA)) adopt one type of conformation, while complexes formed between RIG-I and dsRNAs typical of viral RNAs (5’-di- or triphosphate RNAs (p2dsRNA and p3dsRNA) form a different type of conformation. The grouping of these conformational isoforms is now possible because RIG-I structures with p1dsRNA and p2dsRNA are being reported here for the first time, and all four structures were solved under identical conditions, enabling a head-to-head comparison. As described in the subsequent sections, the primary differences between the host and viral conformations of RIG-I are attributable to changes in the CTD and Hel2.

#### Characteristic features of RIG-I complexes with 5’-p and 5’-OH dsRNAs (HostF conformer)

**Figure 2.**
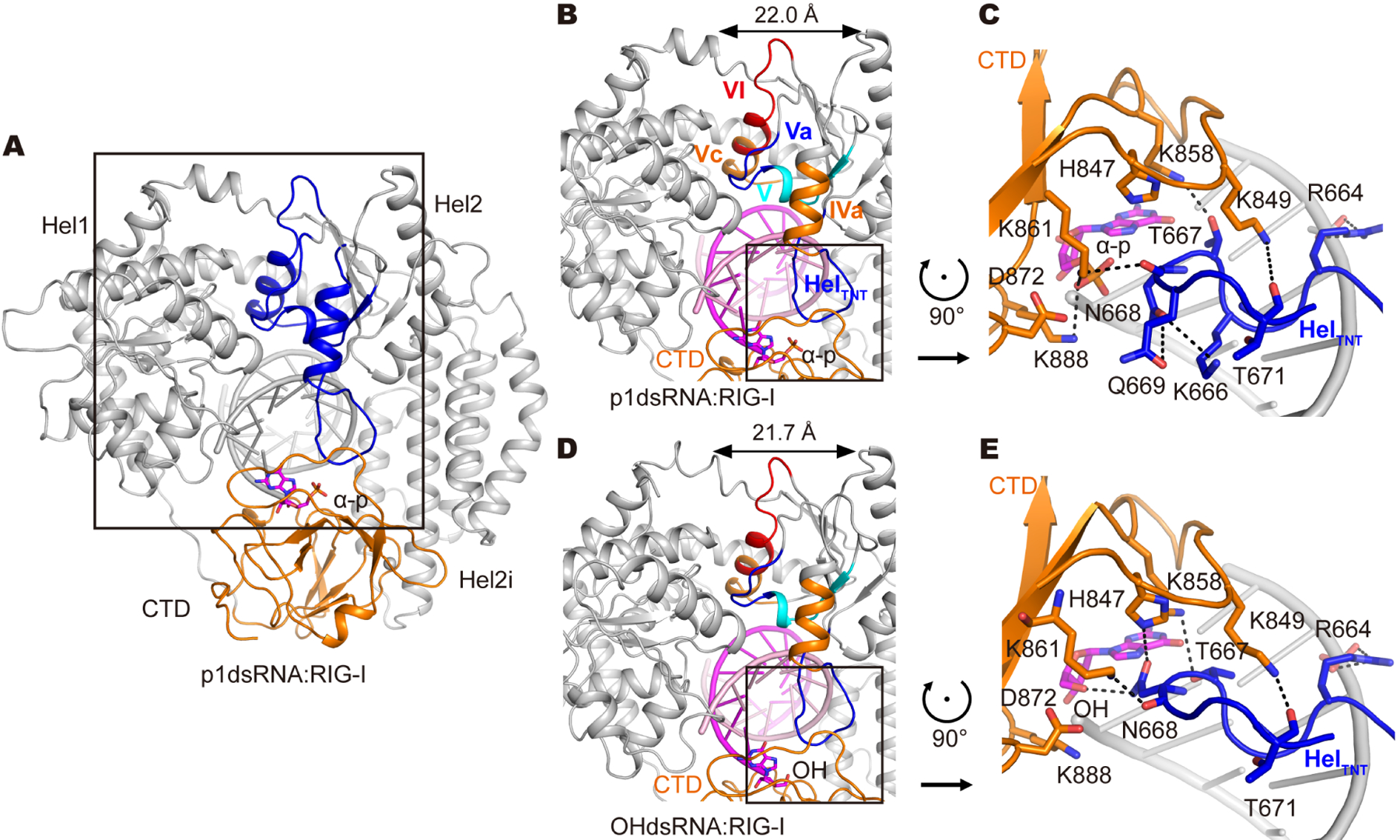
Distinguishing structural features of RIG-I in complex with host RNA duplexes. (A) Overall structure of p1dsRNA:RIG-I. The well-folded helicase motifs in Hel2 are highlighted in blue. The α-p is rendered as sticks. (B, D) Zoom-in views of the Hel2 domain in the p1dsRNA:RIG-I complex (B) and the OHdsRNA:RIG-I complex (D). Helicase motifs are highlighted as follows: Heltnt (blue), motif IVa (orange), V (cyan), Va (blue), Vc (orange) and VI (red). The first nucleotide of the 5’ terminus is rendered as sticks. The distances between Hel1 and Hel2 are denoted. (C, E) CTD-RNA-Heltnt interface in the phosphate binding pocket within CTD in the p1dsRNA:RIG-I complex (C) and the OHdsRNA:RIG-I complex (E). CTD, Heltnt and the first nucleotide of the 5’ terminus are rendered in orange, blue and magenta, respectively. The polar contacts are denoted, and the corresponding residues are rendered as sticks. See also Figure S9, S10 and S11

A distinctive feature of the “host RNA” complexes is that the helicase motifs in Hel2 are all well folded (this conformation of RIG-I is therefore designated as HostF (HF); Fig 2A), which contrasts sharply with the relatively unfolded arrangement of Hel2 in apo-RIG-I structures (Fig S9A and S9B). Like other SF2 helicases in complex with RNA, the Hel2 of RIG-I has α/β/α three-layered sandwich architecture with a twisted parallel β-sheet bounded by five α-helices (Fig S9C and S9D). The helicase motifs (motif IVa, V, Va, Vc and VI) lie within three α-helices that pack against the β-sheet and interact with RNA, forming a well-ordered set of Hel2 secondary structures in RIG-I complexes containing p1dsRNA and OHdsRNA (HF complexes, Fig 2B, 2D, S10A and S10B). This organization is significant because active SF2 helicases require a well-folded conformation of motif IVa, and this region must be closely connected with the two adjacent β-strands in order to maintain the appropriate interaction interface with adjacent α-helical motifs. But in the case of RIG-I, maintaining the fold of Hel2 is challenging because the domain contains unusual insertion motifs that reduce its structural integrity. As we will show below, this serves a critical function in regulating protein function.

In addition to the Hel2i alpha-helical bundle, Hel2 contains a second insertion motif that becomes ordered by intercalating into the central pocket of the CTD, where it forms a network of interactions (Fig 2B and 2D, blue loop). In RIG-I, this unusual inter-domain interaction serves to preorganize motif IVa in an orientation that stabilizes its interactions with the other secondary structural motifs (described above), enabling Hel2 to adopt a canonical SF2 fold (Fig 2B and 2D). This unusual Hel2 insertion (with sequence N_664_-RGK**TN**QN**T**G-C_672_, namely HelTNT) docks within the CTD. Remarkably, as described below, HelTNT interacts with the exact same set of CTD atoms that are also contacted by the 5’-diphosphate of pathogen RNAs, suggesting that HelTNT has evolved as an autoinhibitory motif.

It is interesting that, in the OHdsRNA:RIG-I complex, N668 of HelTNT interacts tightly with the 5’-OH group of the RNA terminus and H847 of the CTD (Fig 2E); while in the p1dsRNA:RIG-I complex, K861 and K888 of the CTD interact with the α-phosphate, resulting in a clash between the side chain of N668 and the α-p, and causing the N668 side chain to adopt a different conformation (Fig 2C). This does not lead to a disordered HelTNT, but it may make the HelTNT less stable, as supported by poorer EM density of Heltnt in p1dsRNA complex (Fig S10C). This apparent disruption of H-bond interactions in the CTD binding pocket may explain the reduced binding affinity of RIG-I to 5’-p RNA duplexes relative to 5’-OH RNA duplexes (Ren *et al.*, 2019).

Buttressed by the HelTNT-CTD interface, Hel2 adopts a relatively conventional SF2 fold in the HF complexes, making it possible to compare this structure with that of other SF2 proteins. It is important to note that well-folded SF2 proteins sample two closely-related conformations (open and closed), which differ in the distance between Hel1 and Hel2 (Appleby et al., 2011; Hamann et al., 2019). Intriguingly, both cryo-EM HF complexes of RIG-I are in the SF2 “closed” conformation, even in the absence of ATP (Fig 2B, 2D and S11). However, it is likely that this conformer is capable of binding ATP because the ATP interaction cleft is well-organized and because previous crystallographic studies report a RIG-I-dsRNA-ADPBeF_3_ complex in the SF2 closed conformation (Fig S11A) (Jiang *et al.*, 2011). Whether Hel1 and Hel2 can slightly flex and form the SF2 “open” complex to release ATP is not yet clear. However, the close architectural similarity between DEAD-box proteins and the RIG-I HF complex, along with robust RNA-dependent ATPase activity observed for RIG-I (Rawling et al., 2015), indicates that the HF conformation can undergo cycles of ATP binding and hydrolysis that are linked to RNA bindingand release, as observed previously in pre-steady-state kinetic experiments (Devarkar et al., 2018; Rawling *et al.*, 2015).

#### Characteristic features of RIG-I complexes with 5’-ppp and 5’-pp dsRNAs (ViralU conformer)

**Figure 3.**
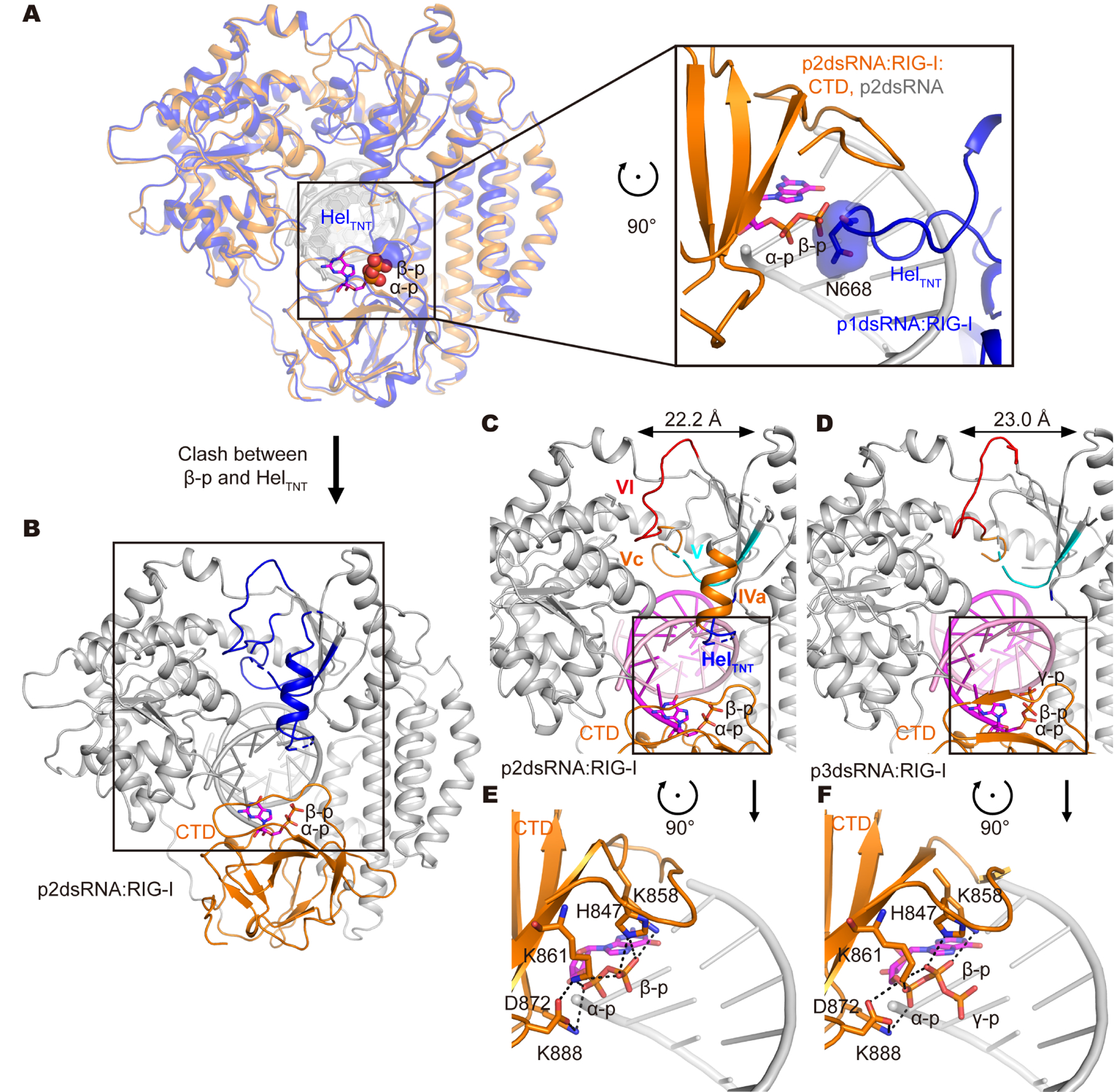
Distinguishing features of RIG-I in complex with viral RNA duplexes. (A) Overlay of p1dsRNA:RIG-I and p2dsRNA:RIG-I. The p1dsRNA:RIG-I in blue and p2dsRNA:RIG-I in orange are illustrated. The α and β-p are rendered as red spheres, while N668 is shown as a transparent surface. Zoom-in view shows the clash between β-p and Heltnt. The α and β-p are rendered as sticks, while N668 is shown in sticks and in transparent surface. (B) Overall structure of p2dsRNA:RIG-I. The disordered helicase motifs in Hel2 are highlighted as blue. The α, β-p are rendered as sticks. (C-D) Zoom-in views of the Hel2 domain in the p2dsRNA:RIG-I complex (C) and the p3dsRNA:RIG-I complex (D). Helicase motifs are highlighted as follows: Heltnt (blue), motif IVa (orange), V (cyan), Va (blue), Vc (orange) and VI (red). The first nucleotide of the 5’ terminus is rendered as sticks. The distances between Hel1 and Hel2 are denoted. (E-F) CTD-RNA interface in the phosphate binding pocket within CTD in the p2dsRNA:RIG-I complex (E) and the p3dsRNA:RIG-I complex (F). CTD and the first nucleotide of the 5’ terminus are highlighted in orange and magenta, respectively. The polar contacts are denoted, and the corresponding residues are rendered as sticks. See also Figure S10, S11 and Movie S1

The most remarkable feature of RIG-I bound to p2dsRNA and p3dsRNAs is that insertion of the 5’-terminal β phosphate into the CTD prevents docking of the HelTNT motif (Fig 3A), and when HelTNT is disengaged from its supporting scaffold within the CTD, it triggers the catastrophic unfolding of Hel2, which effectively “blows up” due to loss of stabilizing interactions between Hel2 secondary structural motifs (Fig 3B; Movie S1). Because of this effect, we have designated RIG-I complexes with p2dsRNA and p3dsRNA the “Viral Unfolded” or ViralU (Vu) conformation. An overlay of the Vu and HF conformers shows that the position of the β-p within the CTD is incompatible with HelTNT insertion, resulting in a disordered HelTNT (Fig 3A) that fails to stabilize motif IVa in the proper orientation, making it impossible to maintain the folding of alpha-helical motifs in Hel2 (Fig 3B). This in turn melts the Hel2 ATP binding motifs (IVa, V, Va, Vc and VI), which must be precisely positioned to form a functional ATP binding cleft within any SF2 protein (Fig 3C, 3D and S10D). Importantly, the Vu conformation is not analogous to the “SF2 open conformation”, which maintains a well-folded Hel2 that is simply held a little farther away from Hel1 (Fig 3C, 3D and S11B). Rather, Vu is a distinct conformation that may be unique to RLR proteins and which does not appear capable of functional ATP binding. Any apparent ATP binding by end-bound RIG-I molecules is likely to be the result of dynamic sampling of a “Hel2 folded” state, and the reformation of Motif IVa, which cannot occur unless the dsRNA 5’-diphosphate disengages from the CTD (Movie S1). An intriguing consequence of the Vu conformation is that, while molecular contacts with ATP are disrupted, specific interactions with viral RNA become greatly enhanced (Table S3). Indeed, the RNA recognition interface expands to include not just the helicase domain, but the RIG-I CTD, which tightly grips the diphosphorylated terminus in a complex network of close interactions.

A second feature that distinguishes complexes in the Vu conformation from those in the HF conformation is that the CTD is closer to Hel2i. This results in an extensive interaction interface between CTD and Hel2i that is expected to completely block reassociation of the CARDs and prevent re-formation of the autoinhibited conformation seen in the apo state. A comparison of Vu p2dsRNA:RIG-I and HF p1dsRNA:RIG-I complexes reveal that CTD in Vu makes more contacts with Hel2i (polar contacts: 9 versus 3; total contacts: 171 versus 140) and buries a larger surface with Hel2i (1340.7 Å^2^ versus 1245.9 Å^2^) by tilting about 1 Å towards Hel2i. Taken together, these findings suggest that the CTD binds tighter to Hel2i in the p2dsRNA complexes in Vu conformation, which will favor signaling over autoinhibition relative to the p1dsRNA complex in HF conformation.

Features of the p2dsRNA:RIG-I complex are being presented here, for the first time. While crystallographic studies have resulted in several p3dsRNA:RIG-I structures, p2dsRNA:RIG-I structures have not been available until now, probably due to the lack of commercially-available 5’-diphosphate RNA ligands. Much like 5’-triphosphorylated ligands, α and β phosphates of the diphosphate linkage bind within the “recognition” pocket of the CTD, forming a network of interactions with highly conserved amino acids. A comparison of the p2dsRNA:RIG-I and p3dsRNA:RIG-I structures establishes that the RIG-I CTD interacts with both α and β phosphates in both cases, but interactions are not formed with the γ-p in the case of p3dsRNA, as observed in our previous crystallographic investigation of this complex (Fig 3E and 3F) (Luo *et al.*, 2012). K858 was previously reported to interact with the γ-p in one available crystal structure, but this is likely to be attributable to poorly-defined electron density for the K858 side chain in the electron density map (Devarkar *et al.*, 2016). Taken together, these data establish that the β phosphate of viral dsRNA duplexes is the primary molecular determinant for RIG-I recognition and signaling.

### Functional validation of the Vu and H_F_ conformations

To evaluate whether the Vu and H_F_ conformations are functionally linked with IFN signaling and RIG-I recycling, respectively, we designed mutants that favor one conformation over the other, and we tested the resulting impact on IFN activation using host and viral dsRNA ligands.

**Figure 4.**
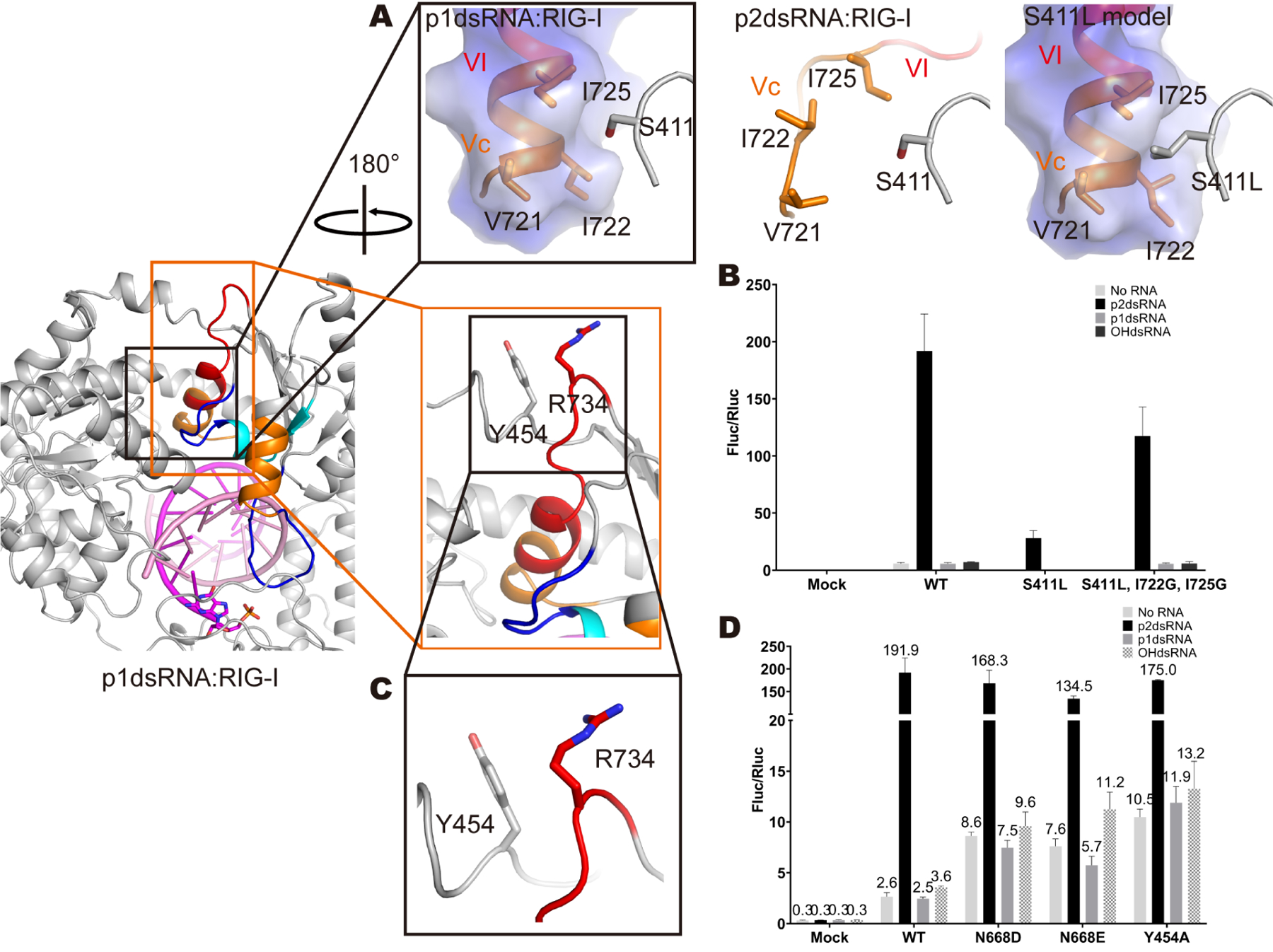
Functional validation of the Vu and HF conformations. (A) Zoom-in views of motif III, Vc and VI in p1dsRNA:RIG-I, p2dsRNA:RIG-I and S411L modeling complexes. Motif III, Vc and VI are highlighted as gray, orange and red, respectively. The electrostatic surface of motif Vc and VI in p1dsRNA:RIG-I and S411L model is shown. The residues are rendered as sticks. (B) IFN-β induction by RIG-I mutants stimulated with p2dsRNA, p1dsRNA and OHdsRNA in HEK293T Cells. Mutants validating the Vu conformation were tested. Data are represented as mean ± SD (n=3). (C) Zoom-in view of an α-helix formed by motif Vc and VI. Motif Vc and VI are highlighted as orange and red, respectively. Y454 stacking with R734 is shown in gray. The residues are rendered as sticks. (D) IFN-β induction by RIG-I mutants stimulated with p2dsRNA, p1dsRNA and OHdsRNA in HEK293T Cells. Mutants validating the HF conformation were tested. Data are represented as mean ± SD (n=3). See also Figure S12

First, we studied a mutant that is expected to lock RIG-I in the H_F_ conformation and we examined its ability to signal in the presence of host and viral dsRNA ligands, expecting that it would have impaired signaling in all cases. With stabilized motifs Vc and VI, RIG-I complexed with p3dsRNA is forced to adopt the HF conformation (Fig S12) (Devarkar *et al.*, 2016). The signal dead mutant, S411L (Fitzgerald et al., 2017; Forbes et al., 2011; Kent et al., 2002; Landrum et al., 2016), is expected to stabilize Hel2 by packing against the hydrophobic pocket in Vc and VI. By employing molecular modeling of S411L within HF structures determined in this study, we found that S411L is positioned to rigidify the α-helix containing motif Vc and VI, creating an extensive hydrophobic core within Hel2 that would be expected to permanently enforce the HF conformation (Fig 4A). In turn, this would be likely to block dynamic sampling of the Vu conformation and explains why the S411L mutant cannot signal upon stimulation with p3dsRNA ligands. To test this idea, we designed an intragenic suppressor of S411L by mutating residues I722 and I725 in the hydrophobic pocket, aiming to disrupt the network of interactions with S411L, thereby compromising the structural environment around motifs Vc and VI, and tilting the conformational balance to Vu. Indeed, consistent with the model, the suppressor mutations rescued RIG-I signaling activity in the presence of viral RNA duplexes (Fig 4B). These results demonstrate that rigidifying the RIG-I helicase core through the hydrophobic effect can freeze the protein in the HF conformation, which has low affinity for p3dsRNA ligands in the presence of ATP (Fitzgerald *et al.*, 2017).

We then designed a series of mutants expected to favor the Vu conformation, examining the resulting impact on IFN induction by dsRNA ligands. The first mutant was based on the observation that N668 is a key residue orienting the position of Heltnt (Fig 2C and 2E). By disrupting these interactions, N668A/D/E mutants tilt the balance towards Vu, which is expected to activate IFN response, even in the presence of inappropriate host RNA duplexes. As expected, the N668A mutant enables RIG-I to moderately respond to 5’-p and 5’-OH RNA duplexes, and N668D/E mutants are moderately activated by endogenous host RNA, p1dsRNA and OHdsRNA as well (Ren *et al.*, 2019) (Fig 4D). Another mutant was designed by noting that, in the HF conformation, Y454 and R734 form a set of hydrophobic interactions that help maintain the helical structure of motifs Vc and VI (Fig 4C). By disrupting these interactions, an Y454A mutant was expected to destabilize the HF conformation and favor the Vu conformation, even in the presence of host RNAs. Consistent with this, Y454A is moderately activated by endogenous host RNA, p1dsRNA and OHdsRNA (Fig 4D). Taken together, these data establish that Hel2 rigidification favors the HF conformation, which prevents signaling; and Hel2 destabilization favors the Vu conformation, which stimulates signaling. When Vu is inappropriately stabilized through mutation, RIG-I can signal moderately on host dsRNAs, thereby establishing a molecular mechanism for certain classes of genetic interferonopathies.

### Alternative RIG-I structural states formed in the presence of ATP

**Figure 5.**
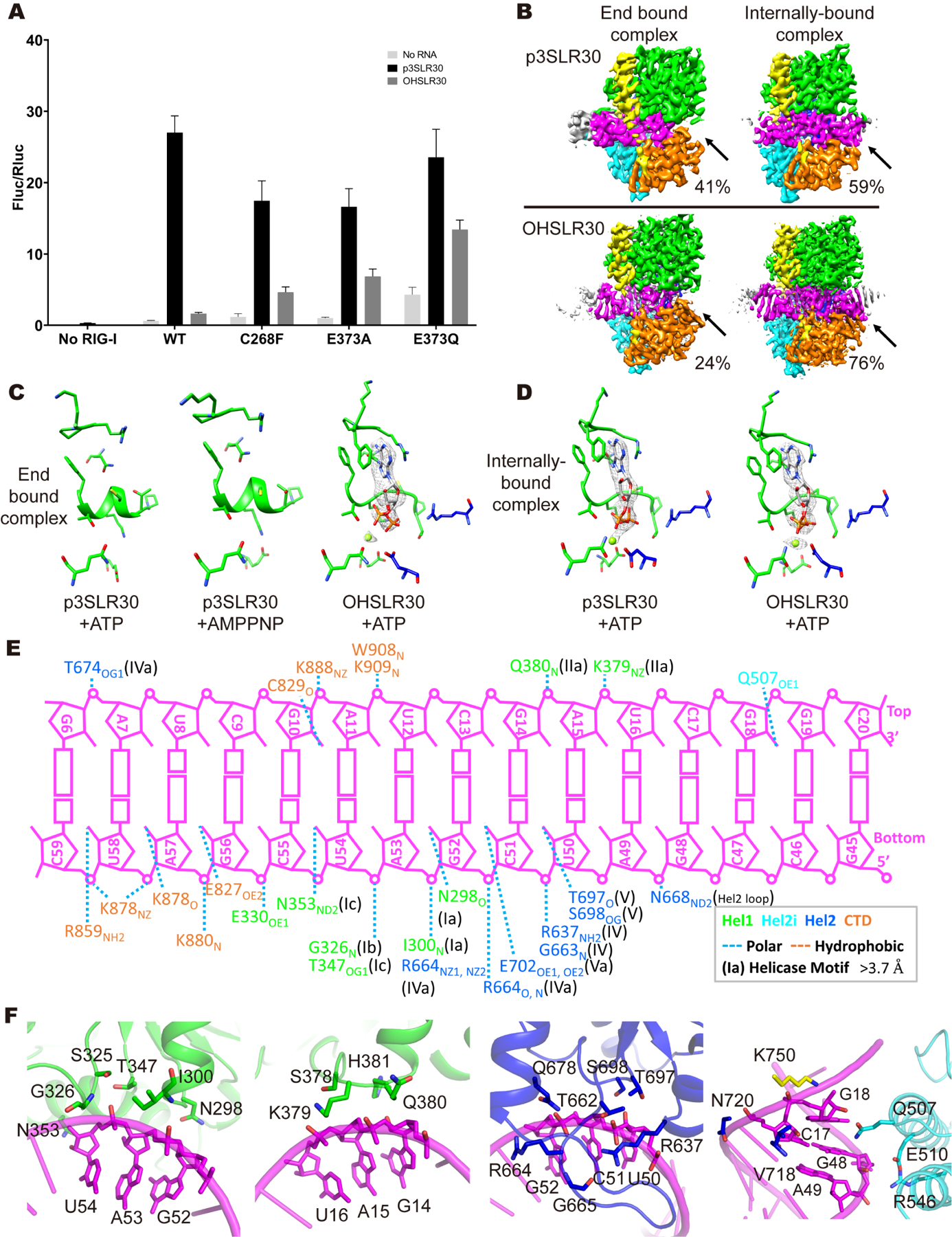
Characteristics of the end-bound and internally-bound complexes. (A) IFN-β induction by RIG-I mutants stimulated with p3SLR30 and OHSLR30 in HEK293T Cells. ATP-deficient and translocation-deficient mutants were tested. Data are represented as mean ± SD (n=3). (B) Cryo-EM maps of p3SLR30 end bound, internally-bound complexes and OHSLR30 end bound, internally-bound complexes. Positions of CTD and RNA highlighted in orange and magenta are indicated by arrows. The percentage of final particles used to reconstruct the maps is presented. (C-D) ATP binding site of p3SLR30, OHSLR30 end bound complexes (C) and p3SLR30, OHSLR30 internally-bound complexes (D). ADP, Mg^2+^ and interacting residues are shown. The corresponding density of ADP and Mg^2+^ is rendered as mesh. Residues of Hel1 and Hel2 are rendered in green and blue, respectively. (E) The interactions between stem of p3SLR30 and RIG-I, determined with a 3.7 Å cutoff for polar contacts. (F) Zoom-in views of Hel1-RNA (green), Hel2-RNA (blue) and Hel2i-RNA (cyan) interfaces. See also Figure S1, S13, S14, S15, S16, S17, Table S1, S2 and S3

Previous kinetic studies have shown that RIG-I is clamped tightly, or “throttled”, at dsRNA termini bearing a 5’-triphosphate, but not at dsRNA termini terminated by 5’-OH (Devarkar *et al.*, 2018). In the latter case, RIG-I is free to disassociate entirely or to undergo translocation to inactive internal sites along the duplex (Devarkar *et al.*, 2018), while in the former case, this process was observed to be inefficient. Here, we’ve shown that the triphosphate-clamped conformation (Vu) is partially unfolded and does not have an intact ATP binding site. To examine whether this lack of a folded ATP binding site helps to freeze RIG-I at dsRNA termini, we needed to obtain a cryo-EM structure of full-length RIG-I in the presence of ATP, in an environment where it was free to move around on the RNA lattice. To this end, we designed a 30 base-pair RNA duplex containing a hairpin loop at one end and a 5’-triphosphate at the opposite, blunt terminus (p3SLR30, Fig S1). It is similar to the p3SLR14 used in other studies (Linehan *et al.*, 2018), but it is 16 base-pairs longer (see Methods), and it readily stimulates RIG-I activation (Fig. 5A and (Kohlway *et al.*, 2013)). SLR30 is capable of binding RIG-I at only one end, and it is designed to reveal any RIG-I complexes have migrated into the middle of the duplex upon addition of ATP.

#### End-bound complexes captured in the presence of ATP

To visualize the behavior of RIG-I in this alternative context, the purified p3SLR30:RIG-I complexes (Fig S13A) were incubated with ATP and Mg^2+^ before cryo-grid preparation. A second batch of purified complexes were frozen in the absence of ATP and Mg^2+^. In this way, we hoped to visualize RIG-I bound to an elongated, closed duplex and to determine if ATP induces changes in the conformation of the complexes. For samples of RIG-I bound to p3SLR30 absence of ATP, all particles contained RIG-I anchored exclusively at the 5’-terminus of p3SLR30 (Fig S13B), as we have already observed for the triphosphorylated or diphosphorylated duplexes (Fig S2C and S3C).

For samples incubated in the presence of ATP, we obtained two different types of complexes. One class of complexes (~41% of final particles) contain RIG-I bound at the triphosphorylated terminus (Fig 5B and S14, Table S2), where it adopts the conventional Vu conformation, as described previously (Fig S15A). Importantly, despite the high concentration of ATP added to this sample, density for ATP was not observed in the map for this complex (Fig 5C), consistent with the fact that Hel2 is unfolded and therefore lacks an intact ATP binding site. Unable to bind ATP and thereby stimulate RNA release, RIG-I protein is firmly stuck at the duplex terminus. However, a second population of complexes (~59% of final particles) contained a conformation of RIG-I that is entirely novel, having bound ADP, and transformed into a conformation that has migrated into the center of the RNA duplex (Fig 5B, 5D and S15A). From this second population of complexes, we determined the first single particle cryo-EM structure of a full-length RIG-I molecule bound to an internal site on an RNA (3.2 Å, Fig S14), which provides new insights into the dynamical behavior of the RIG-I receptor (*vide infra*).

Although the Vu conformation observed in the context of p3SLR30 does not display clear ADP density within the RIG-I active-site (Fig 5C), we wondered whether this might be simply due to weak ADP binding and that the Vu conformation might in fact be capable of binding nucleotide that is more similar to a nucleotide triphosphate. To address this possibility, we solved the p3SLR30:RIG-I complex structure again, but this time we added the ATP mimic AMPPNP to the complex (Fig S16). However, AMPPNP density was not observed within the active-site of RIG-I, providing further evidence that the Vu conformation cannot bind ATP (Fig 5C).

Given that SLR30 duplexes contain a substantial “runway” for the movement of RIG-I away from the blunt duplex terminus, we wondered whether RIG-I might have a greater propensity to migrate into the center of the duplex when it starts out in the HF conformation, complexed with “host-like” RNA duplexes, such as those that terminate in a 5’-OH rather than a 5’-triphosphate. Indeed, this might assist in proof-reading by providing a second pathway for clearing RIG-I molecules from the duplex terminus. To address this question, we created a new RNA called OHSLR30, which contains a “host-like” terminus adjacent to a long duplex (Fig S1), and we solved a series of structures with RIG-I in the absence and presence of ATP. When OHSLR30 is combined with RIG-I in the absence of ATP, all RIG-I molecules are bound at the duplex terminus, forming an HF conformation identical to that observed previously for the monophosphorylated and hydroxylated duplexes (compare Fig S13B with Fig. S4C and S5C). However, when purified OHSLR30:RIG-I complexes are incubated with ATP and Mg^2+^ before cryo-grid preparation, we observe two populations of molecules: A minority population (24%) that remains bound at the duplex terminus, while a majority population (76%) moves to the center of the duplex, supporting the notion that the HF conformer is a dynamically active state that readily binds ATP and assists in proof-reading (Fig 5B and S17). Consistent with this, the end-bound HF species contains clear density for ADP within the active-site (Fig 5C and S15B).

These structural studies were then complemented by mutational functional analysis. For example, if ATP binding to the HF conformation promotes RIG-I dissociation from the dsRNA termini, thereby facilitating proof-reading and reducing the probability of RIG-I signaling, then ATP-deficient mutants should cause RIG-I to bind and signal on “host-like” dsRNA, such as OHdsRNA. It is well established that the C268F mutant is defective in ATP binding, while the C268F and E373A/Q mutants are unable to hydrolyze ATP (Devarkar *et al.*, 2018; Lassig et al., 2018). We therefore selected these mutants for testing with the IFN reporter assay, stimulating with both p3SLR30 and OHSLR30. Indeed, WT RIG-I could only be stimulated by the “viral” p3SLR30, while the C268F, E373A and E373Q mutants were equally well-stimulated by either viral p3SLR30 or host OHSLR30 (Fig 5A). Therefore, the abolition of functional ATP binding leads to abnormally constitutive signaling, even in the absence of viral dsRNA.

#### Characteristic features of translocated, internally-bound RIG-I

The internally-bound RIG-I complexes obtained in the studies described above provided unexpected insights into conformational variability of the protein and its consequences for RNA recognition (Fig S14 and S17, Table S2). RIG-I is known to bind internal duplex sites very weakly (Rawling *et al.*, 2015), and to dissociate from them with a fast off-rate (Devarkar *et al.*, 2018). But due to the high macromolecular concentrations needed for grid preparation (the same approximate concentration as the K_d_ for internally-bound RIG-I), we were able to capture and visualize these labile complexes. As described above, these structures are strikingly different from RIG-I end-bound structures previously determined. Regardless of the different RNA duplexes (p3SLR30 and OHSLR30) used here, structures of the internally-bound complex are identical (RMSD: 0.4 Å, Fig S15). In the internally-bound conformation, Hel1, Hel2, Hel2i and the CTD are wrapped around dsRNA, where they form polar contacts with both strands of the RNA backbone (Fig 5B, 5E and 5F). These attributes of the internally-bound complex, combined with the fact that OHSLR30 does not promote signaling, provide additional evidence for a lack of signaling from internal dsRNA sites (Fig 5A) and they suggest that ATP-dependent translocation might instead serve as a valuable proof-reading mechanism for RIG-I.

**Figure 6.**
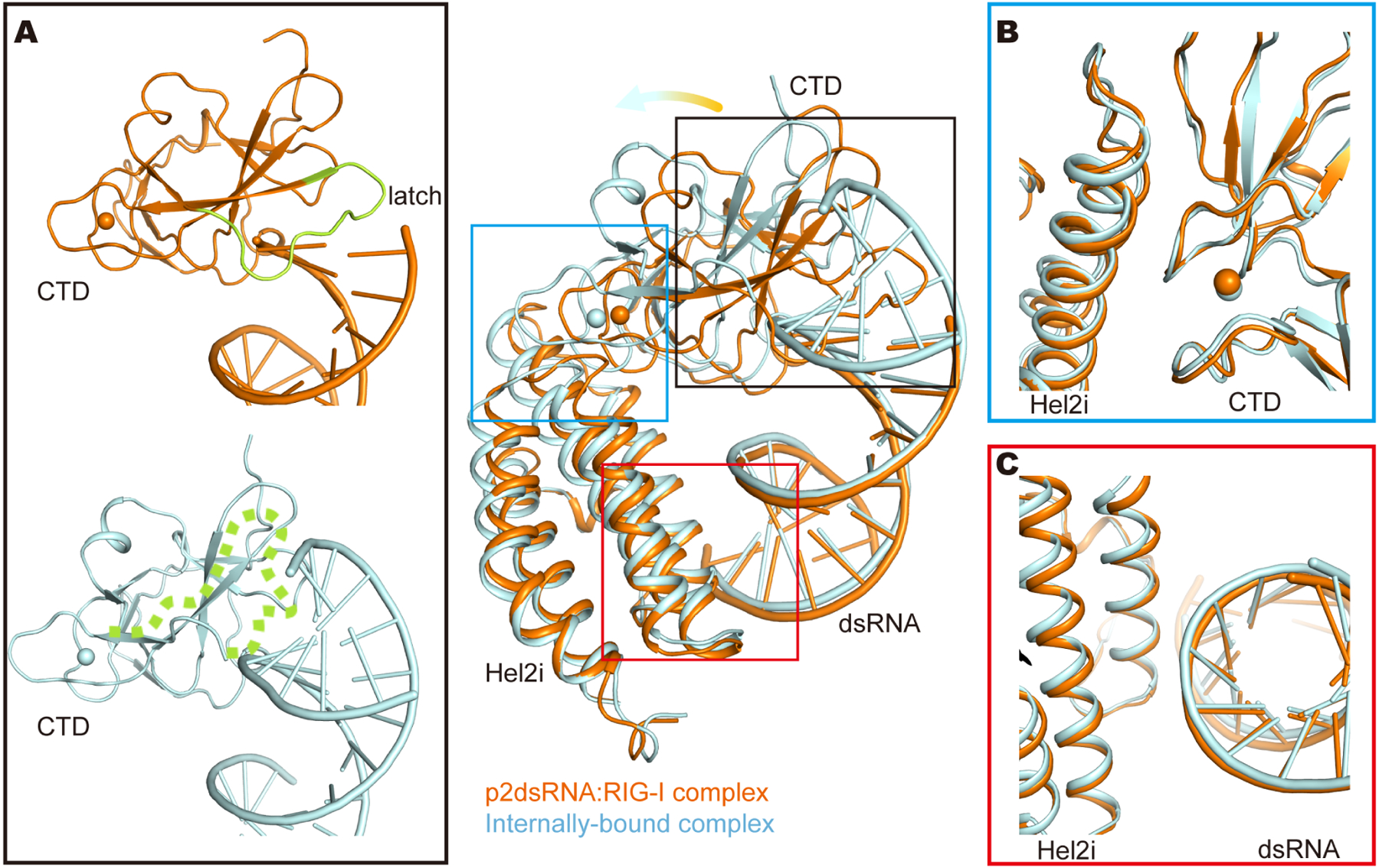
Structural basis for the weak binding between RIG-I and RNA stems. Overlay of Helicase core (Hel1, Hel2 and Hel2i; Hel1 and Hel2 are omitted in this figure) between p2dsRNA:RIG-I (orange) and the internally-bound RIG-I complexes (palecyan). (A). Different CTD conformations in p2dsRNA:RIG-I and the internally-bound complexes. The “latch” in CTD is highlighted in lime, and the disordered “latch” in the internally-bound complex is rendered as dotted line. (B). Unchanged Hel2i-CTD interface in the p2dsRNA:RIG-I and the internally-bound complexes. Overlay of CTD between p2dsRNA:RIG-I (orange) and the internally-bound RIG-I complexes (palecyan). (C). Hel2i-RNA interface in the p2dsRNA:RIG-I and the internally-bound complex. See also Figure S18

The most distinctive feature of the internally-bound complex is that the RIG-I CTD and Hel2i adopt different relative orientations compared to previous complexes. An overlay of Hel1, Hel2 and Hel2i (the main helicase domains) between internally-bound and the p2dsRNA:RIG-I complex structures reveals that the CTD is tilted away from the helicase core to avoid clashes with the contiguous RNA duplex. Lacking the typical CTD-terminus interactions, there is disorder within the CTD region that contains the phenylalanine that normally stacks on the duplex terminus (also known as “the latch”, Fig 5B and 6A). An overall reduction in CTD contact with the RNA is evident from the decrease in buried surface area (from 1586 Å^2^ to 1311 Å^2^) and reduction in intermolecular contacts (from 187 to 135), although a new set of CTD:RNA interactions are established (Fig S18A). Surprisingly, an examination of CTD positioning relative to Hel2i reveals that the tilted CTD still maintains a similar set of interactions with Hel2i as observed in the p2dsRNA:RIG-I structure (Fig 6B). Because the CTD remains effectively attached to Hel2i, the pronounced tilt in the CTD serves to pry Hel2i away from RNA, reducing the RNA-protein interface. This is evident from the dramatic decrease in buried surface area and intermolecular contacts (737 Å^2^ versus 1251 Å^2^; total contacts: 77 versus 128; Fig 6C and Table S3). Given that the CTD is the primary contributor to RIG-I-RNA binding affinity (Vela et al., 2012; Wang et al., 2010), the loss of interactions between the CTD and the blunt dsRNA terminus and the reduction in Hel2i-RNA interactions is expected to dramatically weaken RIG-I binding to internal RNA stems (see Supplementary information and Fig S18B and S18C), as observed in previous studies, which also report rapid dissociation of internal complexes (Rawling *et al.*, 2015). Indeed, direct studies of RIG-I binding to internal duplexes has shown that these complexes are very weak (4–5 orders of magnitude weaker than binding to the terminus of RNA duplexes (Devarkar *et al.*, 2018; Rawling *et al.*, 2015; Ren *et al.*, 2019; Vela *et al.*, 2012).

The design of the SLR30 complexes, together with the relative positioning of RIG-I in the presence and absence of ATP, provide important information on RIG-I loading and translocation directionality. Specifically, SLR30 is blocked at one end by a loop, so there is only one terminal position available for RIG-I loading. When RIG-I is combined with SLR30, it initially binds at this single triphosphorylated terminus, and it isn’t until ATP is added that the protein can move to an alternate internal position. This means that, as described previously in time-resolved kinetic experiments (Devarkar *et al.*, 2018), RIG-I loads at an RNA terminus and then slowly moves away from that position upon addition of ATP. It does not bind at an internal site and then scan for the terminal triphosphate.

The results with SLR30 also indicate that RIG-I is in equilibrium between the Vu and HF conformations at any given time, and that the type of RNA bound will affect this equilibrium (Movie S1). Specifically, we show that RIG-I bound to a diphosphorylated RNA duplex is predominantly in the Vu conformation, which is reinforced by strong interactions with α- and β-phosphates. However, even RIG-I in these complexes is apparently capable of transiently sampling HF, as an intact ATP binding site would have been required for RIG-I to bind ATP, hydrolyze it and move into the duplex center. Thus, in the presence of ATP, even RIG-I that originates at the high-affinity triphosphorylated terminus can sometimes detach from it and move into the low-affinity center of the RNA duplex. This indicates that there is a second way that ATP continually challenges RIG-I bound to RNA molecules, testing for high affinity binding. ATP binding not only stimulates the dissociation from RNA termini, it also facilitates directional motion to internal sites, from which dissociation is exceptionally rapid. In this way, ATP facilitates proof-reading by multiple mechanisms, ensuring that signaling is only possible when RIG-I is clamped at those few viral PAMPs that bind with exceptionally high affinity.

## DISCUSSION

### **Two receptors in one**.

RIG-I selectively differentiates viral RNAs from host RNAs by adopting two different conformations, each of which is specific for a different type of RNA (host or viral). In this way, RIG-I behaves like two proteins in one: Each of the structural isoforms plays critical role in the function of RIG-I. One isoform (which we call Vu) is a partially unfolded state of the protein that forms high affinity interactions with viral RNA PAMPs, exemplified by p2dsRNA and p3dsRNA, resulting in potent induction of interferon signaling. The other isoform (which we call HF) is a tightly folded protein that sensitively detects RNAs that are abundant in the host, exemplified by p1dsRNA and OHdsRNA. It makes few contacts with RNA ligands and contains a dynamic motor domain that facilitates rapid recycling from host RNA binding sites, thereby providing an accurate proof-reading mechanism and minimizing signaling from host dsRNAs.

### **Protein folding transitions are linked to RNA ligand identification**.

It is intriguing that RIG-I uses a protein folding transition to differentiate between host and viral RNAs and to amplify the signal relay that is mediated by the CARDs. When RIG-I encounters pathogen RNA, the CTD clamps onto the terminal diphosphate, which blocks access of Heltnt to the CTD recognition pocket and causes unfolding of Hel2 (Fig 3A). The cryo-EM structures of RIG-I complexed with p2dsRNA and p3dsRNA are consistent with HDX-MS data showing that Heltnt remains dynamic upon RIG-I binding viral dsRNA (Zheng et al., 2018). This partially unfolded form of RIG-I is then free to form a network of stable interactions with the p2dsRNA terminus and the adjacent duplex, resulting in a long-lived complex that maintains the CARDs extended into solution, thereby facilitating subsequent stages of the signaling cascade. By contrast, when RIG-I encounters host RNAs the empty CTD recognition pocket is plugged by Heltnt, which binds the same amino acids as the dsRNA diphosphate (Fig 2C and 2E). With Heltnt inserted inside the CTD, Hel2 adopts a stable RecA fold and a solid hydrophobic core, resulting in a conformational state with active motor activity, but weak affinity for RNA duplexes. Rapid off-rate from these host sites is further enhanced by active cycles of ATP binding and hydrolysis that further accelerate RNA dissociation and, in some cases, cause RIG-I to move away from the terminus to even lower affinity sites on the duplex interior (Devarkar *et al.*, 2018; Rawling *et al.*, 2015). In this way, Heltnt acts as a “kill switch” that prevents formation of a long-lived signaling complex and minimizes signaling from low-affinity host RNAs.

### **An internally-bound RIG-I complex reveals a physical basis for translocation**.

Arguably the most novel structure obtained in this study is that of RIG-I bound to an internal duplex site. Once it dislodges from the duplex terminus, the CTD changes position, tilting away from the rest of the protein and peeling away backbone contacts between RNA and the helicase domains. This results in a loose, dynamic complex consistent with measured weak binding in biochemical experiments (Devarkar *et al.*, 2018; Rawling *et al.*, 2015). The pathway for the formation of this complex, and its structural features, reveal much about RIG-I behavior. Specifically, this structure establishes that, as observed in transient kinetics and single molecule experiments (Myong et al., 2009; Yao et al., 2015), RIG-I is capable of ATP-dependent translocation from a duplex terminus to the interior of a dsRNA molecule.

Given the weak binding of RIG-I to internal sites, and the inability of end-blocked duplex stems to induce signaling (Devarkar *et al.*, 2018; Rawling *et al.*, 2015), it is unlikely that RIG-I translocation contributes positively and directly to signaling, unless internally-bound complexes such as those observed here can be stabilized under specific sets of conditions that have not yet been characterized. Consistent with this, the most potent RNA inducers of RIG-I signaling are short diphosphorylated dsRNAs on which RIG-I cannot translocate at all (Linehan *et al.*, 2018; Luo *et al.*, 2011; Rawling *et al.*, 2015). These complexes may be so active because they are effectively trapped in the activated Vu conformation.

### A unified model for RIG-I signaling applicable to healthy and disease states

**Figure 7.**
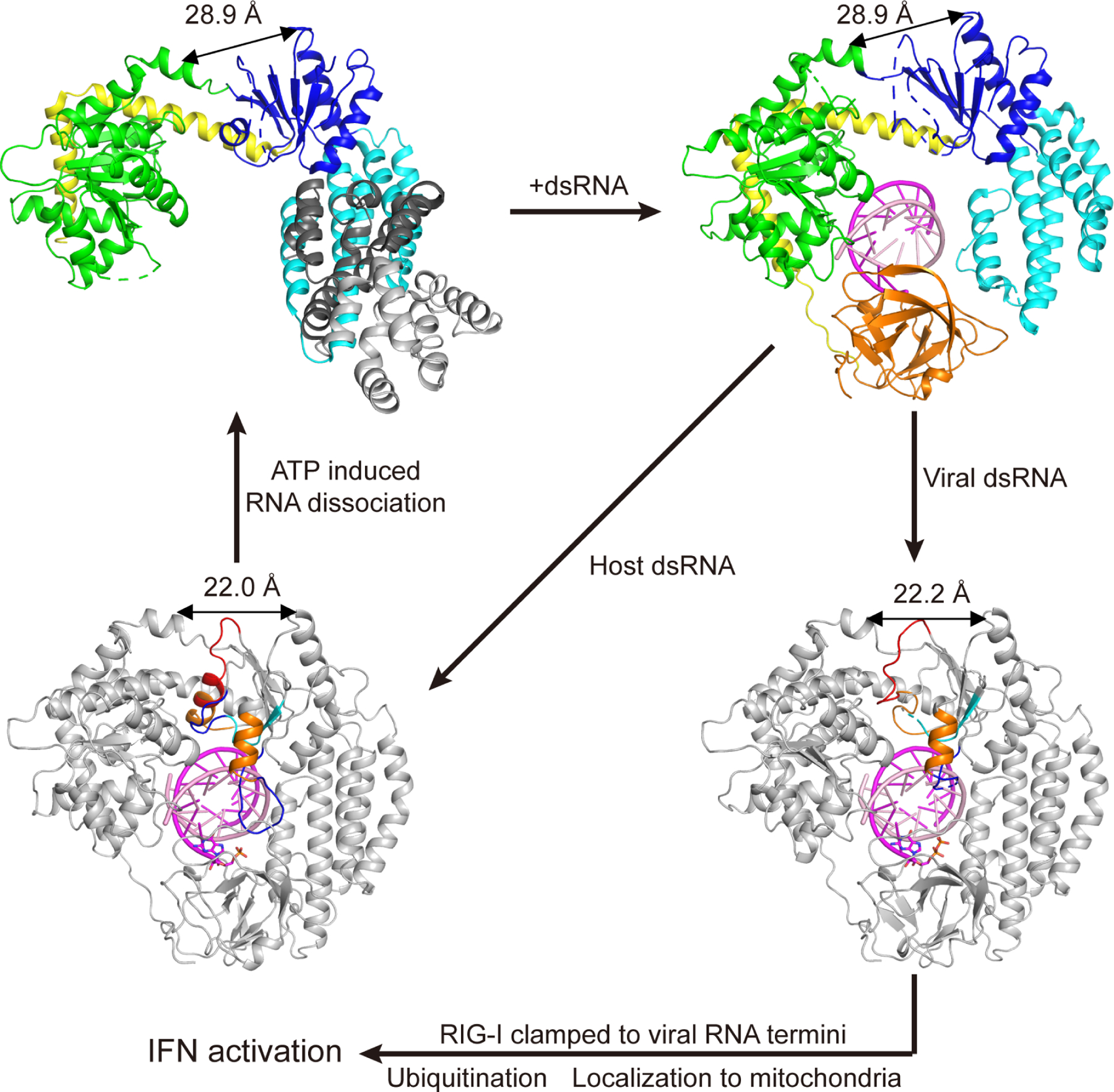
Structure-based model of RIG-I signaling Upon bound to RNA duplexes, RIG-I transforms from the apo state (PDB ID: 4a2w) to semi-closed (PDB ID: 4bpb) and then the closed state. Viral RNAs remain clamped to RIG-I for a long time, thereby preventing formation of the autoinhibited apo state of the protein, while host RNAs are rapidly released. The longer RIG-I remains bound to a given dsRNA, the longer the CARDs will remain exposed in solution, where they encounter the cofactors and binding partners necessary for completing a circuit of signaling. Post-translational modifications, such as ubiquitination, or the binding of cofactor proteins such as Riplet, are likely to trap the CARDS-out state and/or prevent reassociation of the CARDs, thereby forcing the signaling cascade in a forward direction. See also Movie S1

RIG-I does not selectively bind viral dsRNAs. It binds and samples all dsRNAs that it encounters, releasing its CARDs, and using the dynamic flexibility of Hel2 domain folding to test whether the RNA belongs to host or pathogen (Fig 7 and S19). Successful signaling is therefore likely to ultimately depend on the residence time of RIG-I on a particular RNA ligand. Viral RNAs remain stuck on RIG-I for a long time, and they repress formation of the autoinhibited apo state of the protein, while host RNAs are rapidly released (Fig 7). The longer RIG-I remains bound to a given dsRNA, the longer the CARDs will remain exposed in solution, where they encounter the cofactors and binding partners necessary for completing a circuit of signaling (Fig 7). If CARD release is fleeting and reversible (Dickey *et al.*, 2019), then only a small population of complexes have the potential form the interactions needed for signaling. These appear to have a negligible impact on interferon levels unless the concentration of host dsRNAs becomes extraordinarily high or mutations in RIG-I result in long-lived complexes with host dsRNA (Lassig *et al.*, 2018; Lassig et al., 2015; Louber et al., 2015; Manivannan et al., 2020; Yang et al., 2014). The structural basis for these kinetic behaviors, which have long been observed, is now clear from this comprehensive set of solution-state dsRNA:RIG-I complexes.

Here we show that viral RNAs terminated by a 5’-diphosphate or triphosphate form tight RIG-I complexes (Vu conformers) that are stabilized through two different strategies: (A). In the Vu conformation, a massive number of interactions are formed between RIG-I and viral dsRNA termini, essentially fusing the CTD and atoms of the diphosphate terminus, consistent with the high binding affinity (Ren *et al.*, 2019; Vela *et al.*, 2012). By effectively fusing RIG-I to the blunt diphosphate terminus of viral dsRNAs, the CARD domains are prevented from rebinding to Hel2i. (B). The unfolding of Hel2 prevents binding and hydrolysis of ATP, which further slows the process of RNA release and inhibits ATP-catalyzed translocation to low affinity internal duplex sites.

Structures of RIG-I in complex with p1dsRNA and OHdsRNA (HF conformers) explain the physical mechanism of active proof-reading, revealing why host ligands are not conducive to strong signaling. HF complexes form a reduced network of interactions with dsRNA, which is consistent with their fast off-rates (Devarkar *et al.*, 2018; Rawling *et al.*, 2015) and low binding affinity (Ren *et al.*, 2019; Vela *et al.*, 2012). At the same time, with Heltnt engaged in the CTD or along the dsRNA backbone, the Hel2 domain remains well-folded in these complexes, enabling them to engage with ATP and stimulating active RNA dissociation or translocation away from the high-affinity terminus. While the CARDs are accessible in HF complexes, their exposure to solvent is fleeting, so signaling is suppressed. However if host RNA becomes high in concentration, thereby driving accumulation of dsRNA:RIG-I complexes, high levels of signaling will be maintained, and this is likely the cause of gain-of-function diseases and interferonopathies associated with RIG-I (Lassig *et al.*, 2018; Lassig *et al.*, 2015; Louber *et al.*, 2015; Manivannan *et al.*, 2020; Yang *et al.*, 2014).

### Limitations of the study

Adding to underlying kinetic equilibria that control RIG-I signaling, post-translational modifications, such as ubiquitination, or the binding of cofactor proteins such as Riplet, are likely to amplify the effects reported here by trapping the CARDs-out state and/or preventing reassociation of the CARDs, thereby forcing the signaling cascade in a forward direction (Cite first Riplet paper). These processes have their own limiting timescales, which in turn could contribute to the regulation signaling. A second limitation is that this study was designed only to capture structural details of RIG-I:RNA recognition specificity, and it was not designed to facilitate observation of the CARDs, nor their interactions with ubiquitin ligases or the MAVS adapter protein. Understanding of these larger complexes and their dynamic interplay awaits future structure-function studies with an expanded scope. A third limitation is that, while this study provides a first glimpse of actively-translocated RIG-I molecules along the dsRNA duplex, direct kinetic studies of this process, particularly involving disease mutants known to be defective in proof-reading, will be needed to understand whether RIG-I movement along dsRNA serves a specific functional role within the innate immune system.

## STAR METHODS

### LEAD CONTACT AND RESOURCE AVAILABILITY

#### Lead Contact

All requests for resources and reagents should be directed to and will be fulfilled by the Lead Contact, Anna Marie Pyle (anna.pyle@yale.edu).

#### Materials Availability

All materials will be available from the Lead Contact with a completed Materials Transfer Agreement.

#### Data and Code Availability

- Atomic coordinates and cryo-EM maps are deposited in EMDB and PDB as follows: p3dsRNA:RIG-I complex (EMDB: EMD-26022, PDB: 7TNX), p2dsRNA:RIG-I complex (EMDB: EMD-26023, PDB: 7TNY), p1dsRNA:RIG-I complex (EMDB: EMD-26024, PDB: 7TNZ), OHdsRNA:RIG-I complex (EMDB: EMD-26025, PDB: 7TO0), p3SLR30 end bound complex (EMDB: EMD-26026, PDB: 7TO1), p3SLR30 internally-bound complex (EMDB: EMD-26027, PDB: 7TO2), OHSLR30 end bound complex (EMDB: EMD-27744, PDB: 8DVS), OHSLR30 internally-bound complex (EMDB: EMD-27745, PDB: 8DVu) and p3SLR30:RIG-I (+AMPPNP) complex (EMDB: EMD-27743, PDB: 8DVR).
- This paper does no report original code.
- Any additional information required to reanalyze the data reported in this paper is available from the lead contact upon request.

### EXPERIMENTAL MODELS AND SUBJECT DETAILS

#### Cell lines

HEK293T cells were grown in high glucose Dulbecco’s Modified Eagle Medium (DMEM) supplemented with 10% heat inactivated fetal bovine serum (HI-FBS) at 37 °C with 5% CO2.

### METHOD DETAILS

#### Cloning, Expression, and Purification of RIG-I

The human RIG-I protein was expressed and purified as described previously (Ren *et al.*, 2019). In brief, RIG-I was fused to an N-terminal 6xHis tag and a SUMO tag, followed by ULP1 digestion site in ChampionTM pET SUMO vector (ThermoFisher Scientific). This construct was overexpressed in E.coli Rosetta™ 2(DE3) Singles™ Competent Cells (Millipore Sigma). RIG-I expression was induced by IPTG (0.5 mM) when OD600 reached 0.6 and proceeded for 20–24 hours at 16 °C. The pellets were lysed in buffer (25 mM HEPES, pH 8.0, 300 mM NaCl, 10% Glycerol, 5 mM BME) supplemented with EDTA-free Protease Inhibitor Cocktail (Sigma), followed by nickel affinity chromatography using Ni-NTA Superflow beads (Qiagen). RIG-I was treated by ULP1 to remove the SUMO tag, followed by cation exchange and size exclusion chromatography, using a HiTrap Heparin HP column (GE Healthcare) and then a Superdex 200 Increase 10/300 GL column (GE Healthcare). RIG-I was pooled in a storage buffer (25 mM HEPES, pH 7.4, 200 mM NaCl, 5% Glycerol, 5 mM BME) for use in further experiments. RIG-I used in cryo-EM studies was pooled in buffer without glycerol.

#### RNA Preparation

RNA oligonucleotides (p3dsRNAa, p3dsRNAb; p2dsRNA; p1dsRNA; OHdsRNA) were synthesized in-house using an automated MerMade synthesizer (BioAutomation, Irving, TX, United States) with phosphoramidites from Glen Research using standard phosphoramidite chemistry. Oligonucleotides were deprotected and gel purified as previously described (Wincott et al., 1995), and evaluated for purity by mass spectrometry (Novatia). Briefly, base deprotection was performed in a 1:1 mixture of 30% ammonium hydroxide (JT Baker) and 40% methylamine (Sigma) at 65 °C for 10 min. The supernatant was cooled on ice and evaporated to dryness in a new vial. With the addition of 500 µl absolute ethanol, the solution was evaporated to dryness. In order to deprotect the 2’-OH groups, the pellet was incubated with 500 µl of 1 M solution of tetrabutylammonium fluoride (TBAF) in Tetrahydrofuran (Sigma) at RT for 36 h. Then with addition of 500 µl 2 M sodium acetate (pH 6.0), the solution was evaporated to about 500 µl, extracted with 3 × 800 µl ethyl acetate, following ethanol precipitation. The RNA oligonucleotides were purified using 16% urea denaturing polyacrylamide gel and its purity was assessed by mass spectrometry (Novatia). The stem loop RNA (p3SLR30) was in-vitro transcribed, purified and evaluated as described previously (Dickey *et al.*, 2019; Rawling *et al.*, 2015). In brief, the p3SLR30 was in-vitro transcribed using T7 RNA polymerase with synthetic dsDNA template (Integrated DNA Technologies) containing 2’-OMe modifications on the first two nucleotides of the 5’ terminus of the negative-sense strand. A 100 µl transcription solution contains 1 µg of annealed template, 40 mM Tris-HCl (pH 8.0), 22 mM MgCl2, 10 mM DTT, 2 mM spermidine, 0.01% Triton X-100, 5 mM of each NTPs, 40 U of RNaseOUT™ Recombinant Ribonuclease Inhibitor (Thermo Fisher), and 5 µl of T7 RNA polymerase. With 12-hour incubation at 37 °C, the transcribed p3SLR30 was purified by gel extraction from 12%-20% urea denaturing polyacrylamide gel and its purity was assessed by mass spectrometry (Novatia). With NEB CIP treatment, the triphosphate group of p3SLR30 was removed, resulting in a stem loop RNA containing 5’-OH blunt terminus (OHSLR30), which was evaluated by MS. RNA duplexes (p3dsRNA, p2dsRNA, p1dsRNA, OHdsRNA) and stem loop RNA (p3SLR30, OHSLR30) were annealed and stabilized before use in experiments. Specifically, two-stranded RNA duplexes (260 µM) were annealed by rapidly heating to 99 °C and slowly cooling over 1 hour to 4 °C in annealing buffer (200 mM NaCl) on a Thermocycler. The p3SLR30 and OHSLR30 were heated to 90 °C for 2 min and then snap-cooled on ice for 30 min. Purity of annealed duplex RNAs was assessed by running samples on a 15% native polyacrylamide gel and visualized by Amersham Typhoon (GE). Sequences of the RNAs used in this study are shown in Table S1.

#### Separation of RNA:RIG-I complexes

To prepare RNA:RIG-I complexes, purified RIG-I and RNA were mixed and incubated overnight at 4°C. The RNA:RIG-I complexes were separated using a Superdex 200 Increase 10/300 GL column (GE Healthcare). The complexes were pooled in size-exclusion buffer (25 mM HEPES, pH 7.4, 200 mM NaCl, 5 mM BME) and concentrated with a 50 kD Amicon Ultra-0.5 Centrifugal Filter Unit (Millipore Sigma). The absorbance was estimated using a Nanodrop (ThermoFisher) for concentration quantification. RIG-I was mixed with p3dsRNA, p2dsRNA, p1dsRNA, OHdsRNA, p3SLR30 and OHSLR30 in an 8:1, 2:1, 1.5:1, 2:1, 1:1 and 1:1 molar ratio, and peak fractions of RNA:RIG-I complex were collected.

#### Pipeline development for Cryo-EM structure-determination:

As in earlier crystallographic studies, we initially isolated complexes and performed single-particle cryo-EM studies on RIG-I in complex with short dsRNA ligands (14 bp). While these complexes resulted in high quality data, we found that lengthening the RNA stem had several technical advantages and it improved our ability to image the particles due to the inherently high contrast of RNA duplexes. To develop a pipeline for routine analysis of RIG-I complexes on dsRNA we designed a set of 24-bp double blunt-ended RNA duplexes containing potential binding sites for RIG-I binding at each end (Fig S1). This design increased the probability of obtaining uniform bound complexes and resulted in particles that are easily identifiable from their shape, thereby speeding the process of particle-picking. Specifically, at low resolution, RIG-I is rapidly identifiable as a blob of density localized at the end of a long stick, representing the RNA duplex that projects asymmetrically from the side of the protein. Using this approach, it was possible to obtain complexes with an overall resolution of 3.5 Å (local resolution of core is around 2.5 Å). The long unbound RNA stem enhanced particle alignment accuracy and alleviated the problems of preferred orientation by using 1:2 RNA:RIG-I complexes in orientations which are sparse in 1:1 RNA:RIG-I complexes (Fig S2A–S2G). The double blunt-ended p2dsRNA, p1dsRNA and OHdsRNA for cryo-EM studies were synthesized using scaffolds similar to the p3dsRNA described above (Fig S1; Table S1).

#### Cryo-EM sample preparation

To prepare for freezing cryo-EM grids, the fresh samples were obtained and concentrated. The p3dsRNA:RIG-I, p2dsRNA:RIG-I, p1dsRNA:RIG-I, OHdsRNA:RIG-I, p3SLR30:RIG-I and OHSLR30:RIG-I complexes were concentrated to 0.3, 1.5, 0.4, 1.5, 1.5 and 1.5 mg/ml. The Quantifoil holey carbon R1.2/1.3 300 mesh Cu grids (Ted Pella) were glow discharged using the PELCO easiGlow^™^ Glow Discharge Cleaning System (Ted Pella) for 35 s at 25 mA. With purified RNA:RIG-I complexes (3 µl) applied onto the grids, the grids were blotted and plunged to the liquid ethane for flash freezing using a Vitrobot Mark IV (ThermoFisher). The blotting conditions for all grids were similar, under conditions of 22 °C and 100% humidity with force -4. Blotting times were 4 s, 5 s, 4 s, 4 s, 3 s and 3 s for p3dsRNA:RIG-I, p2dsRNA:RIG-I, p1dsRNA:RIG-I, OHdsRNA:RIG-I, p3SLR30:RIG-I and OHSLR30:RIG-I complexes, respectively. In the case of p3SLR30:RIG-I complex with ATP in solution, OHSLR30:RIG-I complex with ATP in solution and p3SLR30:RIG-I with AMPPNP in solution, 3, 20, 20 µM (0.3, 2.3, 2.3 mg/ml) of complex was incubated with 2.5 mM ATP/AMPPNP and 5 mM MgCl2 on ice for 30 min before being applied to the grids, and the blotting conditions are the same as p3SLR30:RIG-I complex in the absence of ATP described above. The frozen grids were all transferred and kept in liquid nitrogen prior to use in data collection.

#### Cryo-EM data acquisition

Cryo-EM data were acquired at Yale Cryo-EM facilities on a Titan Krios transmission electron microscope (ThermoFisher) operating at 300 keV and equipped with a Gatan K2/K3 Summit direct electron detector using SerialEM software at super-resolution mode. For the p3dsRNA:RIG-I, p2dsRNA:RIG-I, p1dsRNA:RIG-I and OHdsRNA:RIG-I complexes, 3480, 2586, 2460 and 2838 micrographs were collected at a nominal magnification of 130,000 ×, 81,000 ×, 81,000 × and 81,000 ×, corresponding to calibrated pixel size of 1.05, 1.068, 1.068 and 1.068 Å/pix, with a defocus range of -1.2 to -2.7, -1.2 to -3.0, -1.2 to -3.0 and -1.2 to -3.0 µm. Each micrograph contains 34, 38, 38 and 38 frames and was collected with an exposure rate of 7.9, 17.7, 17.7 and 17.7 e^-^/pix/s and total electron exposure of 61, 59, 59 and 59 e^-^/Å^2^. For the p3SLR30:RIG-I complex with ATP or AMPPNP addition, 3417 and 2744 micrographs were collected at a nominal magnification of 130,000 × and 81,000 ×, corresponding to calibrated pixel size of 1.05 and 1.068 Å/pix, with a defocus range of -1.2 to -2.7 and -1.2 to -3.0 µm. Each micrograph contains 40, 38 frames and was collected with an exposure rate of 9.4, 18 e^-^/pix/s and total electron exposure of 55, 60 e^-^/Å^2^. For the p3SLR30:RIG-I and OHSLR30:RIG-I complexes without ATP addition, and OHSLR30:RIG-I complex with ATP addition, the data were acquired at Yale Cryo-EM facilities on a Glacios transmission electron microscope (ThermoFisher) operating at 200 keV and equipped with a Gatan K2 Summit direct electron detector using SerialEM software at super-resolution mode. 2773, 2264 and 3663 micrographs were collected at a nominal magnification of 36,000×, corresponding to calibrated pixel size of 1.143, 1.149 and 1.149 Å/pix, with a defocus range of -1.2 to -2.5, -1.0 to -2.0 and -0.8 to -2.0 µm. Each micrograph contains 40, 53 and 53 frames and was collected with an exposure rate of 9.3, 6.3 and 6.3 e^-^/pix/s and total electron exposure of 57, 50 and 50 e^-^/Å^2^. The statistics of data acquisition are summarized in Table S2.

#### Cryo-EM data processing

All datasets were processed through Relion (Zivanov *et al.*, 2018). The micrographs were dose-weighted and beam induced motion corrected through MotionCor2 (Zheng *et al.*, 2017). The non-dose-weighted and motion corrected micrographs were used to estimate the CTF parameters using CTFFIND4 (Rohou and Grigorieff, 2015). Micrographs were selected based on the Total motion and image resolution, and further selected by manual screening.

For the p3dsRNA:RIG-I complex, 1,996,263 particles were initially picked from 3167 selected micrographs using the 3D reference of p3dsRNA:RIG-I complex reconstructed from a cryo-EM dataset collected on a 200 KeV Glacios with Gatan K2 Summit direct electron detector located at Yale Cryo-EM facility. The particles were subjected to several rounds of 2D classification. Particles from all 2D classes containing a 1:1 RNA:RIG-I complex and from 2D classes showing 2:1 RNA:RIG-I complex in top and bottom views were selected. The selected particles were then used to generate the initial model through Relion, and subjected to several rounds of 3D classification. To improve the resolution, the unbound end of p3dsRNA in the 3D map generated above was erased through Chimera (Pettersen *et al.*, 2004), and a soft mask was applied to re-implement the 3D classification with selected particles from the 2D classes. The best 3D class contained 613,519 particles, which were then subjected to 3D refinement, Ctfrefine and Byaesian polishing, followed by a final 3D refinement (Zivanov *et al.*, 2018; Zivanov et al., 2019). The postprocessing yielded a map at global resolution of 3.5 Å, sharpened with B-factor of - 150.000 Å^2^, according to the FSC = 0.143 criterion (Rosenthal and Henderson, 2003). The strategy and statistics of data processing are summarized in Table S2 and Figure S2.

For the p2dsRNA:RIG-I complex, 3,354,308 particles were initially picked from 2370 selected micrographs using p3dsRNA:RIG-I complex as the 3D reference. The particles were rescaled, and subjected to several rounds of 2D classification, 3D classification as described above. The best 3D class contained 957,706 particles, which were rescaled to 1.068 Å/pix and subjected to 3D refinement, Ctfrefine and Byaesian polishing, following a final 3D refinement (Zivanov *et al.*, 2018; Zivanov *et al.*, 2019). The postprocessing yielded a map at global resolution of 3.2 Å, sharpened with B-factor of -126.40 Å^2^, according to the FSC = 0.143 criterion (Rosenthal and Henderson, 2003). The strategy and statistics of data processing are summarized in Table S2 and Figure S3.

For the p1dsRNA:RIG-I complex, 3,005,433 particles were initially picked from 2242 selected micrographs using the 3D map of p3dsRNA:RIG-I complex as the 3D reference. The particles were rescaled, and subjected to several rounds of 2D classification, 3D classification as described above. The best 3D class contained 624,117 particles, which were subjected to 3D refinement, Ctfrefine and Byaesian polishing, following a final 3D refinement (Zivanov *et al.*, 2018; Zivanov *et al.*, 2019). The postprocessing yielded a map at global resolution of 3.5 Å, sharpened with B-factor of -189.05 Å^2^, according to the FSC = 0.143 criterion (Rosenthal and Henderson, 2003). The strategy and statistics of data processing are summarized in Table S2 and Figure S4.

For the OHdsRNA:RIG-I complex, 3,357,160 particles were initially picked up from 2564 selected micrographs using the 3D map of p3dsRNA:RIG-I complex as the 3D reference. The particles were rescaled, and subjected to several rounds of 2D classification, 3D classification as described above. The best 3D class contained 435,184 particles, which were rescaled to 1.068 Å/pix and subjected to 3D refinement, Ctfrefine and Byaesian polishing, following a final 3D refinement (Zivanov *et al.*, 2018; Zivanov *et al.*, 2019). The postprocessing yielded a map at global resolution of 3.5 Å, sharpened with B-factor of -146.58 Å^2^, according to the FSC = 0.143 criterion (Rosenthal and Henderson, 2003). The strategy and statistics of data processing are summarized in Table S2 and Figure S5

For the p3SLR30:RIG-I complex with ATP addition, 2,188,358 particles were initially picked up from 3241 selected micrographs using p3dsRNA:RIG-I complex as the 3D reference through Gautomatch (developed by Kai Zhang, https://www2.mrc-lmb.cam.ac.uk/research/locally-developed-software/zhang-software/). The particles were rescaled, and subjected to several rounds of 2D classification, 3D classification as described above. An internally-bound and an end-bound complex were generated. The 3D class of internally-bound complex and end-bound complex contained 366,751 and 253,073 particles, which were subjected to 3D refinement, Ctfrefine and Byaesian polishing, following a final 3D refinement (Zivanov *et al.*, 2018; Zivanov *et al.*, 2019). The postprocessing yielded a map at global resolution of 3.2 Å and 3.7 Å, sharpened with B-factor of -82.00 Å^2^ and -140.00 Å^2^, according to the FSC = 0.143 criterion (Rosenthal and Henderson, 2003). The strategy and statistics of data processing are summarized in Table S2 and Figure S14. For the p3dsRNA:RIG-I complex with AMPPNP addition, 3,097,952 particles were initially picked from 2744 selected micrographs using p3dsRNA:RIG-I complex as the 3D reference. The particles were rescaled, and subjected to several rounds of 2D classification, 3D classification as described above. The best 3D class contained 692,833 particles, which were rescaled to 1.068 Å/pix and subjected to 3D refinement, Ctfrefine and Byaesian polishing, following a final 3D refinement (Zivanov *et al.*, 2018; Zivanov *et al.*, 2019). The postprocessing yielded a map at global resolution of 3.3 Å, sharpened with B-factor of -157.95 Å^2^, according to the FSC = 0.143 criterion (Rosenthal and Henderson, 2003). The strategy and statistics of data processing are summarized in Table S2 and Figure S16.

For the OHSLR30:RIG-I complex with ATP addition, 3,635,886 particles were initially picked up from 3599 selected micrographs using p3dsRNA:RIG-I complex as the 3D reference. The particles were rescaled, and subjected to several rounds of 2D classification, 3D classification as described above. An internally-bound and an end-bound complex were generated. The 3D class of internally-bound complex and end-bound complex contained 1,224,061 and 386,764 particles, which were subjected to 3D refinement, Ctfrefine and Byaesian polishing, following a final 3D refinement (Zivanov et al., 2018; Zivanov et al., 2019). The postprocessing yielded a map at global resolution of 3.0 Å and 3.0 Å, sharpened with B-factor of -116.23 Å^2^ and -118.35 Å^2^, according to the FSC = 0.143 criterion (Rosenthal and Henderson, 2003). The particles of internally-bound complex were further classified and yielded a map at global resolution of 2.9 Å, sharpened with B-factor of -114.44 Å^2^, according to the FSC = 0.143 criterion. The strategy and statistics of data processing are summarized in Table S2 and Figure S17.

For the p3SLR30:RIG-I and OHSLR30:RIG-I complexes without ATP addition, 2,102,250 and 1,767,040 particles were initially picked up from 2684 and 1879 selected micrographs using the 3D map of p3SLR30:RIG-I complex as the 3D reference. The particles were rescaled, and subjected to several rounds of 2D classification to reveal whether most RIG-I molecules bind to the terminus of p3SLR30 and OHSLR30.

#### Model building, refinement and validation

For the p3dsRNA:RIG-I complex, PDB ID 5F9H was used as the initial search model for building into the cryo-EM map, using molrep in the ccpem software suit (Burnley et al., 2017; Devarkar *et al.*, 2016). The p3dsRNA:RIG-I complex was used as the initial search model for the p2dsRNA:RIG-I, p1dsRNA:RIG-I, OHdsRNA:RIG-I, p3SLR30 end-bound complexes and p3SLR30:RIG-I complex with AMPPNP addition. For the p3SLR30 internally-bound complex, PDB ID 5E3H was used as the initial search model (Jiang *et al.*, 2011). For the OHSLR30 end bound and internally-bound complexes, the OHdsRNA:RIG-I and p3SLR30 internally-bound complexes were used as the initial search model, respectively. All models were built and manually adjusted in Coot (Emsley *et al.*, 2010). Then the models were refined against the cryo-EM maps using phenix.real_space_refine within phenix and refmac5 within the ccpem software suit (Afonine *et al.*, 2018; Burnley *et al.*, 2017). Models were validated using Comprehensive validation (cryo-EM) in phenix (Afonine *et al.*, 2018; Williams *et al.*, 2018). All maps and models were further validated through the PDB validation server. The statistics of model building, refinement and validation are summarized in Table S2. All the figures and movie were generated using PyMOL (http://www.pymol.org/) and Chimera (Pettersen *et al.*, 2004).

#### IFN-β induction assay

The IFN-β induction assay was implemented to test RIG-I mutants as described previously (Ren *et al.*, 2019). In brief, 500 µl of HEK293T cells at a concentration of 100,000 cells/ml in Dulbecco’s Modified Eagle Medium (DMEM, ThermoFisher) supplemented with 10% heat-inactivated Fetal Bovine Serum (HI-FBS, ThermoFisher) was seeded into each well of 24-well plate (Corning). 24 hours after the seeding, the cells of each well were transfected with 3 ng of pUNO1-RIG-I, 6 ng of pRL-TK and 150 ng of IFN-β/Firefly using the lipofectamine 2000 transfection reagent (ThermoFisher). The RIG-I expression was allowed to proceed for 24 hours, at which point the cells of each well were challenged with 1 µg of RNAs using lipo2000 reagent. 12–16 hours after the stimulation, the HEK293T cells were lysed and the IFN-β induction was measured using the Dual-Luciferase Reporter Assay System (Promega) and a Synergy Neo2 Hybrid Multi-Mode Reader (Biotek). The IFN-β induction level, the relative luminescence unit (RLU, Fluc/Rluc), is the firefly luciferase activity normalized to the renilla luciferase activity. The data were further processed with GraphPad Prism.

#### Quantification and Statistical Analysis

Statistical details of cryo-EM analyses can be found in Tables S2 and S3.

## Supplementary Material

1

2 Movie S1. RIG-I distinguishes viral from host RNA duplexes by staying in equilibrium between the Vu and HF conformations. Related to Figure 3 and 7.

## Figures and Tables

**Table:** KEY RESOURCES TABLE

REAGENT or RESOURCE	SOURCE	IDENTIFIER
Chemicals, Peptides, and Recombinant Proteins		
IPTG	AmericanBio	AB00841–00050
chloramphenicol	Sigma-Aldrich	C0378–25G
Kanamycin	Sigma-Aldrich	10106801001
Blasticidin	Invivogen	ant-bl-1
EDTA-free Protease Inhibitor Cocktail	Sigma-Aldrich	11873580001
2-Mercaptoethanol	Sigma-Aldrich	M6250
SUMO protease (ULP1)	Self-made	N/A
Adenosine 5’-triphosphate disodium salt hydrate	Sigma-Aldrich	A2383
Adenosine 5′-(β,γ-imido)triphosphate lithium salt hydrate	Sigma-Aldrich	A2647–25MG
Opti-MEM, Reduced Serum Medium	Thermo Fisher Scientific	31985070
Lipofectamine 2000 Transfection Reagent	Thermo Fisher Scientific	11668030
Bacteria		
Rosetta 2 (DE3) Singles Competent Cells	Millipore	71400–3
Recombinant DNA		
pET-SUMO-humanRIG-I and mutants	(Ren *et al.*, 2019; Vela *et al.*, 2012)	N/A
pUNO-humanRIG-I and mutants	Invivogen; (Ren *et al.*, 2019)	puno1-hrigi; N/A
pRL-TK plasmid	Promega	E2241
IFN-β/Firefly luciferase plasmid	(Ren *et al.*, 2019)	N/A
Critical Commercial Assays		
Q5 Site-Directed Mutagenesis Kit	New England Biolabs	E0554S
Dual luciferase reporter assay system	Promega	E1910
Quick CIP	NEB	M0525S
Experimental Models: Cell Lines		
Human: HEK293T	ATCC	CRL-3216
Oligonucleotides		
ssRNA oligonucleotides (see Table S1 for sequences)	Self-made	N/A
Software and Algorithms		
GraphPad Prism 7	GraphPad	SCR_002798; http://www.graphpad.com/scientificsoftware/prism
SerialEM	(Mastronarde, 2005)	SCR_017293; http://bio3d.colorado.edu/SerialEM/
MotionCor2	(Zheng et al., 2017)	SCR_016499; https://msg.ucsf.edu/em/software/motioncor2.html
CtfFinder4	(Rohou and Grigorieff, 2015)	SCR_016732; https://grigoriefflab.umassmed.edu/ctffind4
Relion 3.0	(Zivanov et al., 2018)	SCR_016274; https://www3.mrc-lmb.cam.ac.uk/relion/index.php/Download_%26_install
UCSF Chimera	(Pettersen et al., 2004)	SCR_004097; https://www.cgl.ucsf.edu/chimera/
Phenix	(Afonine et al., 2018)	SCR_014224; https://www.phenix-online.org/
Coot	(Emsley et al., 2010)	SCR_014222; https://www2.mrc-lmb.cam.ac.uk/personal/pemsley/coot/
Pymol	Schrödinger, LLC	SCR_000305; https://pymol.org/2/
MolProbity	(Williams et al., 2018)	SCR_014226; http://molprobity.biochem.duke.edu/
Deposited Data		
p3dsRNA:RIG-I complex	EMDB, PDB	EMDB: EMD-26022, PDB: 7TNX
p2dsRNA:RIG-I complex	EMDB, PDB	EMDB: EMD-26023, PDB: 7TNY
p1dsRNA:RIG-I complex	EMDB, PDB	EMDB: EMD-26024, PDB: 7TNZ
OHdsRNA:RIG-I complex	EMDB, PDB	EMDB: EMD-26025, PDB: 7TO0
p3SLR30 end bound complex	EMDB, PDB	EMDB: EMD-26026, PDB: 7TO1
p3SLR30 internally-bound complex	EMDB, PDB	EMDB: EMD-26027, PDB: 7TO2
OHSLR30 end bound complex	EMDB, PDB	EMDB: EMD-27744, PDB: 8DVS
OHSLR30 internally-bound complex	EMDB, PDB	EMDB: EMD-27745, PDB: 8DVu
p3dsRNA:RIG-I complex (+AMPPNP)	EMDB, PDB	EMDB: EMD-27743, PDB: 8DVR
Other		
Quantifoil holey carbon R1.2/1.3 300 mesh Cu grids	Ted Pella	https://www.tedpella.com/calibration_html/QUANTIFOIL_TEM_Substrates.htm
Ni-NTA Superflow beads	Qiagen	30230
HiTrap Heparin HP column	GE healthcare	GE17–0407-01
Superdex 200 Increase 10/300 GL column	GE healthcare	28990944

## Highlights

Cryo-EM structures of RIG-I complexed with different viral and host RNA ligands

RIG-I adopts two different conformations: one for viral RNA, the other for host RNA

RIG-I unfolds its motor domain in order to clamp tightly to the terminus of viral RNA

Bound to host RNA, the motor domain folds and actively facilitates rapid RNA release
