# Supplementary material for: The RIG-I receptor adopts two different conformations for distinguishing host from viral RNA ligands
Source: Mol Cell. Author manuscript; Available in PMC 2022 Nov 29. (PMC9707737; doi:10.1016/j.molcel.2022.09.029)
Supplement: 1 [file NIHMS1847035-supplement-1.pdf]

Figure S1

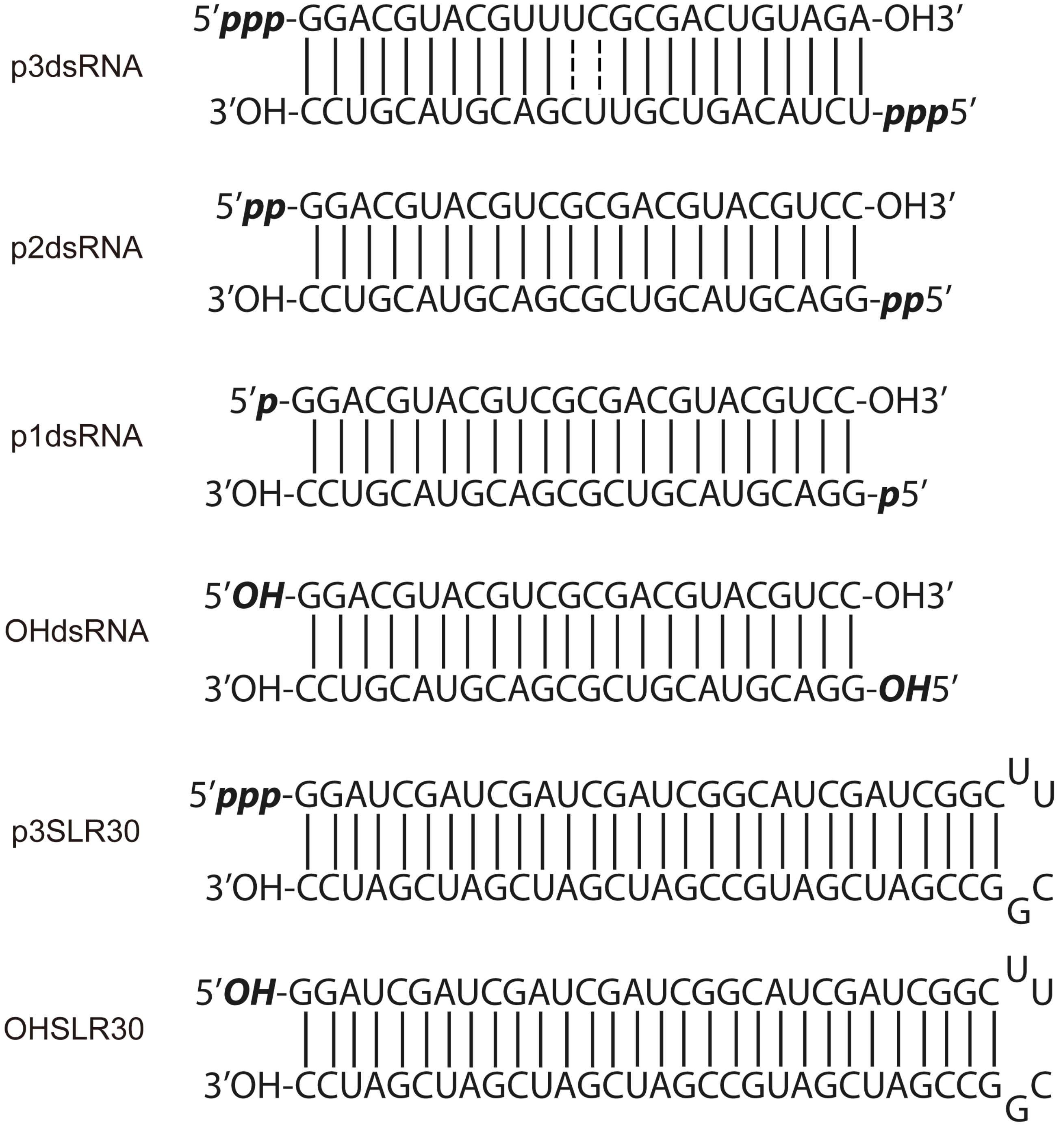
p3dsRNA
5′ppp-GGACGUACGUUUCGCGACUGUAGA-OH3′
3′OH-CCUGCAUGCAGCUUGCUGACAUCU-ppp5′
p2dsRNA
5′pp-GGACGUACGUCGCGACGUACGUCC-OH3′
3′OH-CCUGCAUGCAGCGCUGCAUGCAGG-pp5′
p1dsRNA
5′p-GGACGUACGUCGCGACGUACGUCC-OH3′
3′OH-CCUGCAUGCAGCGCUGCAUGCAGG-p5′
OHdsRNA
5′OH-GGACGUACGUCGCGACGUACGUCC-OH3′
3′OH-CCUGCAUGCAGCGCUGCAUGCAGG-OH5′
p3SLR30
5′ppp-GGAUCGAUCGAUCGAUCGGCAUCGAUCGGC U U
3′OH-CCUAGCUAGCUAGCUAGCCGUAGCUAGCCG G C
OHSLR30
5′OH-GGAUCGAUCGAUCGAUCGGCAUCGAUCGGC U U
3′OH-CCUAGCUAGCUAGCUAGCCGUAGCUAGCCG G C

Figure S2

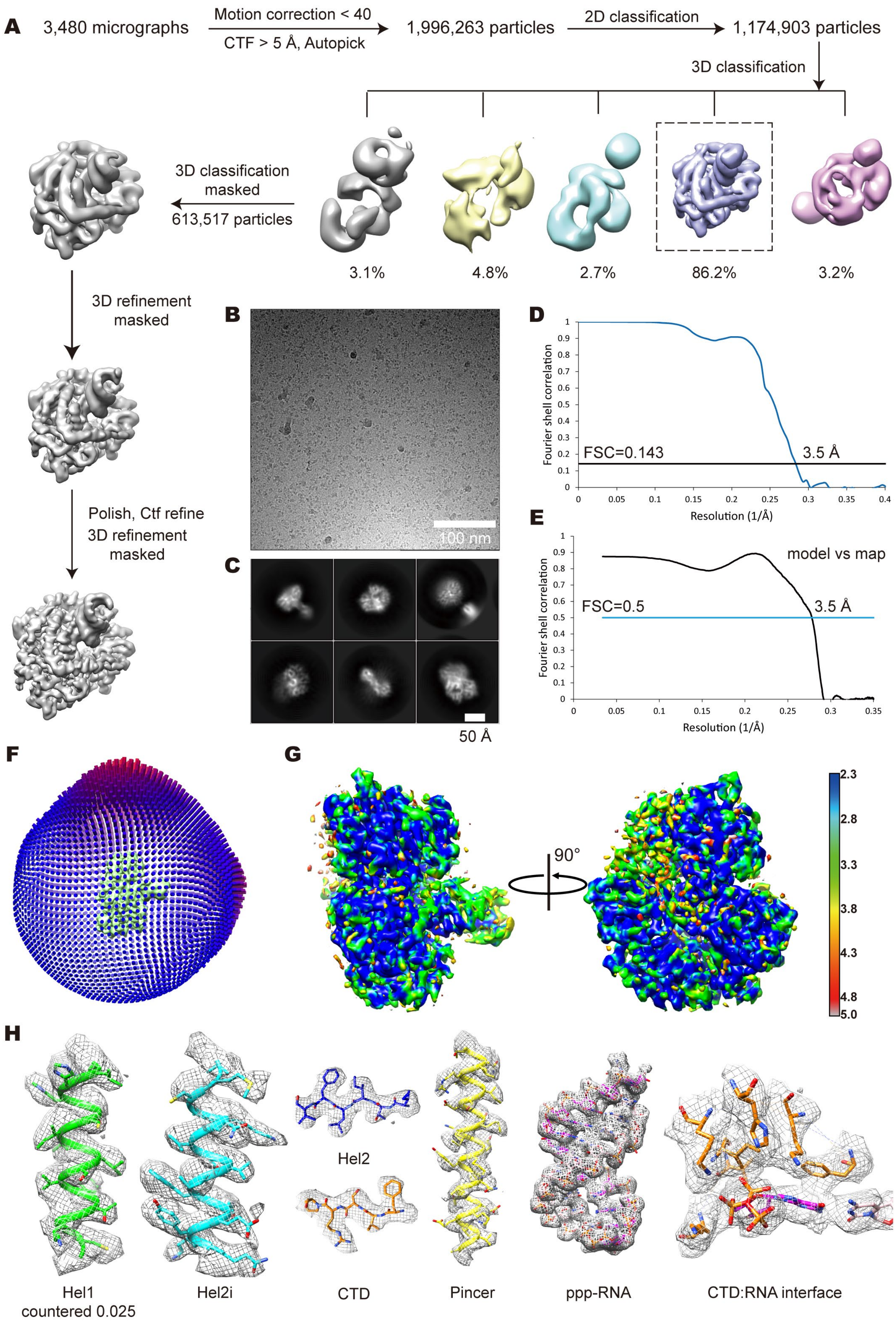
A
3,480 micrographs
Motion correction < 40
CTF > 5 Å, Autopick
1,996,263 particles
2D classification
1,174,903 particles
3D classification
3.1%
4.8%
2.7%
86.2%
3.2%
3D classification masked
613,517 particles
3D refinement masked
Polish, Ctf refine
3D refinement masked
B
100 nm
C
50 Å
D
Fourier shell correlation
FSC=0.143
3.5 Å
Resolution (1/Å)
E
Fourier shell correlation
model vs map
FSC=0.5
3.5 Å
Resolution (1/Å)
F
G
90°
2.3
2.8
3.3
3.8
4.3
4.8
5.0
H
Hel2
Hel1
countered 0.025
Hel2i
CTD
Pincer
ppp-RNA
CTD:RNA interface

Figure S3

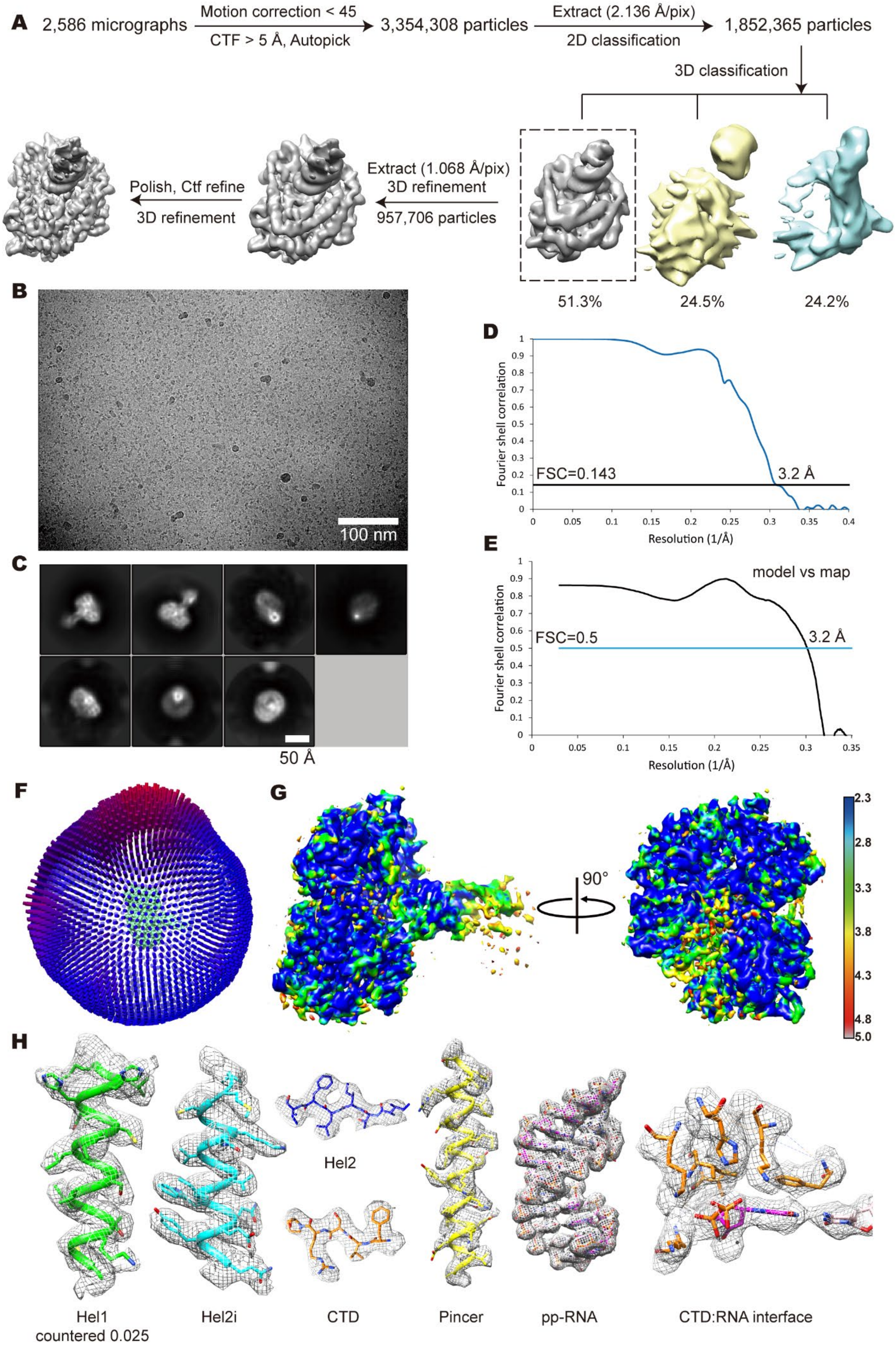

A
2,586 micrographs
Motion correction < 45
CTF > 5 Å, Autopick
3,354,308 particles
Extract (2.136 Å/pix)
2D classification
1,852,365 particles
3D classification
Extract (1.068 Å/pix)
3D refinement
957,706 particles
Polish, Ctf refine
3D refinement
51.3%
24.5%
24.2%
B
100 nm
C
50 Å
D
Fourier shell correlation
FSC=0.143
3.2 Å
Resolution (1/Å)
E
model vs map
Fourier shell correlation
FSC=0.5
3.2 Å
Resolution (1/Å)
F
G
90°
2.3
2.8
3.3
3.8
4.3
4.8
5.0
H
Hel2
Hel1
countered 0.025
Hel2i
CTD
Pincer
pp-RNA
CTD:RNA interface

Figure S4

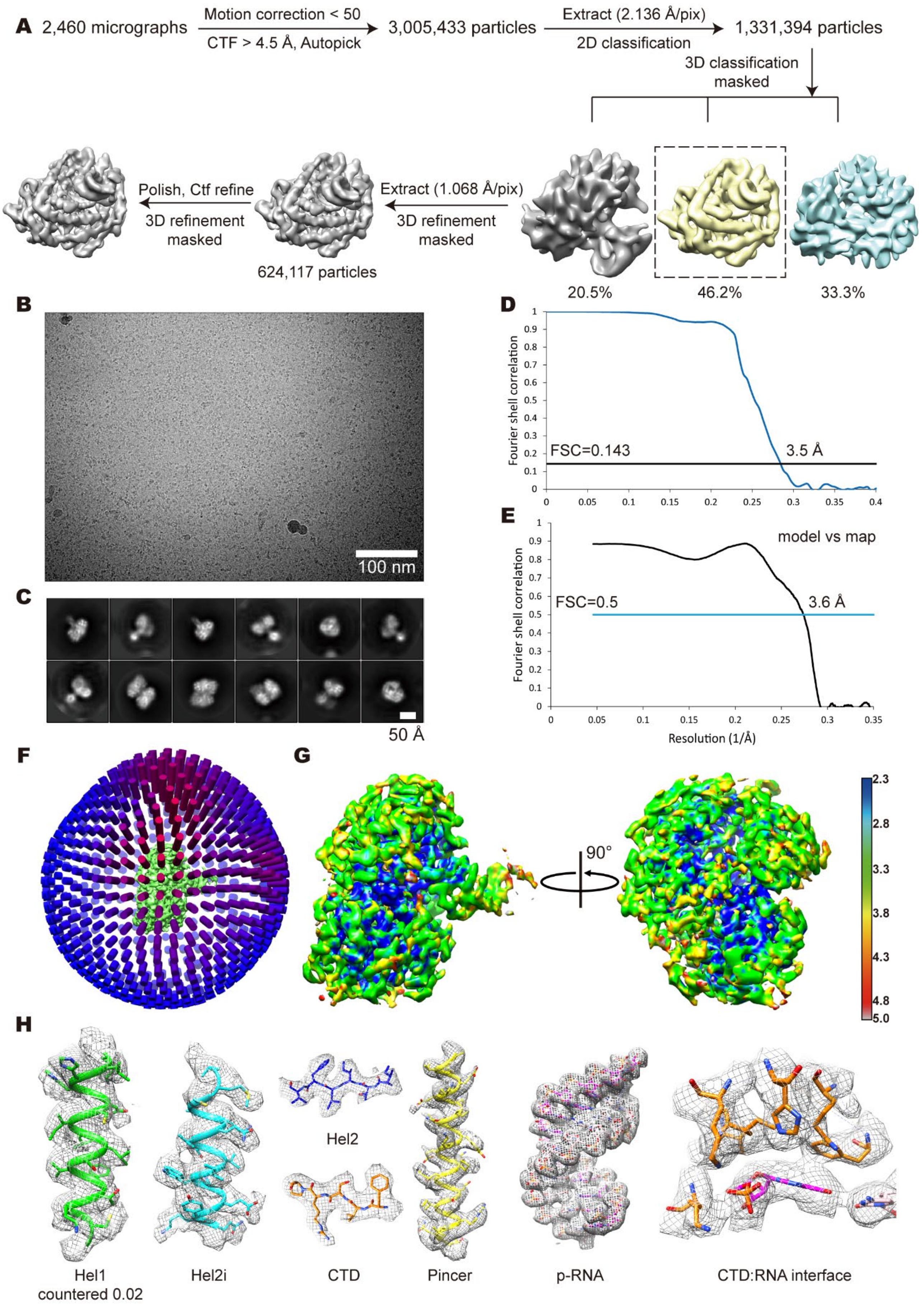

A 2,460 micrographs
Motion correction < 50
CTF > 4.5 Å, Autopick
3,005,433 particles
Extract (2.136 Å/pix)
2D classification
1,331,394 particles
3D classification masked
20.5%
46.2%
33.3%
Extract (1.068 Å/pix)
3D refinement masked
624,117 particles
Polish, Ctf refine
3D refinement masked
B
100 nm
C
50 Å
D
Fourier shell correlation
FSC=0.143
3.5 Å
E
Fourier shell correlation
model vs map
FSC=0.5
3.6 Å
Resolution (1/Å)
F
G
90°
2.3
2.8
3.3
3.8
4.3
4.8
5.0
H
Hel2
Hel1 countered 0.02
Hel2i
CTD
Pincer
p-RNA
CTD:RNA interface

Figure S5

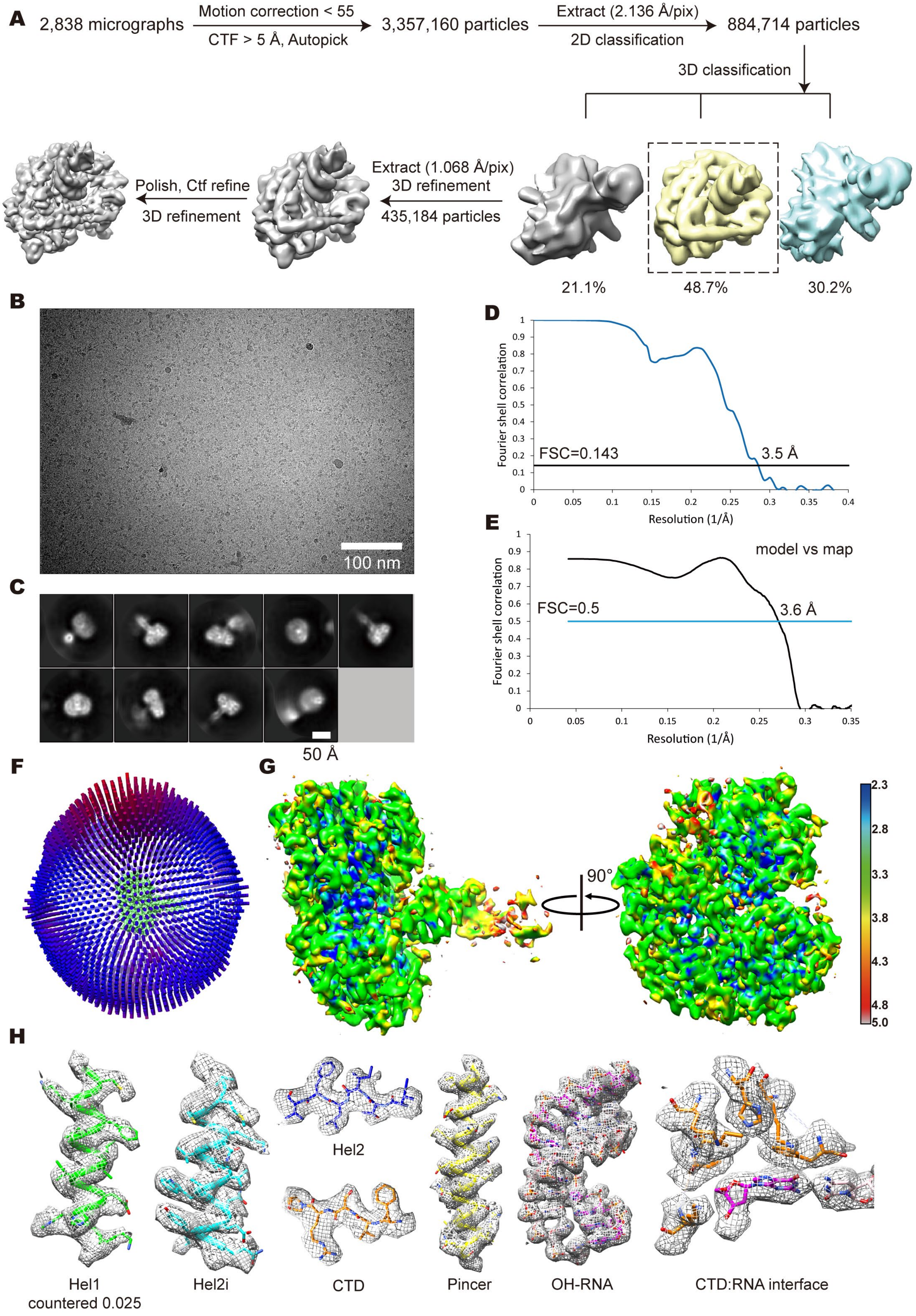

A
2,838 micrographs
Motion correction < 55
CTF > 5 Å, Autopick
3,357,160 particles
Extract (2.136 Å/pix)
2D classification
884,714 particles
3D classification
21.1%
48.7%
30.2%
Extract (1.068 Å/pix)
3D refinement
435,184 particles
Polish, Ctf refine
3D refinement
B
100 nm
C
50 Å
D
FSC=0.143
3.5 Å
Fourier shell correlation
Resolution (1/Å)
E
model vs map
FSC=0.5
3.6 Å
Fourier shell correlation
Resolution (1/Å)
F
G
90°
2.3
2.8
3.3
3.8
4.3
4.8
5.0
H
Hel2
Hel1
countered 0.025
Hel2i
CTD
Pincer
OH-RNA
CTD:RNA interface

Figure S6

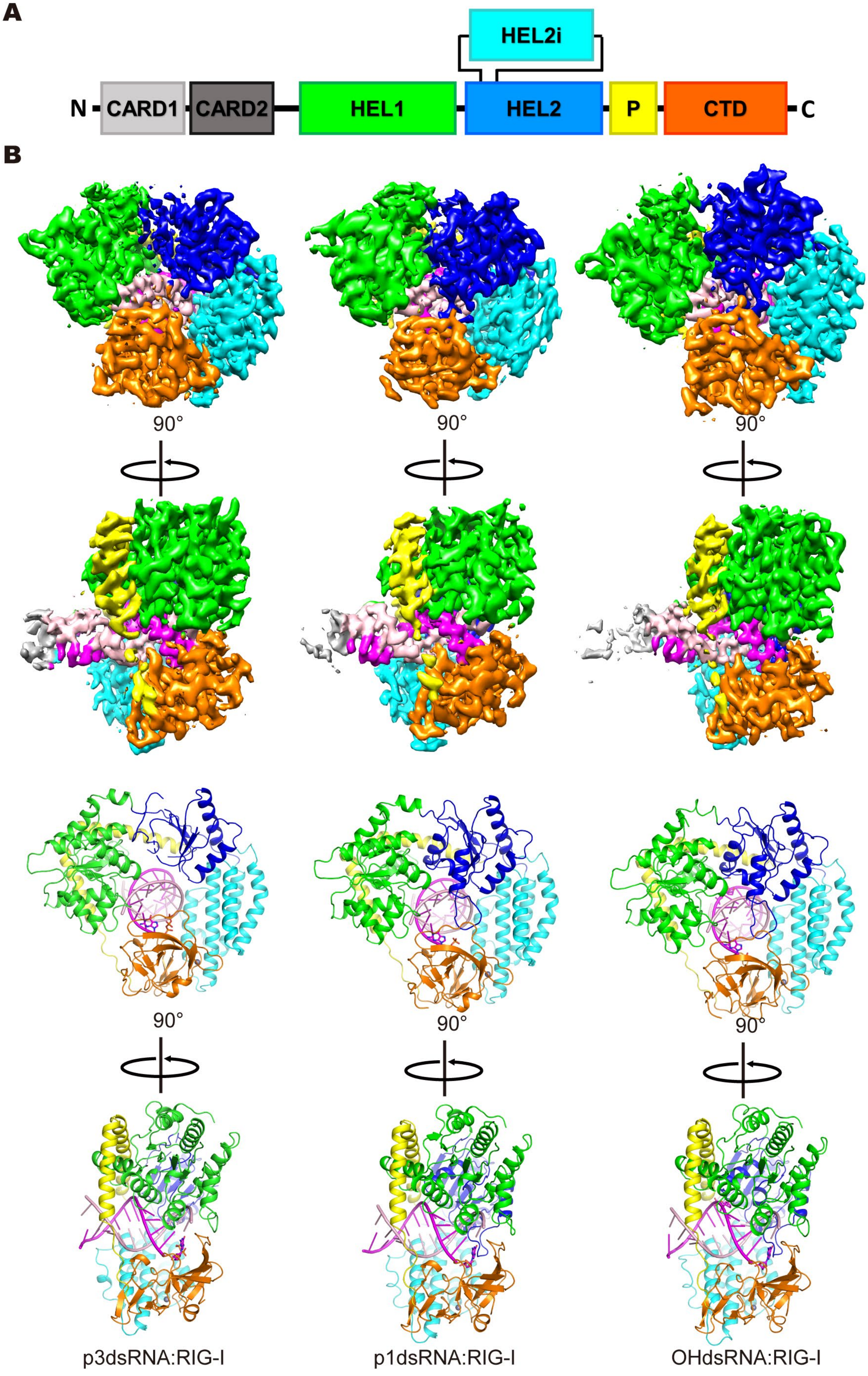
A
HEL2i
N
CARD1
CARD2
HEL1
HEL2
P
CTD
C
B
90°
90°
90°
90°
90°
90°
p3dsRNA:RIG-I
p1dsRNA:RIG-I
OHdsRNA:RIG-I

Figure S7

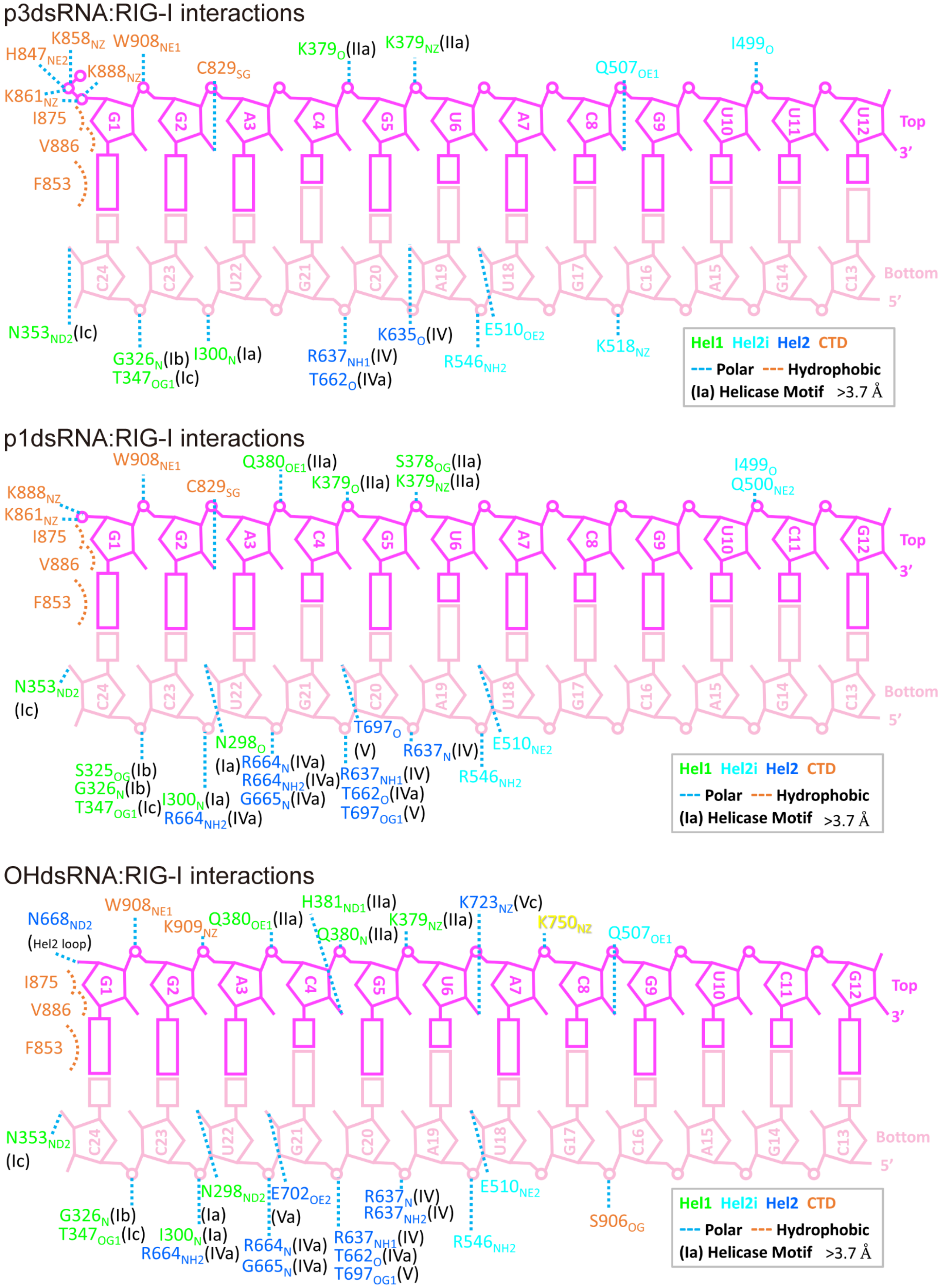
p3dsRNA:RIG-I interactions
K858NZ
W908NE1
H847NE2
K888NZ
K861NZ
I875
V886
F853
C829SG
K379O(IIa)
K379NZ(IIa)
Q507OE1
I499O
G1
G2
A3
C4
G5
U6
A7
C8
G9
U10
U11
U12
Top
3'
C24
C23
U22
G21
C20
A19
U18
G17
C16
A15
G14
C13
Bottom
5'
N353ND2(Ic)
G326N(Ib)
T347OG1(Ic)
I300N(Ia)
R637NH1(IV)
T662O(IVa)
K635O(IV)
E510OE2
R546NH2
K518NZ
Hel1 Hel2i Hel2 CTD
Polar
Hydrophobic
(Ia) Helicase Motif
>3.7 Å
p1dsRNA:RIG-I interactions
W908NE1
Q380OE1(IIa)
S378OG(IIa)
K379O(IIa)
K379NZ(IIa)
I499O
Q500NE2
K888NZ
K861NZ
C829SG
I875
V886
F853
N353ND2
(Ic)
S325OG(Ib)
G326N(Ib)
T347OG1(Ic)
I300N(Ia)
R664NH2(IVa)
N298O
(Ia)
R664N(IVa)
R664NH2(IVa)
G665N(IVa)
T697O
(V)
R637NH1(IV)
T662O(IVa)
T697OG1(V)
R637N(IV)
E510NE2
R546NH2
Hel1 Hel2i Hel2 CTD
Polar
Hydrophobic
(Ia) Helicase Motif
>3.7 Å
OHdsRNA:RIG-I interactions
N668ND2
(Hel2 loop)
W908NE1
K909NZ
Q380OE1(IIa)
H381ND1(IIa)
Q380N(IIa)
K379NZ(IIa)
K723NZ(Vc)
K750NZ
Q507OE1
I875
V886
F853
N353ND2
(Ic)
G326N(Ib)
T347OG1(Ic)
I300N(Ia)
R664NH2(IVa)
N298ND2
(Ia)
E702OE2
(Va)
R664N(IVa)
G665N(IVa)
R637N(IV)
R637NH2(IV)
R637NH1(IV)
T662O(IVa)
T697OG1(V)
E510NE2
R546NH2
S906OG
Hel1 Hel2i Hel2 CTD
Polar
Hydrophobic
(Ia) Helicase Motif
>3.7 Å

Figure S8

p3dsRNA:RIG-I

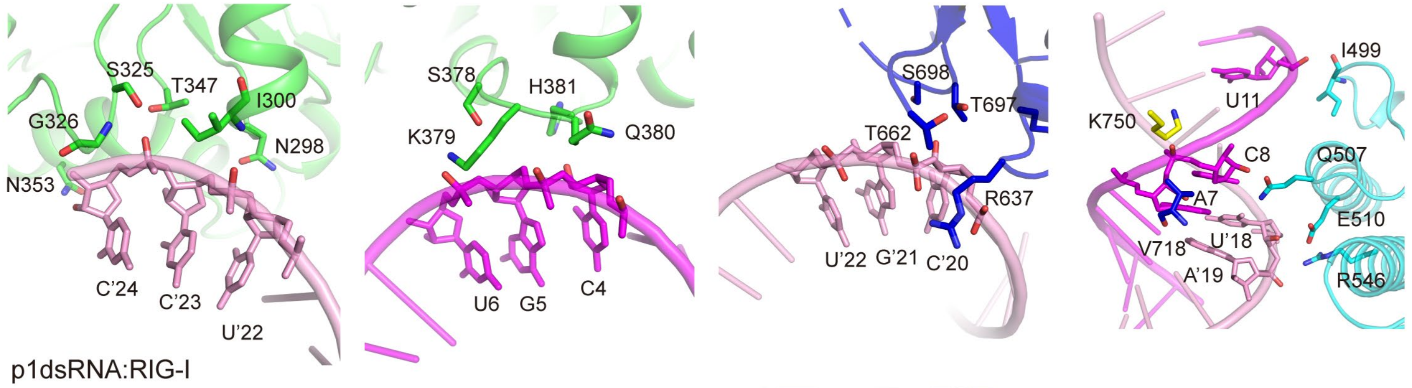

p1dsRNA:RIG-I

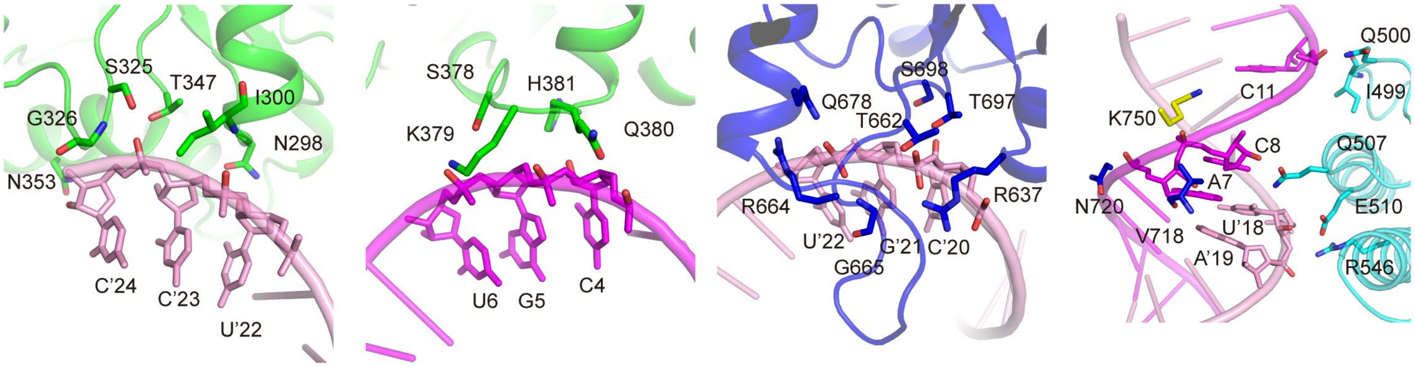

OHdsRNA:RIG-I

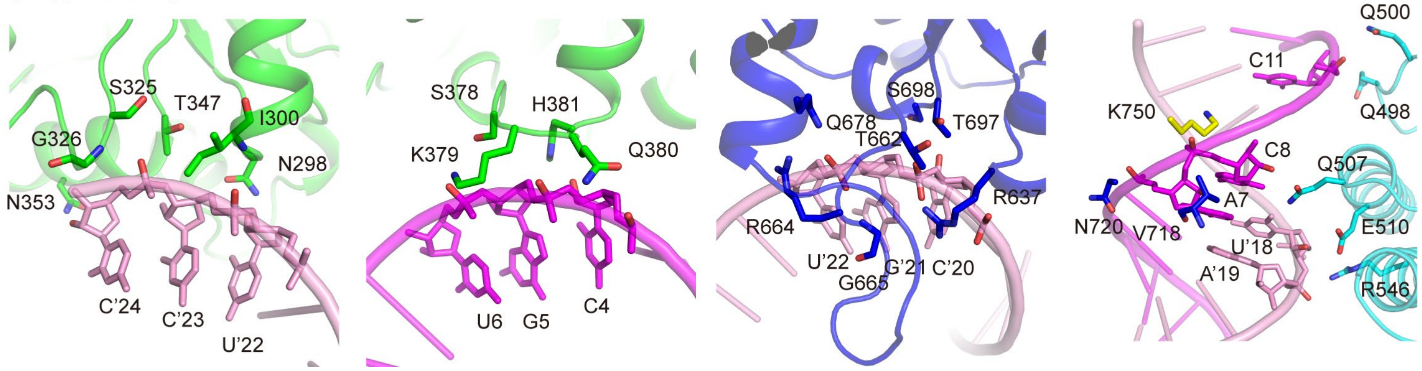

Figure S9

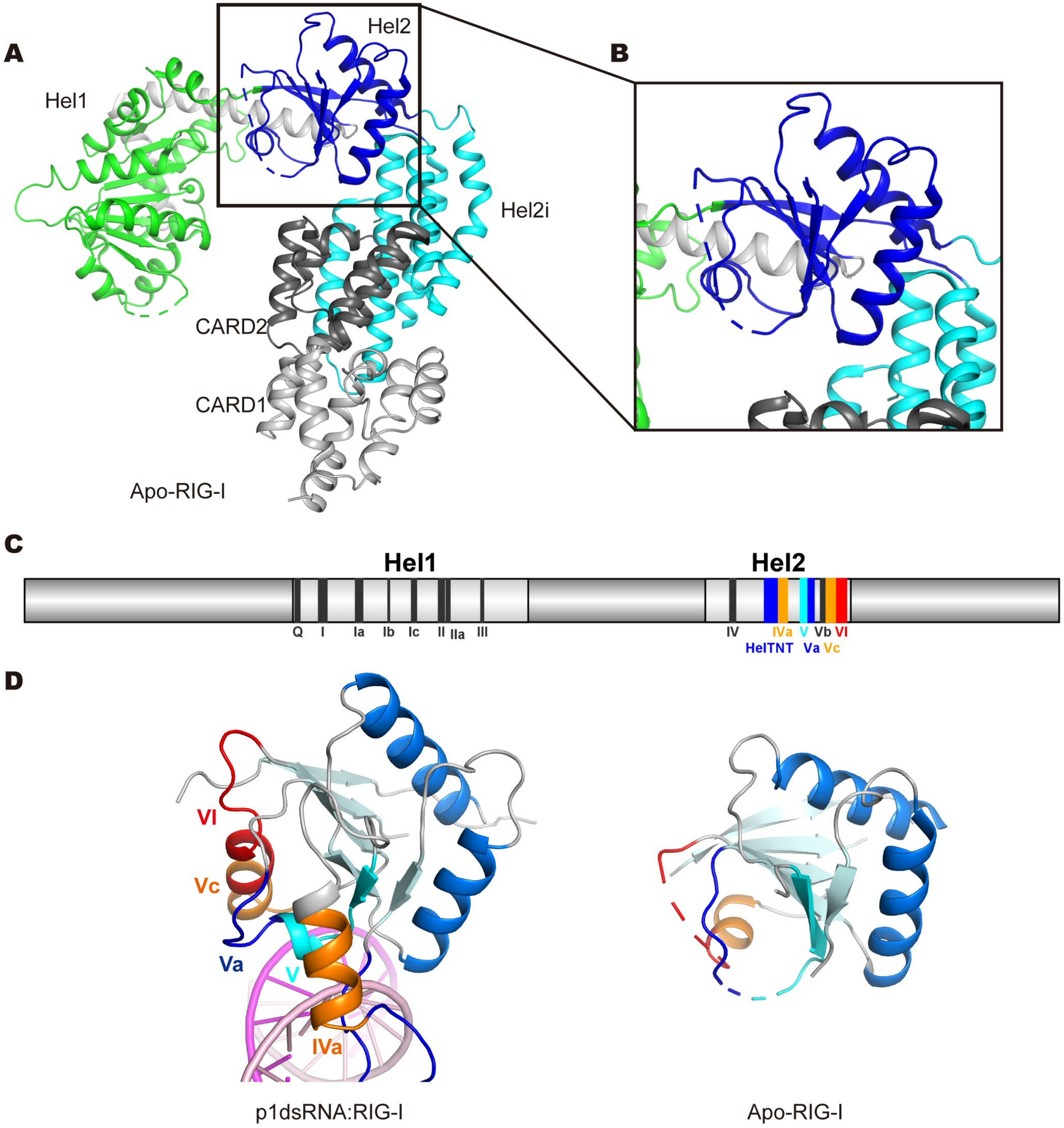
A
Hel2
Hel1
Hel2i
CARD2
CARD1
Apo-RIG-I
B
C
Hel1
Hel2
Q
I
Ia
Ib
Ic
II
IIa
III
IV
IVa
V
Vb
VI
HelTNT
Va
Vc
D
VI
Vc
Va
V
IVa
p1dsRNA:RIG-I
Apo-RIG-I

Figure S10

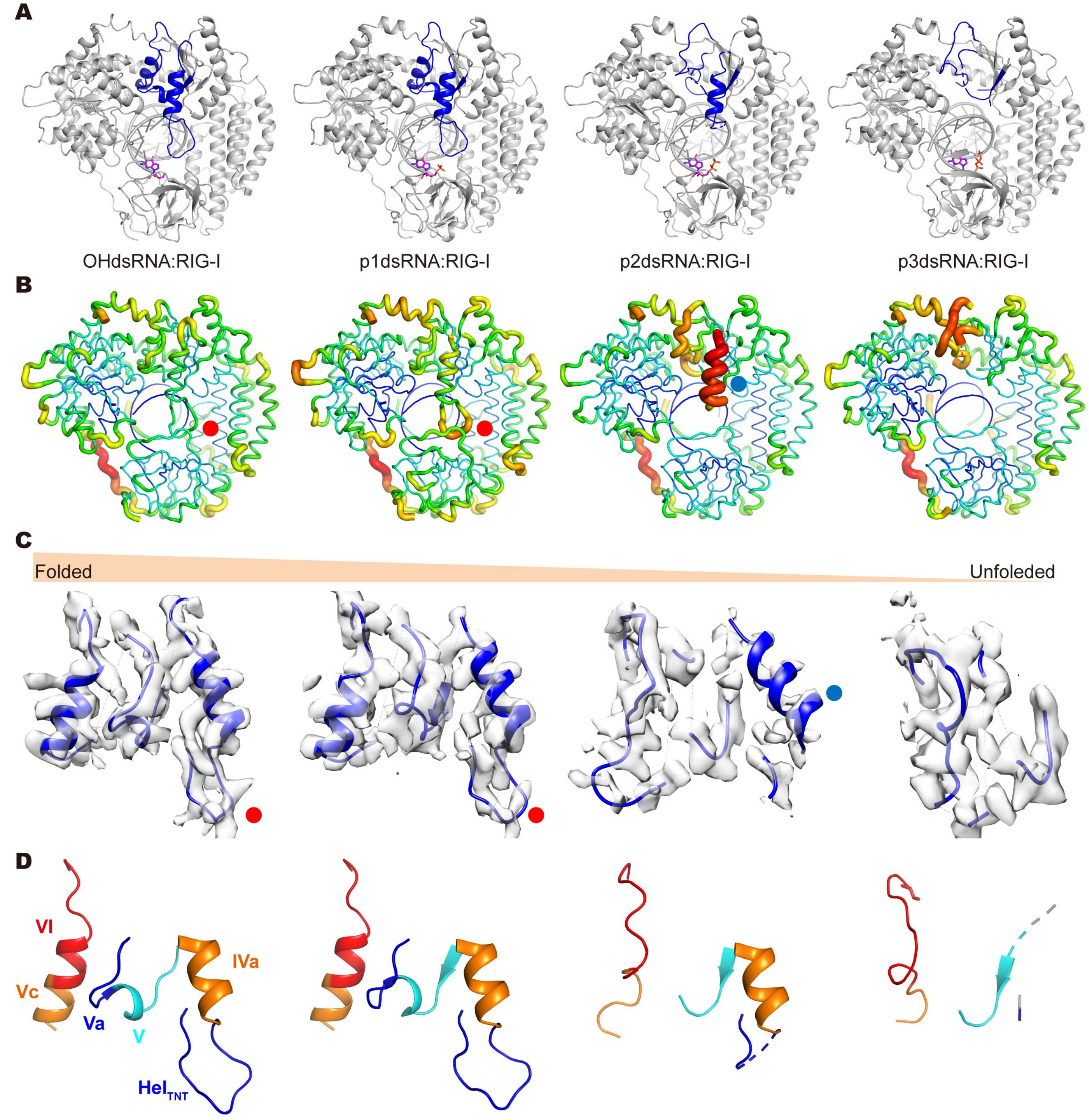
A
OHdsRNA:RIG-I
p1dsRNA:RIG-I
p2dsRNA:RIG-I
p3dsRNA:RIG-I
B
C
Folded
Unfoleded
D
VI
Vc
Va
V
IVa
Hel$_{TNT}$

Figure S11

A

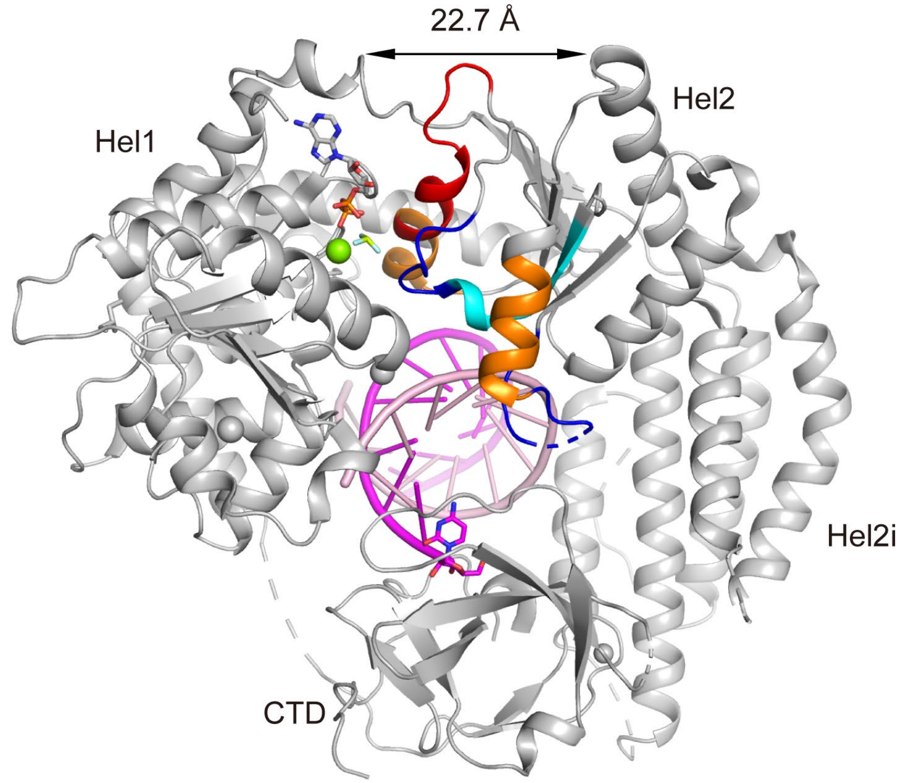

Closed conformation
RIG-I:OH-RNA:ADP-BeF_3
PDB: 5e3h

B

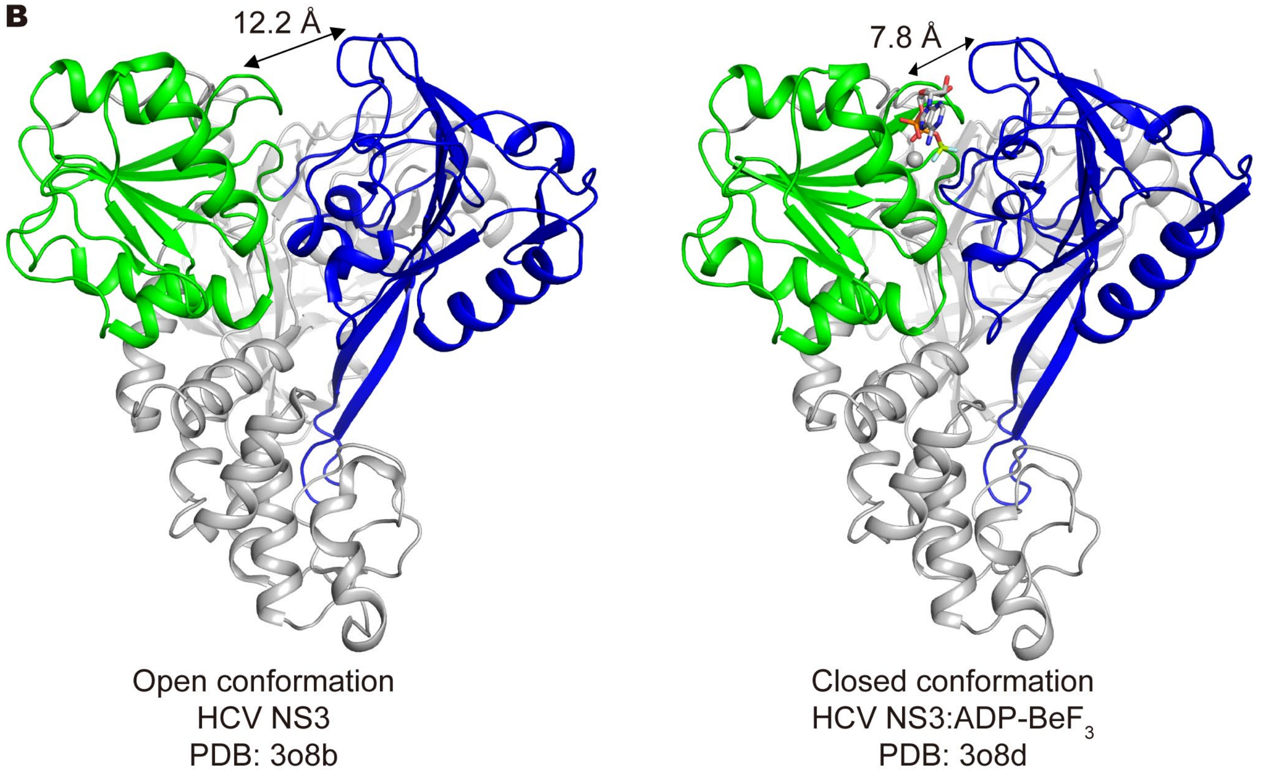

Open conformation
HCV NS3
PDB: 3o8b

Closed conformation
HCV NS3:ADP-BeF_3
PDB: 3o8d

Figure S12

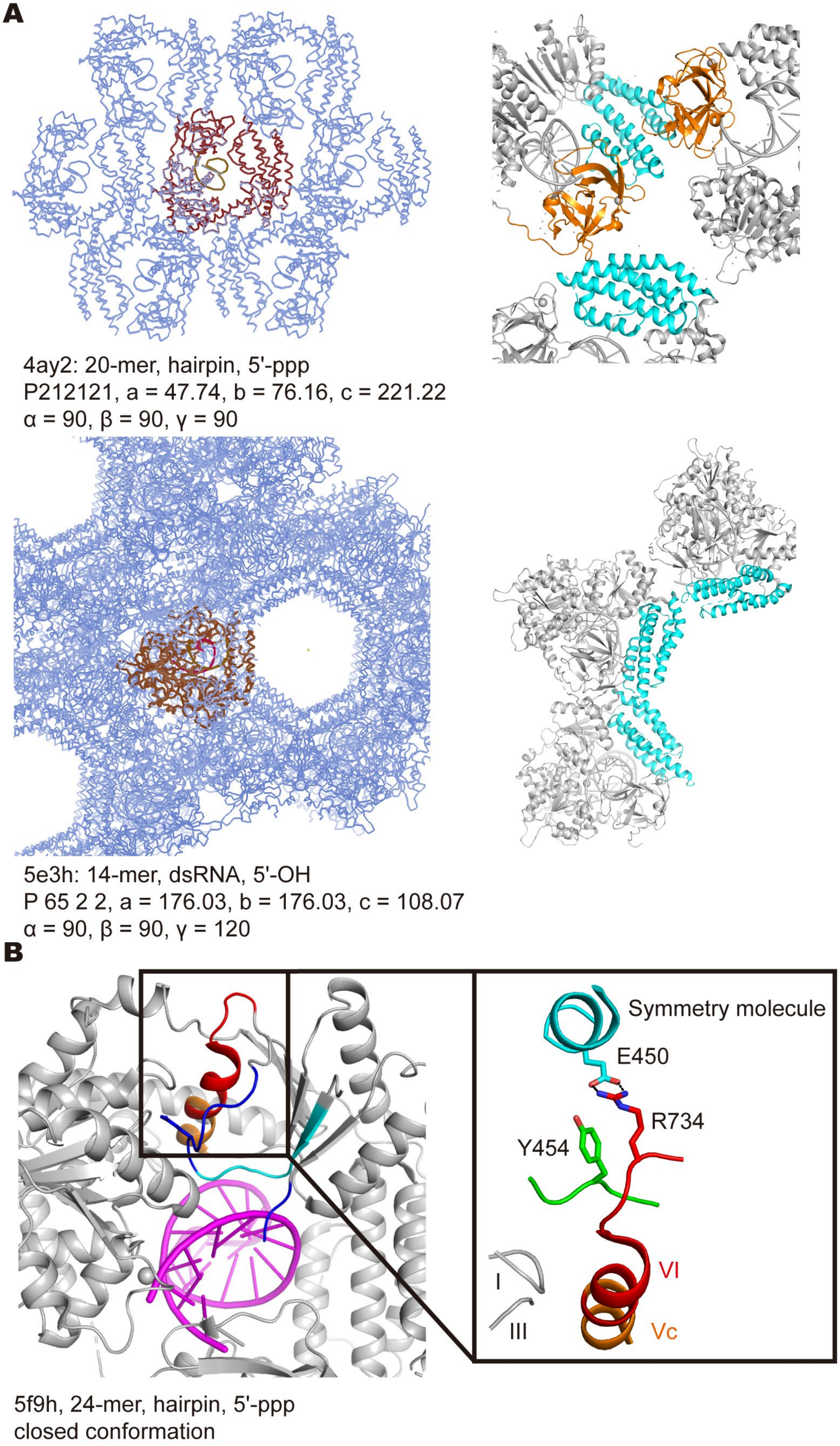
A
4ay2: 20-mer, hairpin, 5'-ppp
P212121, a = 47.74, b = 76.16, c = 221.22
α = 90, β = 90, γ = 90
5e3h: 14-mer, dsRNA, 5'-OH
P 65 2 2, a = 176.03, b = 176.03, c = 108.07
α = 90, β = 90, γ = 120
B
Symmetry molecule
E450
R734
Y454
VI
I
III
Vc
5f9h, 24-mer, hairpin, 5'-ppp
closed conformation

Figure S13

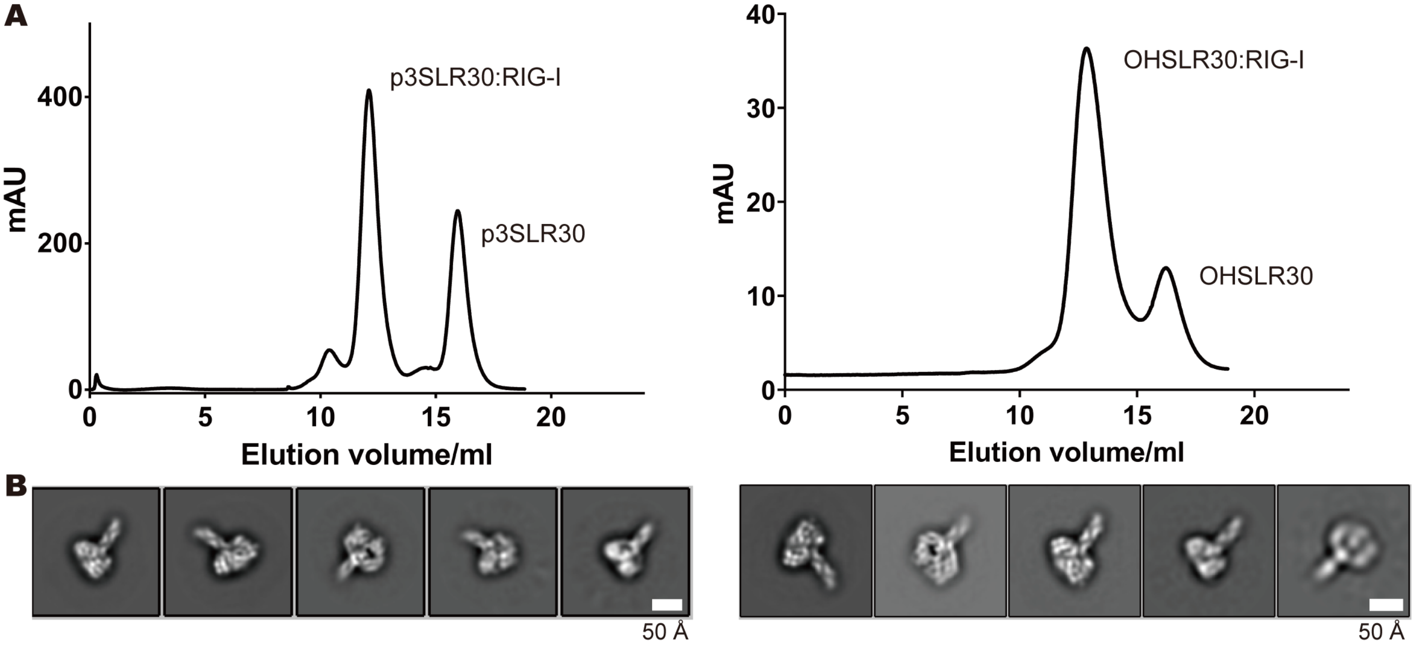

2D class averages of p3SLR30:RIG-I without ATP and Mg^{2+}

2D class averages of OHSLR30:RIG-I without ATP and Mg^{2+}

Figure S14

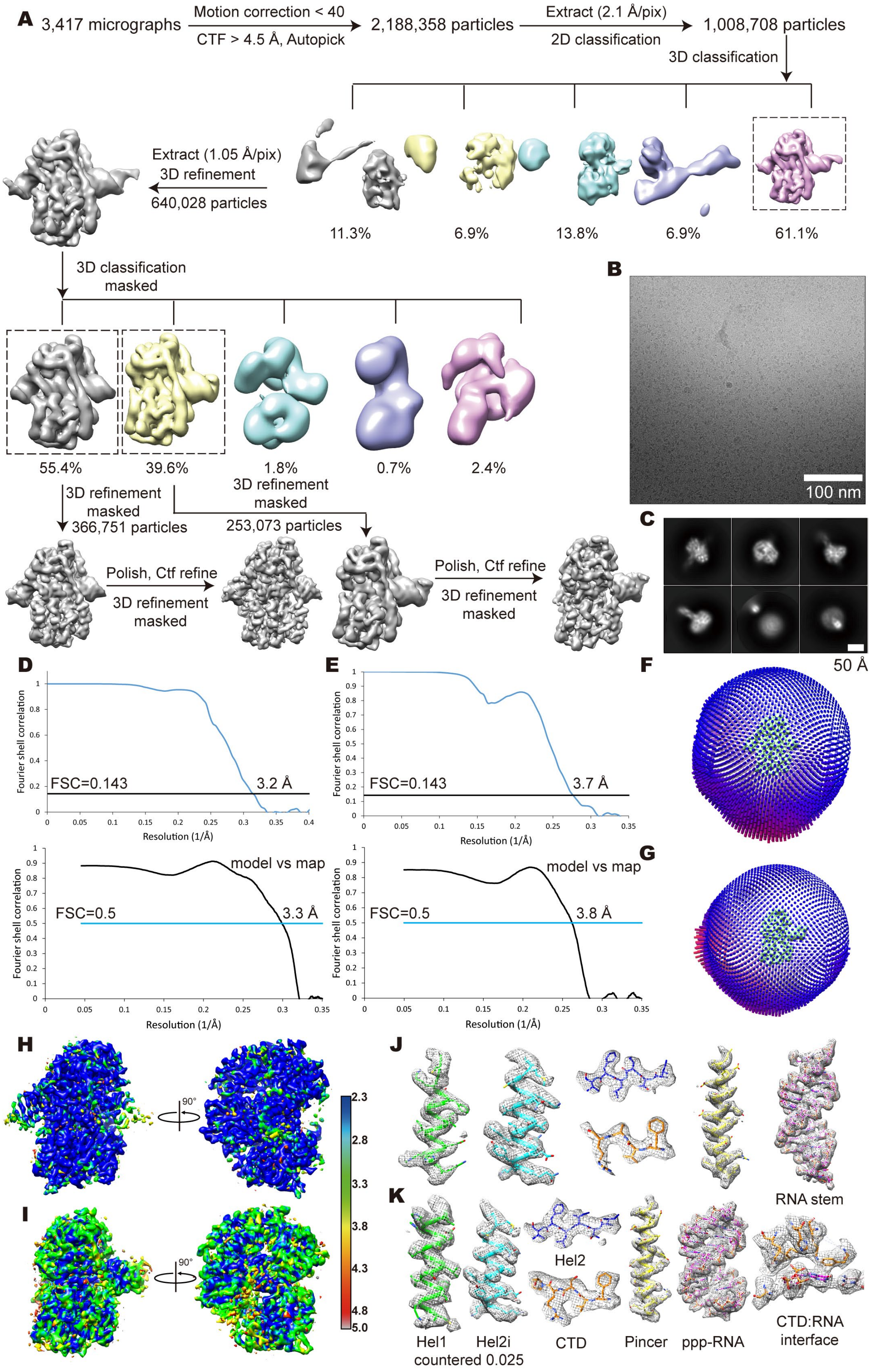

A 3,417 micrographs
Motion correction < 40
CTF > 4.5 Å, Autopick
2,188,358 particles
Extract (2.1 Å/pix)
2D classification
1,008,708 particles
3D classification
11.3%
6.9%
13.8%
6.9%
61.1%
Extract (1.05 Å/pix)
3D refinement
640,028 particles
3D classification
masked
55.4%
39.6%
1.8%
0.7%
2.4%
3D refinement
masked
366,751 particles
3D refinement
masked
253,073 particles
Polish, Ctf refine
3D refinement
masked
Polish, Ctf refine
3D refinement
masked
B
100 nm
C
50 Å
D
Fourier shell correlation
FSC=0.143
3.2 Å
Resolution (1/Å)
model vs map
FSC=0.5
3.3 Å
E
FSC=0.143
3.7 Å
model vs map
FSC=0.5
3.8 Å
F
G
H
90°
2.3
2.8
3.3
3.8
4.3
4.8
5.0
I
J
RNA stem
K
Hel2
Hel1
countered 0.025
Hel2i
CTD
Pincer
ppp-RNA
CTD:RNA
interface

Figure S15

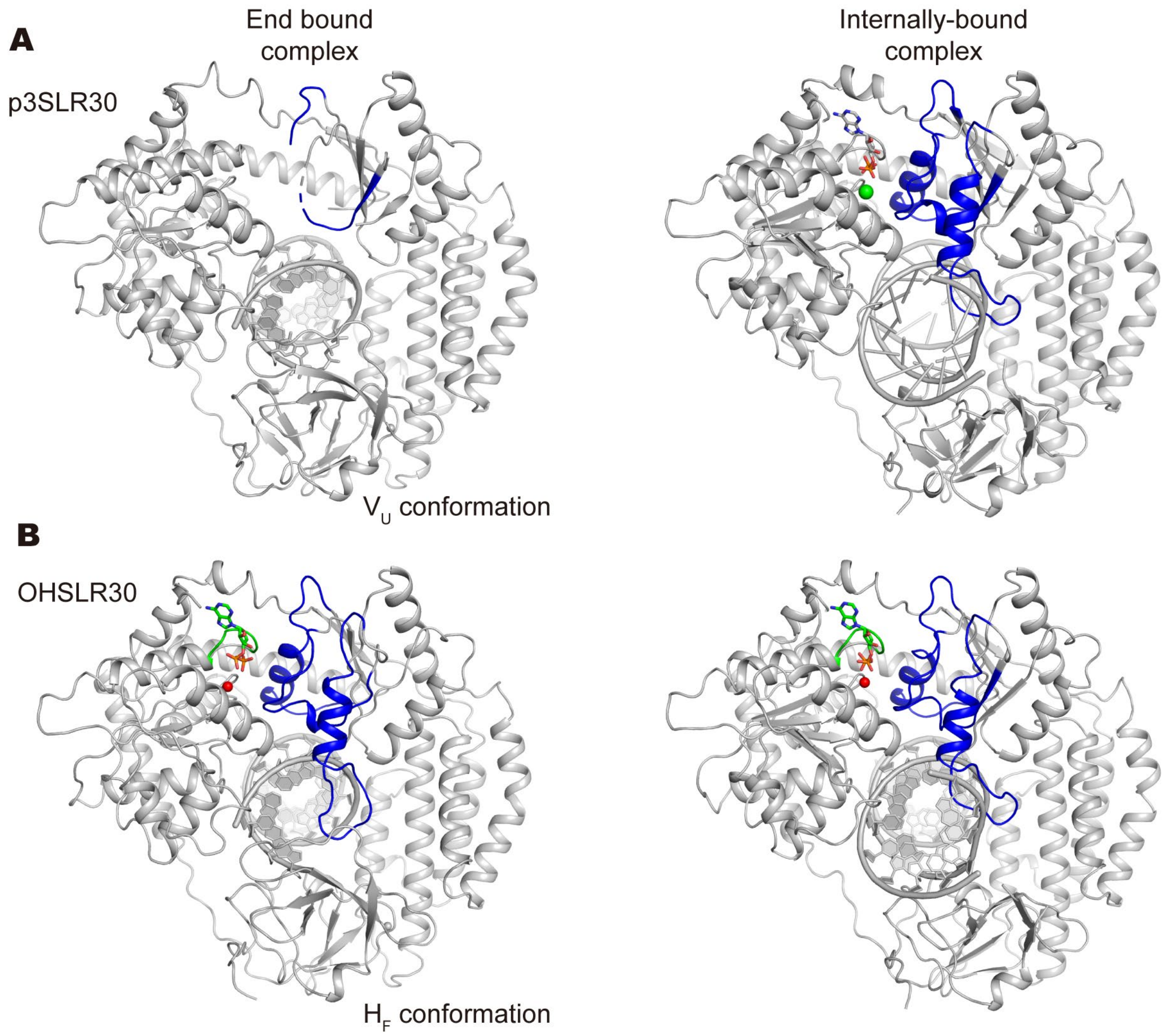

A
End bound complex
Internally-bound complex
p3SLR30
V_U conformation
B
OHSLR30
H_F conformation

Figure S16

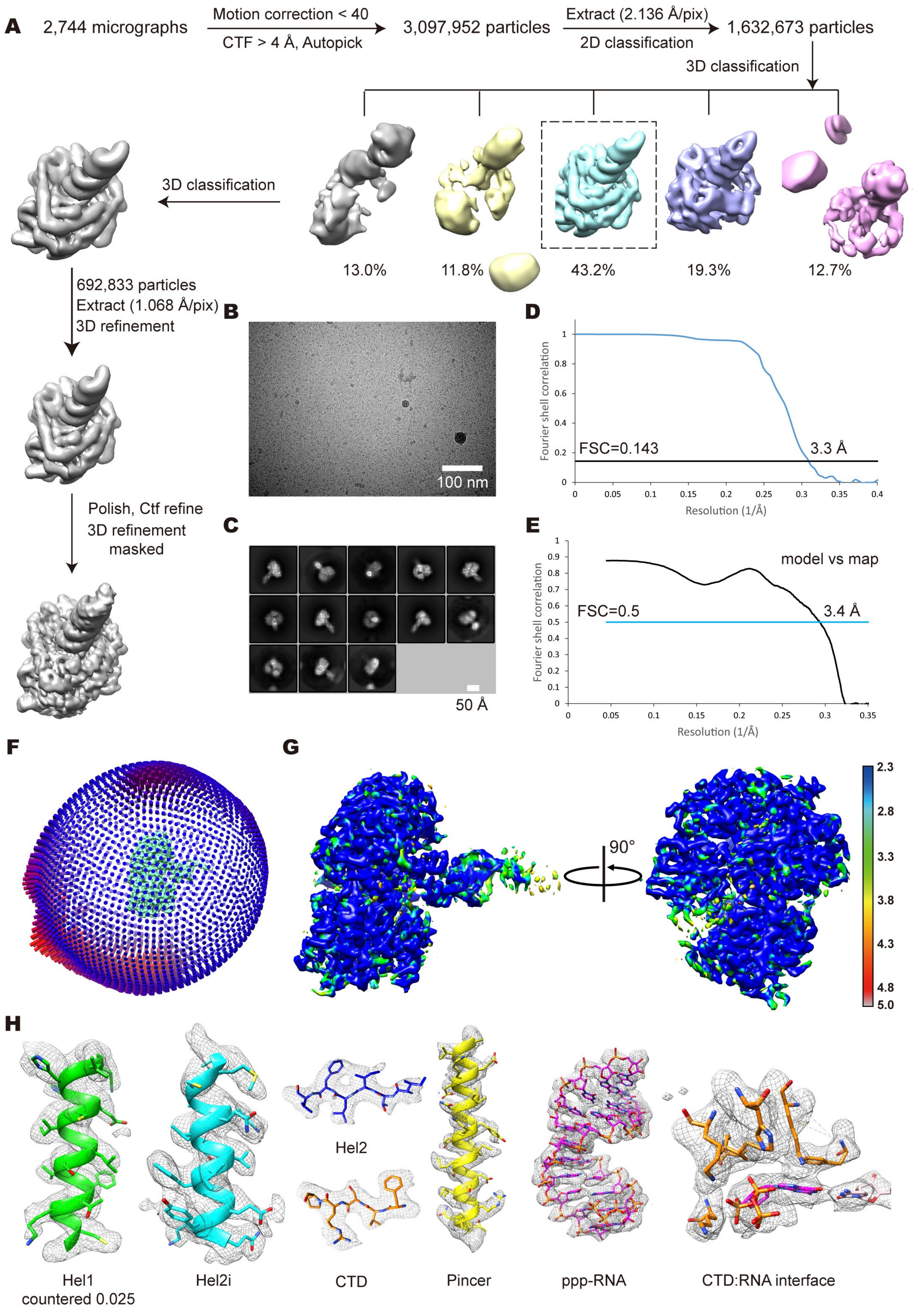

A
2,744 micrographs
Motion correction < 40
CTF > 4 Å, Autopick
3,097,952 particles
Extract (2.136 Å/pix)
2D classification
1,632,673 particles
3D classification
13.0%
11.8%
43.2%
19.3%
12.7%
3D classification
692,833 particles
Extract (1.068 Å/pix)
3D refinement
Polish, Ctf refine
3D refinement
masked
B
100 nm
C
50 Å
D
Fourier shell correlation
FSC=0.143
3.3 Å
Resolution (1/Å)
E
model vs map
FSC=0.5
3.4 Å
Fourier shell correlation
Resolution (1/Å)
F
G
90°
2.3
2.8
3.3
3.8
4.3
4.8
5.0
H
Hel2
Hel1
countered 0.025
Hel2i
CTD
Pincer
ppp-RNA
CTD:RNA interface

Figure S17

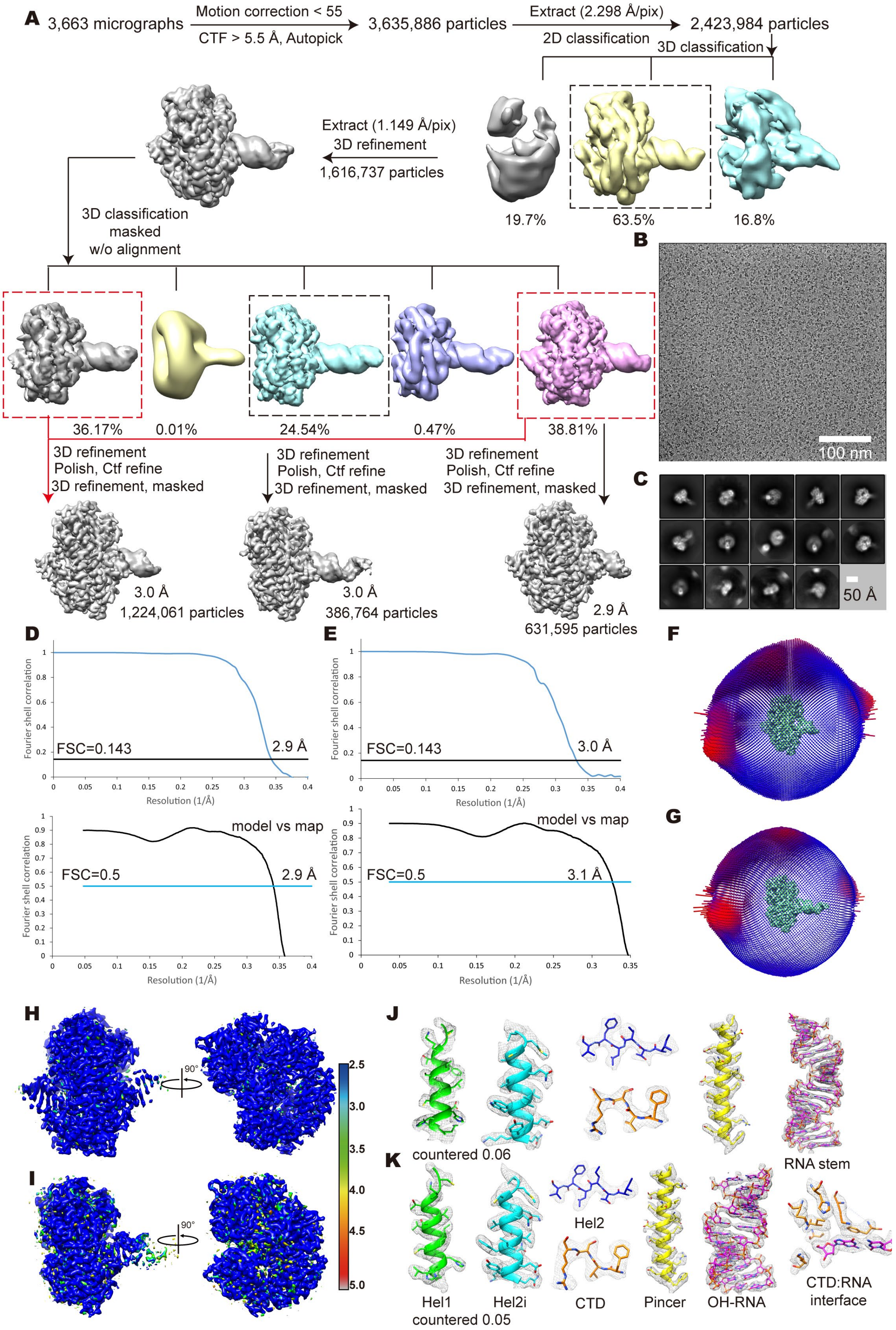

A
3,663 micrographs
Motion correction < 55
CTF > 5.5 Å, Autopick
3,635,886 particles
Extract (2.298 Å/pix)
2D classification
2,423,984 particles
3D classification
19.7%
63.5%
16.8%
Extract (1.149 Å/pix)
3D refinement
1,616,737 particles
3D classification
masked
w/o alignment
36.17%
0.01%
24.54%
0.47%
38.81%
3D refinement
Polish, Ctf refine
3D refinement, masked
3.0 Å
1,224,061 particles
386,764 particles
2.9 Å
631,595 particles
B
100 nm
C
50 Å
D
E
Fourier shell correlation
Resolution (1/Å)
FSC=0.143
3.0 Å
model vs map
FSC=0.5
3.1 Å
F
G
H
I
90°
2.5
3.0
3.5
4.0
4.5
5.0
J
K
countered 0.06
RNA stem
Hel2
Hel1
countered 0.05
Hel2i
CTD
Pincer
OH-RNA
CTD:RNA
interface

Figure S18

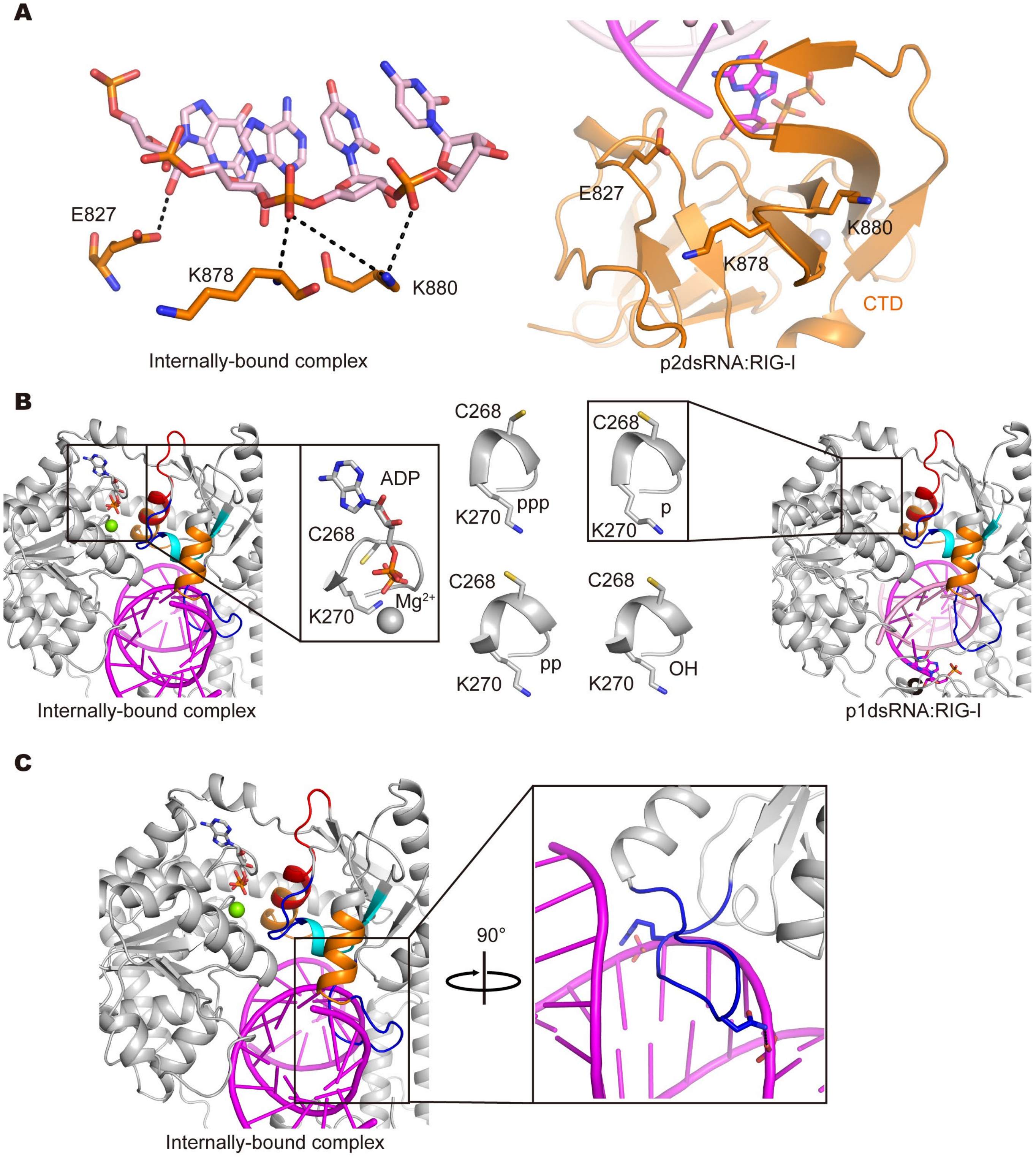
A
E827
K878
K880
Internally-bound complex
E827
K880
K878
CTD
p2dsRNA:RIG-I
B
ADP
C268
K270
Mg2+
C268
K270
ppp
C268
K270
p
C268
K270
pp
C268
K270
OH
Internally-bound complex
p1dsRNA:RIG-I
C
90°
Internally-bound complex

Figure S19

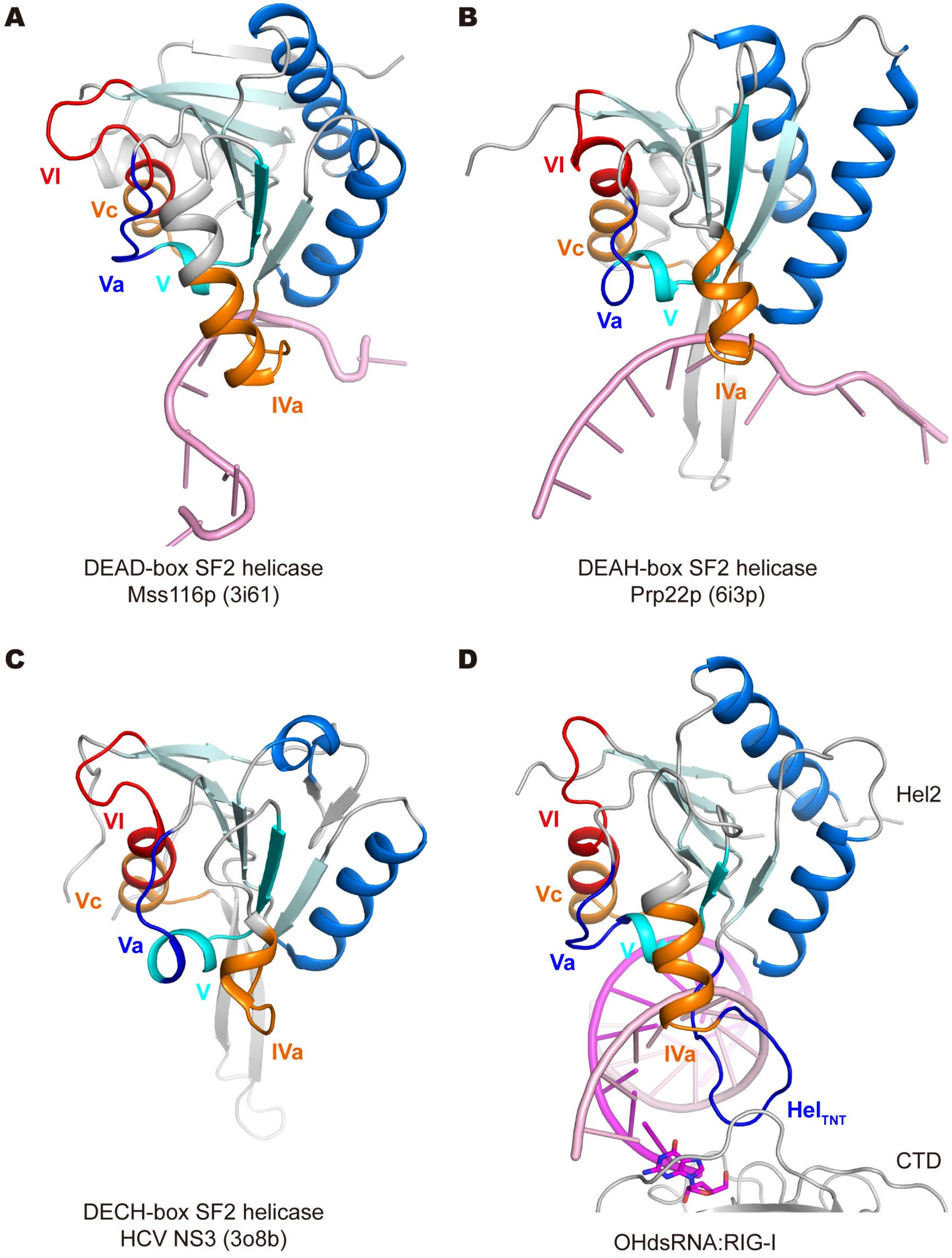
A
VI
Vc
Va
V
IVa
DEAD-box SF2 helicase
Mss116p (3i61)
B
VI
Vc
Va
V
IVa
DEAH-box SF2 helicase
Prp22p (6i3p)
C
VI
Vc
Va
V
IVa
DECH-box SF2 helicase
HCV NS3 (3o8b)
D
VI
Vc
Va
V
IVa
Hel2
HelTNT
CTD
OHdsRNA:RIG-I

Figure S1. RNA duplexes used in this study. Related to Figure 1, 5 and STAR Methods.

Figure S2. Cryo-EM analysis of the p3dsRNA:RIG-I complex. Related to Figure 1 and STAR Methods.

(A) Flow chart of cryo-EM data processing.

(B) Representative micrograph.

(C) Representative 2D class averages from Relion 2D classification.

(D) Fourier shell correlation (FSC) curve for the p3dsRNA:RIG-I complex.

(E) Fourier shell correlation between the refined model and the map that the model is refined against.

(F) Euler angle distribution of all particles used in the final map reconstruction. Final map shown in green. Each orientation is represented by a cylinder, with each cylinder's height and color (from blue to red) proportional to the number of particles for that specific direction.

(G) Local resolution display of p3dsRNA:RIG-I complex reconstruction.

(H) Representative local densities contoured at 0.025

Figure S3. Cryo-EM analysis of the p2dsRNA:RIG-I complex. Related to Figure 1 and STAR Methods.

(A) Flow chart of cryo-EM data processing.

(B) Representative micrograph.

(C) Representative 2D class averages from Relion 2D classification.

(D) Fourier shell correlation (FSC) curve for the p2dsRNA:RIG-I complex.

(E) Fourier shell correlation between the refined model and the map.

(F) Euler angle distribution of all particles used in the final map reconstruction.

(G) Local resolution display of p2dsRNA:RIG-I complex reconstruction.

(H) Representative local densities contoured at 0.025

Figure S4. Cryo-EM analysis of the p1dsRNA:RIG-I complex. Related to Figure 1 and STAR Methods.

(A) Flow chart of cryo-EM data processing.

(B) Representative micrograph.

(C) Representative 2D class averages from Relion 2D classification.

(D) Fourier shell correlation (FSC) curve for the p1dsRNA:RIG-I complex.

(E) Fourier shell correlation between the refined model and the map.

(F) Euler angle distribution of all particles used in the final map reconstruction.

(G) Local resolution display of p1dsRNA:RIG-I complex reconstruction.

(H) Representative local densities contoured at 0.02

Figure S5. Cryo-EM analysis of the OHdsRNA:RIG-I complex. Related to Figure 1 and STAR Methods.

(A) Flow chart of cryo-EM data processing.

(B) Representative micrograph.

(C) Representative 2D class averages from Relion 2D classification.

(D) Fourier shell correlation (FSC) curve for the OHdsRNA:RIG-I complex.

(E) Fourier shell correlation between the refined model and the map.

(F) Euler angle distribution of all particles used in the final map reconstruction.

(G) Local resolution display of OHdsRNA:RIG-I complex reconstruction.

(H) Representative local densities contoured at 0.025

Figure S6. Overview of the p3dsRNA:RIG-I, p1dsRNA:RIG-I and OHdsRNA:RIG-I complexes. Related to Figure 1.

(A) Schematic representation of the RIG-I protein.

(B) Cryo-EM maps and overall structures of p3dsRNA:RIG-I, p1dsRNA:RIG-I and OHdsRNA:RIG-I. Starting from the N terminus: The CARDs are invisible in the structure; Hel1 (green), Hel2i (cyan), Hel2 (blue), Pincer (yellow) and CTD (orange) of RIG-I are highlighted. The top and bottom strands of the dsRNA is colored as magenta and pink, respectively. The first nucleotide of top strand is shown as sticks.

Figure S7. The interactions between RNA and RIG-I in the p3dsRNA:RIG-I, p1dsRNA:RIG-I and OHdsRNA:RIG-I complexes, determined with a 3.7 Å cutoff for polar contacts. Related to Figure 1.

Residue F853 stacks on the terminal base pair of the RNA duplex, while I875 and V886 make hydrophobic contacts with the G1 ribose.

Figure S8. Zoom-in views of Hel1-RNA (green), Hel2-RNA (blue) and Hel2i-RNA (cyan) interfaces in p3dsRNA:RIG-I, p1dsRNA:RIG-I and OHdsRNA:RIG-I complexes. Related to Figure 1.

Motifs Ia (N298, I300), Ib (S325, G326) and Ic (T347, N353) of Hel1 make polar contacts with backbone atoms of the bottom strand (U’22-C’24), and motif IIa (S378, K379, Q380 and H381) interacts with backbone atoms in the top strand (C4-U6). Hel2 forms a network of interactions with backbone atoms on the RNA, specifically joining motifs IV (R637), IVa (T662, R664, G665, Q678) and V (T697, S698) with the bottom strand (C’20-U’23), motif Vc (V718, N720) with the top strand. Residues Q498 and Q507 of Hel2i interact with the top strand (C8, C11), while E510 and R546 interact with the bottom strand (U’18-A’19).

Figure S9. Hel2 is unfolded revealed by the apo-RIG-I crystal structure. Related to Figure 2.

(A) The duck apo-RIG-I crystal structure (PDB ID: 4a2w). CARD2 (dark gray) interacts with Hel2i (cyan). The disordered helicase motifs in Hel2 are highlighted in blue.

(B) Zoom-in view of the disordered helicase motifs in Hel2 domain in the duck apo-RIG-I structure.

(C) Schematic representation of helicase motifs in RIG-I.

(D) Comparison of the well-folded helicase motifs in Hel2 in the p1dsRNA24:RIG-I complex structure (left) and the unfolded helicase motifs in the apo-RIG-I structure (right).

Figure S10. Hel2 changes from well-folded to unfolded from RIG-I in complex with host RNA duplexes to viral RNA duplexes. Related to Figure 2 and 3.

(A) Overview of helicase motifs (blue) in the Hel2 domain in the OHdsRNA:RIG-I, p1dsRNA:RIG-I, p2dsRNA:RIG-I and p3dsRNA:RIG-I complexes.

(B) B-factor maps of the OHdsRNA:RIG-I, p1dsRNA:RIG-I, p2dsRNA:RIG-I and p3dsRNA:RIG-I complexes. The red dots indicate Hel_{TNT} in OHdsRNA:RIG-I and p1dsRNA:RIG-I complexes. The blue dot indicates the highly dynamic motif IVa in p2dsRNA:RIG-I complex.

(C) Zoom-in views of helicase motifs in the Hel2 domain of the OHdsRNA:RIG-I, p1dsRNA:RIG-I, p2dsRNA:RIG-I and p3dsRNA:RIG-I complexes with corresponding map densities. The red dots indicate the density of Hel_{TNT} in the p1dsRNA:RIG-I complex and the OHdsRNA:RIG-I complex. The blue dot marks the poor density of motif IVa in the p2dsRNA:RIG-I complex.

(D) Conformational differences of helicase motifs in Hel2 domains of the OHdsRNA:RIG-I, p1dsRNA:RIG-I, p2dsRNA:RIG-I and p3dsRNA:RIG-I complexes.

Figure S11. Open and closed conformation of SF2 helicase. Related to Figure 2 and 3.

(A) RIG-I-dsRNA-$ADPBeF_3$ complex in the SF2 closed conformation (PDB ID: 5e3h). The helicase motifs in Hel2 domain are well-folded. $ADPBeF_3$ is rendered as sticks. The distance between Hel1 and Hel2 is 22.7 Å, while it's 21.7 Å, 22.0 Å, 22.2 Å and 23.0 Å in the OHdsRNA:RIG-I, p1dsRNA:RIG-I, p2dsRNA:RIG-I and p3dsRNA:RIG-I complexes.

(B) The open and closed conformations of a typical SF2 helicase, HCV NS3 (Open, PDB ID: 3o8b; closed, PDB ID: 3o8d). RecA1 in green and RecA2 in blue. $ADPBeF_3$ is rendered as sticks.

Figure S12. Crystal structures of dsRNA:ΔCARD-RIG-I complexes. Related to Figure 4.

(A) Two kinds of molecular packing revealed by the crystal structures. The crystal matrices (left) and molecular interfaces (right) of two types of dsRNA:ΔCARD-RIG-I crystal structures (p3dsRNA:ΔCARD-RIG-I complex, PDB ID: 4ay2; OHdsRNA:ΔCARD-RIG-I complex, PDB ID: 5e3h) are rendered. In the matrices (left), the molecule in the asymmetric unit and the symmetry molecules are highlighted in deep red and blue, respectively. In the right panel showing molecular interfaces, Hel2i and CTD are shown in cyan and orange, respectively.

(B) The interactions between symmetry molecules stabilize motif Vc and VI as an α-helix. Motif Vc and VI are highlighted in orange and red, respectively. The symmetry molecule is rendered in cyan.

Figure S13. RIG-I binds to the terminus of p3SLR30 and OHSLR30 in the absence of ATP. Related to Figure 5.

(A) Representative size-exclusion chromatography elution profile of p3SLR30:RIG-I and OHSLR30:RIG-I complexes using Superdex 200 Increase 10/300 GL column.

(B) Representative 2D class averages from Relion 2D classification. The complex is not treated with ATP.

Figure S14. Cryo-EM analysis of the p3SLR30 internally-bound and the end-bound complexes. Related to Figure 5 and STAR Methods.

(A) Flow chart of cryo-EM data processing.

(B) Representative micrograph.

(C) Representative 2D class averages from Relion 2D classification.

(D-E) Fourier shell correlation (FSC) curve and fourier shell correlation between map and model for the p3SLR30 internally-bound complex (D) and p3SLR30 end-bound complex (E).

(F-G) Euler angle distribution of all particles used in the final map reconstruction for the p3SLR30 internally-bound complex (F) and the p3SLR30 end-bound complex (G).

(H-I) Local resolution display of the p3SLR30 internally-bound complex (H) and the p3SLR30 end-bound complex (I) reconstruction.

(J-K) Representative local densities contoured at 0.025 of the p3SLR30 internally-bound complex (J) and the p3SLR30 end-bound complex (K).

Figure S15. The Hel2 conformation of p3SLR30, OHSLR30 end-bound and internally-bound complexes. Related to Figure 5.

(A) Overview of helicase motifs (blue) in the Hel2 domain in the p3SLR30 end-bound and internally-bound complexes.

(B) Overview of helicase motifs (blue) in the Hel2 domain in the OHSLR30 end-bound and internally-bound complexes.

Figure S16. Cryo-EM analysis of the p3SLR30:RIG-I complex with AMPPNP addition. Related to Figure 5 and STAR Methods.

(A) Flow chart of cryo-EM data processing.

(B) Representative micrograph.

(C) Representative 2D class averages from Relion 2D classification.

(D) Fourier shell correlation (FSC) curve for the p3SLR30:RIG-I complex.

(E) Fourier shell correlation between the refined model and the map.

(F) Euler angle distribution of all particles used in the final map reconstruction.

(G) Local resolution display of p3SLR30:RIG-I complex reconstruction.

(H) Representative local densities contoured at 0.025

Figure S17. Cryo-EM analysis of the OHSLR30 internally-bound and the end-bound complexes. Related to Figure 5 and STAR Methods.

(A) Flow chart of cryo-EM data processing.

(B) Representative micrograph.

(C) Representative 2D class averages from Relion 2D classification.

(D-E) Fourier shell correlation (FSC) curve and fourier shell correlation between map and model for the OHSLR30 internally-bound complex (D) and OHSLR30 end-bound complex (E).

(F-G) Euler angle distribution of all particles used in the final map reconstruction for the OHSLR30 internally-bound complex (F) and the OHSLR30 end-bound complex (G).

(H-I) Local resolution display of the OHSLR30 internally-bound complex (H) and the OHSLR30 end-bound complex (I) reconstruction.

(J-K) Representative local densities contoured at 0.06 of the OHSLR30 internally-bound complex (J) and 0.05 of the OHSLR30 end-bound complex (K).

Figure S18. Distinguishing features of the internally-bound RIG-I complex. Related to Figure 6.

(A) Novel CTD-RNA interactions in the internally-bound RIG-I complex. Corresponding residues in CTD (orange) are rendered as sticks.

(B) Unique conformation of Walker A residues in motif I in the internally-bound complex. In the zoom-in views, Walker A residues in the internally-bound complex, p3dsRNA:RIG-I, p2dsRNA:RIG-I, p1dsRNA:RIG-I and OHdsRNA:RIG-I are highlighted in gray. ADP and residues are rendered as sticks. The Walker A residues in motif I form a loop that accommodates ADP binding, whereas the same residues adopt an alpha-helical conformation in end-bound cryo-EM structures (where ATP is unbound). This distinction is only evident in the set of cryo-EM structures because crystal structures appear to freeze Motif 1 into a loop conformation, regardless

of whether ATP is present or not. Except for the Walker A motif, all other motifs of Hel1 and Hel2 within the internally-bound complex are similar to those observed in the RIG-I structures with p1dsRNA and OHdsRNA (the structures in the HF conformation), further supporting the idea that HF is primed for ATP binding, hydrolysis and RNA release.

(C) Zoom-in view of Hel_{TNT}-RNA interface in the internally-bound complex. The Hel_{TNT} is rendered in blue and N668 in Hel_{TNT} is shown as sticks. Different from structures of RIG-I bound to p1dsRNA and OHdsRNA, in the internally-bound structure, stable folding of Hel2 and the ATPase domains is maintained by sequestering HelTNT along the duplex RNA backbone, rather than within the CTD as in end-bound complexes.

Figure S19. Conformations of the Hel2 domains in the SF2 helicases. Related to Figure 7.

(A-C) Well-folded domains of SF2 helicases corresponding to Hel2 domain in RIG-I. DEAD-box helicase, Mss116p (PDB ID: 3i61) (A); DEAH-box helicase, Prp22p (PDB ID: 6i3p) (B); DECH-box helicase, HCV NS3 (PDB ID: 3o8b) (C). Helicase motifs are highlighted as follows: Hel_{TNT} (blue), motif IVa (orange), V (cyan), Va (blue), Vc (orange) and VI (red).

(D) Well-folded Hel2 domain of RIG-I bound to host RNA.

The multifunctional nature of Hel2 is a distinguishing feature of RIG-I and related proteins. In classical SF2 proteins, Hel2 has a single function, serving as one lobe of the helicase motor domain, which functions together with Hel1 to bind and hydrolyze ATP while coupling the resulting motions with RNA translocation. But RLRs have adapted, enabling Hel2 to assume an additional regulatory function that is controlled by the HelTNT insert.

Table S1. RNA sequences used in this study. Related to Figure 1, 5 and STAR Methods.

RNAs	Sequences
p3dsRNAa	5’ppp-GGACGUACGUUUCGCGACUGUAGA-OH3’
p3dsRNAb	5’ppp-UCUACAGUCGUUCGACGUACGUCC-OH3’
p2dsRNA	5’pp-GGACGUACGUCGCGACGUACGUCC-OH3’
p1dsRNA	5’p-GGACGUACGUCGCGACGUACGUCC-OH3’
OHdsRNA	5’OH-GGACGUACGUCGCGACGUACGUCC-OH3’
p3SLR30	5’ppp-GGAUCGAUCGAUCGAUCGGCAUCGAUCGGCUUCGGCCGAUCGAUGCCGAUCGAUCGAUCGAUCC-OH3’
OHSLR30	5’OH-GGAUCGAUCGAUCGAUCGGCAUCGAUCGGCUUCGGCCGAUCGAUGCCGAUCGAUCGAUCGAUCC-OH3’

Table S2. Cryo-EM data collection, refinement and validation statistics. Related to Figure 1, 5 and STAR Methods.

**Structure:**	OHdsRNA :RIG-I	p1dsRNA :RIG-I	p2dsRNA :RIG-I	p3dsRNA :RIG-I	p3SLR30 :RIG-I+ATP		OHSLR30 :RIG-I+ATP			p3SLR30: RIG-I+ AMPPNP
					Internally-bound	End-bound	Internally-bound 1	Internally-bound 2	End-bound	p3SLR30 :RIG-I
**Data collection and processing:**										
Voltage (KeV)	300	300	300	300	300			200		300
Pixel size (Å)	1.068	1.068	1.068	1.05	1.05			1.149		1.068
Magnification	81,000 ×	81,000 ×	81,000 ×	130,000 ×	130,000 ×			36,000 ×		81,000 ×
Defocus range (μm)	-1.2 ~ -3.0	-1.2 ~ -3.0	-1.2 ~ -3.0	-1.2 ~ -2.7	-1.2 ~ -2.7			-0.8 ~ -2.0		-1.2 ~ -3.0
Total electron exposure (e^-/$Å^2$)	59	59	59	61	68			50		60
Collected micrographs	2838	2460	2586	3480	3417			3663		2744
Initial particle images (no.)	3,357,160	3,005,433	3,354,308	1,996,263	2,188,358			3,635,886		3,097,952
Final particle images (no.)	435,184	624,117	957,706	613,519	366,751	253,073	1,224,061	631,595	386,764	692,833
Symmetry applied	C1	C1	C1	C1	C1	C1	C1	C1	C1	C1
Map resolution (Å)	3.5	3.5	3.2	3.5	3.2	3.7	3.0	2.9	3.0	3.3
FSC threshold	0.143	0.143	0.143	0.143	0.143	0.143	0.143	0.143	0.143	0.143
Local resolution range (Å)	2.3-5.0	2.3-5.0	2.3-5.0	2.3-5.0	2.3-5.0	2.3-5.0	2.5-5.0	2.5-5.0	2.5-5.0	2.3-5.0
Sharpening B-factor ($Å^2$)	-146.58	-189.05	-126.40	-150.00	-82.00	-140.00	-116.23	-114.44	-118.35	-157.95
**Model building and refinement:**										
Initial model used (PDB code)				5F9H	5E3H					
Model resolution (Å)	3.6	3.6	3.2	3.5	3.3	3.8		2.9	3.1	3.4
FSC threshold	0.5	0.5	0.5	0.5	0.5	0.5		0.5	0.5	0.5
Model composition										
Non-hydrogen atoms	5989	5986	5865	5753	6052	5601		6170	6058	5601
Protein residues	682	680	666	647	670	635		669	682	635
RNA residues	24	24	24	24	30	22		36	26	22
Ligands: ADP/Mg/Zn	-/-/1	-/-/1	-/-/1	-/-/1	1/1/1	-/-/1		1/1/1	1/1/1	-/-/1
B factors ($Å^2$)										
Protein	77.85	112.92	74.87	79.74	71.62	83.37		16.31	36.38	43.55
RNA	38.16	33.91	33.35	39.29	49.07	33.27		20.42	35.20	28.52
Ligand	9.61	37.48	20.79	24.04	68.91	35.48		20.89	50.28	26.85
R.m.s. deviations										
Bond lengths (Å)	0.011	0.011	0.011	0.011	0.011	0.011		0.002	0.002	0.002
Bond angles (°)	1.281	1.270	1.245	1.221	1.248	1.198		0.506	0.456	0.446
Validation										
Molprobity score	1.91	1.92	1.91	1.83	1.66	1.89		1.76	1.93	1.89
All-atom clashscore	8.15	8.50	8.08	6.37	5.75	7.10		6.25	7.33	8.74
Rotamer outliers (%)	0.82	0.66	0.84	0.17	0.84	0.00		0.50	1.00	0.70
EMRinger score	2.21	2.02	2.34	2.39	2.46	1.82		3.49	2.76	1.81
Ramachandran plot										
Favored (%)	92.50	92.75	92.40	92.18	95.05	91.71		93.83	93.24	95.53
Allowed (%)	7.50	6.80	4.60	7.67	4.95	8.29		6.17	6.76	4.47
Outliers (%)	0.00	0.44	0.00	0.16	0.00	0.00		0.00	0.00	0.00

Table S3. Polar interactions between RIG-I and RNA in p3dsRNA:RIG-I, p2dsRNA:RIG-I, p1dsRNA:RIG-I, OHdsRNA:RIG-I and internally-bound complex. Related to Figure 1, 2, 3, 5 and STAR Methods.

Interactions	5'-ppp (Å)	5'-pp (Å)	5'-p (Å)	5'-OH (Å)	Stem (Å)
N298O-U'22O2'	3.94	3.43	3.02	4.21	3.10 (G52)
N298OD1/ND2-U'22O2'	4.16	3.97	3.98	2.95	4.09 (G52)
I300N-C'23OP1	2.87	2.73	2.95	2.98	2.74 (A53)
S325OG-C'24OP1	3.75	3.39	3.60	4.52	4.27 (U54)
G326N-C'24OP1	3.08	3.07	2.92	3.34	2.67 (U54)
T347OG1-C'24OP1	3.20	2.74	3.08	4.08	3.68 (U54)
N353ND2-C'24O2'	3.12	3.53	3.35	3.32	2.80 (U54)
S378OG-6UOP1	4.20	4.21	3.63	3.98	4.42 (U16)
K379NZ-6UOP1	2.91	3.15	2.91	2.68	3.24 (U16)
K379NZ-6UOP2	3.74	3.98	3.56	3.39	4.18 (U16)
K379O-5GOP1	2.60	2.31	2.29	4.96	5.02 (A15)
Q380OE1/NE2-4COP1	4.27	4.74	3.29	3.50	6.14 (G14)
Q380N-5GOP1	4.07	3.89	4.21	3.03	3.12 (A15)
H381NE2/ND1-4CO2'	4.32	4.45	4.15	2.97	4.48 (G14)
Q498NE2/OE1-C11O2'	-	2.78	-	4.25	-
I499O-C11OP1	3.52 (U11)	6.52	3.34	7.42	-
Q500OE1/NE2-C11OP1	5.08 (U11)	6.61	3.43	3.74	-
Q507OE1-C8O2'	2.60	2.54	2.84	2.96	2.75 (G18)
K508NZ-U10OP1	4.53	3.83 (C11)	4.59	4.50	4.14 (C20)
E510OE1/OE2-U'18O2'	3.05	3.04	3.08	3.10	3.90 (G48)
K518NZ-G'17OP1	3.10	2.70	4.70 (U'18)	4.21 (U'18)	4.74 (G48)
R546NH2-A'19OP1	3.21	3.19	3.20	3.08	3.85 (A49)
V718O-A7O2'	3.98	3.34	3.78	3.82	4.16 (C17)
N720OD1/ND2-A7OP1	-	3.02	3.75	4.32	4.21 (C17)
K750NZ-C8OP1	3.76	3.41	4.53	3.14	4.31 (G18)
R637N-C'20OP1	3.89	3.50	3.70	3.54	3.88 (U50)
R637NH1-G'21OP2	3.64	5.75	3.49	2.63	-
R637NH2-C'20OP1/OP2	3.86	4.19	4.03	3.54	3.03 (C51)
T662O-G'21OP1	3.13	2.78	2.58	2.84	4.86 (G52)
R664N-U'22OP2	-	3.63	3.47	3.55	2.83 (G52)
R664NH2-U'22OP1	-	-	3.66	3.76	2.62 (NH1-A53OP1)
R664NH2-C'23OP2	-	-	3.26	3.23	3.11 (NH2-A53OP2)
G665N-U'22OP2	-	3.12	2.84	3.45	-
Q678OE1/NE2-U'22OP1	-	3.47	4.21	4.69	3.93 (G52)
T697OG1-G'21OP1	4.99	5.41	2.55	3.66	4.46 (C51)
T697O-C'20O2'	4.81	4.33	3.50	6.34	3.61 (U50)
S698OG-C'20O2'	4.01	3.04	3.71	4.27	2.95 (U50)
H847NE2-β(P)O2B	3.35	3.53	-	-	-
K858NZ-β(P)O1B	3.37	2.91	-	-	-
K861NZ-β(P)O2B	2.80	3.64	-	-	-
K861NZ-α(P)O2A	2.75	2.74	3.59	-	-
K888NZ-α(P)O2A	3.75	3.66	3.37	-	-
K888NZ-α(P)O1A	3.21	4.19	3.71	-	-
C829SG-G2O2'	3.66	3.96	3.29	4.28	3.89 (G56)
N668ND2-G1O5'	-	-	-	3.32	-
E827OE2-G56O2'	-	-	-	-	3.10
K878NZ-C59OP1	-	-	-	-	4.01
K880N-U58OP1	-	-	-	-	3.48
